# Supplementary material for: Cortical somatostatin long-range projection neurons and interneurons exhibit divergent developmental trajectories
Source: Neuron. Author manuscript; Available in PMC 2025 Oct 11. (PMC7618239; doi:10.1016/j.neuron.2023.11.013)
Supplement: Supplemental Information — can be found online at https://doi.org/10.1016/j.neuron.2023.11.013. [file EMS209514-supplement-Supplemental_Information.zip › 1-s2.0-S0896627323008875-mmc3.pdf]

Neuron

Article

Cortical somatostatin long-range projection neurons and interneurons exhibit divergent developmental trajectories

Graphical abstract

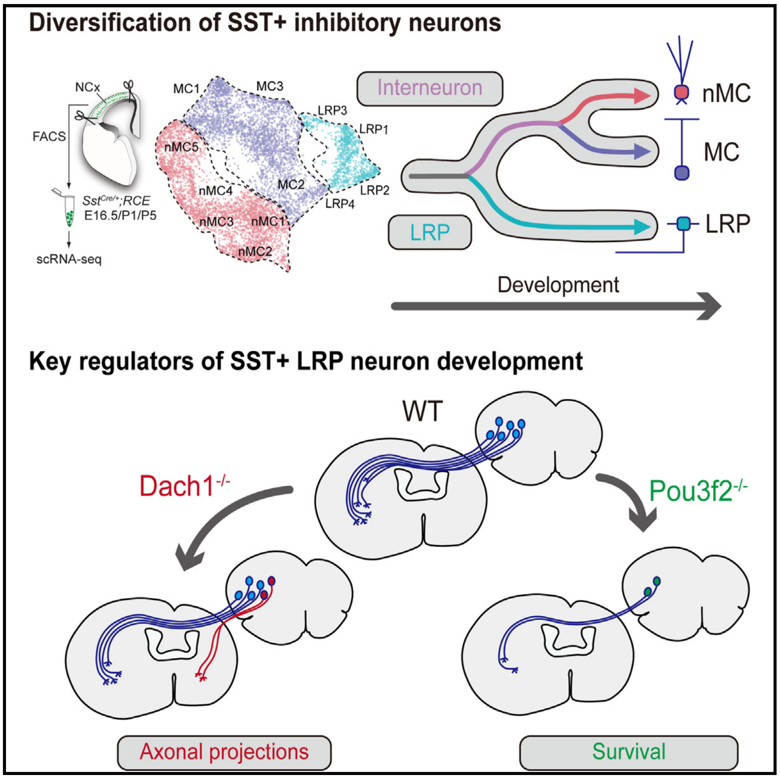

Highlights

- Diversity among cortical inhibitory SST+ neurons emerges early in development
- The timing of interneuron diversification varies among different SST+ neurons
- Long-range GABAergic neurons implement unique molecular programs of development

Authors

Josephine Fisher, Marieke Verhagen, Zhen Long, ..., Oscar Marín, Da Mi, Lynette Lim

Correspondence

oscar.marin@kcl.ac.uk (O.M.),
mida@tsinghua.edu.cn (D.M.),
lynette.lim@kuleuven.vib.be (L.L.)

In brief

Fisher et al. reveal that cortical GABAergic neurons expressing somatostatin are highly diverse during embryonic development. However, the timing of cell fate acquisition among these cells varies depending on their identity. Fisher and colleagues also identify distinct molecular programs governing the diversification of GABAergic long-range projection neurons and interneurons.

Fisher et al., 2024, Neuron *112*, 558–573
February 21, 2024 © 2023 The Authors. Published by Elsevier Inc.
https://doi.org/10.1016/j.neuron.2023.11.013

CellPress
OPEN ACCESS

Neuron

Article

Cortical somatostatin long-range projection neurons and interneurons exhibit divergent developmental trajectories

Josephine Fisher,[1,2,6] Marieke Verhagen,[3,4,6] Zhen Long,[5,6] Monika Moissidis,[1,2,6] Yiming Yan,[5,6] Chenyi He,[5] Jingyu Wang,[5] Elia Micoli,[3,4] Clara Milían Alastruey,[3,4] Rani Moors,[3,4] Oscar Marín,[1,2,7,8,*] Da Mi,[5,7,*] and Lynette Lim[3,4,7,*]
[1]Centre for Developmental Neurobiology, Institute of Psychiatry, Psychology and Neuroscience, King's College London, SE1 1UL London, UK
[2]MRC Centre for Neurodevelopmental Disorders, King's College London, SE1 1UL, London, UK
[3]VIB Center for Brain and Disease, 3000 Leuven, Belgium
[4]Department of Neurosciences, Katholieke Universiteit (KU) Leuven, 3000 Leuven, Belgium
[5]State Key Laboratory of Membrane Biology, Tsinghua-Peking Center for Life Sciences, IDG/McGovern Institute for Brain Research, School of Life Sciences, Tsinghua University, Beijing 100084, China
[6]These authors contributed equally
[7]These authors contributed equally
[8]Lead contact
*Correspondence: oscar.marin@kcl.ac.uk (O.M.), mida@tsinghua.edu.cn (D.M.), lynette.lim@kuleuven.vib.be (L.L.)
https://doi.org/10.1016/j.neuron.2023.11.013

SUMMARY

The mammalian cerebral cortex contains an extraordinary diversity of cell types that emerge by implementing different developmental programs. Delineating when and how cellular diversification occurs is particularly challenging for cortical inhibitory neurons because they represent a small proportion of all cortical cells and have a protracted development. Here, we combine single-cell RNA sequencing and spatial transcriptomics to characterize the emergence of neuronal diversity among somatostatin-expressing (SST+) cells in mice. We found that SST+ inhibitory neurons segregate during embryonic stages into long-range projection (LRP) neurons and two types of interneurons, Martinotti cells and non-Martinotti cells, following distinct developmental trajectories. Two main subtypes of LRP neurons and several subtypes of interneurons are readily distinguishable in the embryo, although interneuron diversity is likely refined during early postnatal life. Our results suggest that the timing for cellular diversification is unique for different subtypes of SST+ neurons and particularly divergent for LRP neurons and interneurons.

INTRODUCTION

Cortical γ-aminobutyric acid-containing (GABAergic) inhibitory neurons constitute one of the most diverse cellular populations in the brain. The most comprehensive catalog of neuronal diversity based on transcriptomic information suggests that the adult cerebral cortex of mice, marmosets, and humans may contain over 100 subtypes of inhibitory neurons.[1] Even more stringent censuses that collectively consider the morphological, electrophysiological, and molecular properties of these cells identify some 30 different subtypes of GABAergic neurons in the neocortex (NCx) and hippocampus.[2,3] When and how this extensive repertoire of inhibitory neurons emerges during development remains unclear.

Several studies over the past decade have revealed that the diversity of cortical inhibitory neurons emerges during embryonic development. For instance, transcriptomic analyses have found that many newborn neurons have similar gene expression profiles to those found among adult neurons,[4–8] which suggests that the identity of cortical inhibitory neurons may be established shortly after neurogenesis. However, there is a large discrepancy between the number of different subtypes of GABAergic neurons described in the adult and the developing cerebral cortex. This difference can no longer be attributed to undersampling issues in developmental studies because some of the most recent observations derive from analyzing more than 40,000 cells from the ganglionic eminences,[5,7,8] the embryonic origin of cortical inhibitory neurons. Instead, the inconsistency may reflect that the diversification of some GABAergic inhibitory neurons may occur at different times during development.[9–12]

The large diversity of cortical inhibitory neurons can be organized into five major subclasses based on common biochemical characteristics and gene expression: parvalbumin-expressing (PV+) interneurons, somatostatin-expressing (SST+) neurons, vasoactive intestinal peptide-expressing (VIP+) bipolar cells, *Sncg*-expressing basket cells, and *Lamp5*-expressing neurogliaform cells.[2,13] SST+ cells are the most diverse among cortical

 © 2023 The Authors. Published by Elsevier Inc.
This is an open access article under the CC BY license (http://creativecommons.org/licenses/by/4.0/).

CellPress
OPEN ACCESS

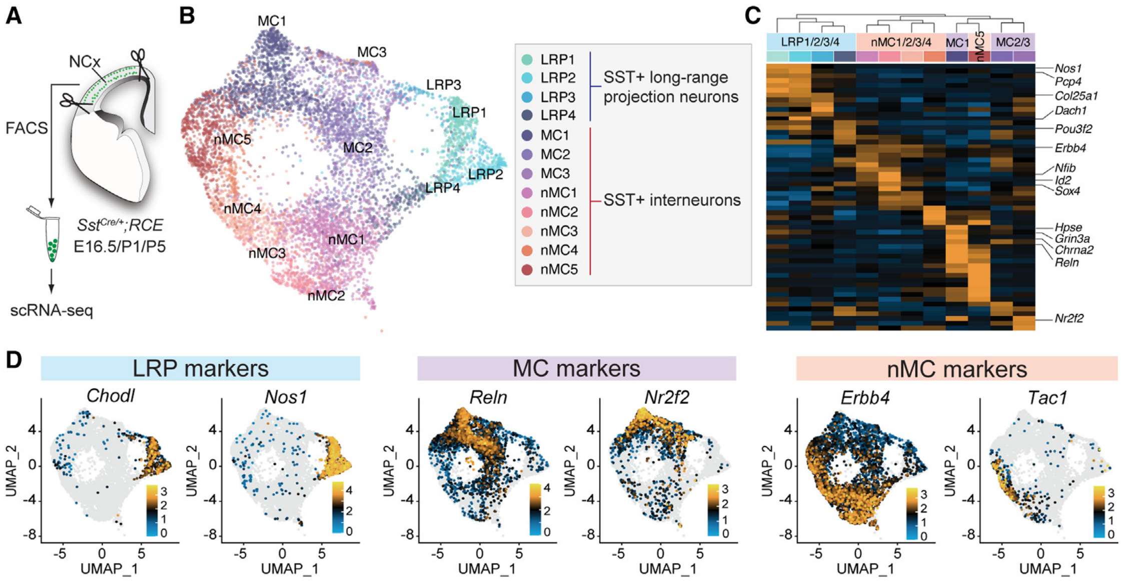

Figure 1. The diversity of cortical SST+ inhibitory neurons emerges in early development

(A) Schema of experimental design. Enrichment of SST+ neurons by dissection of the neocortex (NCx) from *Sst*$^{Cre/+}$*;RCE* mice followed by FACS.

(B) Integration of SST+ neurons from E16.5, P1, and P5 and visualization by uniform manifold approximation and projection (UMAP). The annotation of cell clusters was based on marker expression.

(C) Heatmap illustrating differentially expressed genes (DEGs) enriched in each cluster.

(D) Examples of gene expression of markers for LRP neurons, MCs, and nMCs visualized by UMAP.

GABAergic neurons. In the adult mouse NCx, thirteen subtypes of SST+ inhibitory neurons with distinctive morpho-electrical and transcriptomic profiles have been identified using Patch-seq. They include long-range projecting (LRP) neurons and two main types of SST+ interneurons, Martinotti cells (MCs) and non-MCs (nMCs).[2] SST+ LRP neurons are unique among neocortical GABAergic neurons because their axons extend across different cortical regions.[14,15] SST+ LRP neurons characteristically express *Chodl* and are among the most conserved cell types in the mammalian NCx,[1] which suggests that they play an essential role in cortical function. For instance, SST+ LRP neurons may regulate slow-wave oscillations during non-rapid eye movement sleep.[16,17] Although SST+ LRP neurons and SST+ interneurons seem to derive from the same region of the embryonic telencephalon,[18] the mechanisms driving the diversification of this subclass of cortical GABAergic neurons are unknown.

Here, we used SST+ neurons as a model to study the timing and molecular regulation of the diversification of cortical GABAergic inhibitory neurons in the mouse. We used single-cell RNA sequencing (scRNA-seq) to analyze gene expression in over 7,000 SST+ GABAergic inhibitory neurons isolated from three different developmental stages spanning embryonic day (E) 16.5 and postnatal day (P) 5. Our results uncover unique, cell-type-specific molecular programs governing the diversification and maturation of cortical SST+ GABAergic inhibitory neurons, identify transcription factors driving the unique development of SST+ LRP neurons, and suggest the timing of cell-fate decisions among GABAergic neurons varies depending on cell identity.

RESULTS

The transcriptional diversity of SST+ neurons emerges during development

We took advantage of the fact that *Sst* mRNA is expressed very early in GABAergic neurons of the telencephalon[19] to isolate SST+ neurons. To this end, we crossed *Sst*$^{Cre/+}$mice with a Rosa26-CAG-boosted eGFP reporter line (RCE). We then dissected the NCx of *Sst*$^{Cre/+}$*;RCE* mice at E16.5, P1, and P5 and obtained cell populations highly enriched in SST+ neurons using fluorescent-activated cell sorting (FACS). We then examined gene expression in individual cells using 10× Genomics Chromium sequencing (Figure 1A). We performed RNA-seq on 12,324 cells from three developmental stages, which were then subjected to quality control (see STAR Methods). This process rendered 9,154 cells with an average of 4,403 UMI per cell and a median of 2,170 genes per cell.

We performed cross-dataset anchor analysis to integrate the scRNA-seq datasets from the three developmental stages (Figure S1A). Using an iterative hierarchical clustering approach,[20] we found 17 distinct clusters of neurons (Figure S1B). Most groups (12/16) exhibit enriched expression of cellular markers, enabling their annotation into one of the three main types of cortical SST+ GABAergic neurons: LRP neurons, MCs, and nMCs (Figure S1C). Another cluster representing a small fraction (0.9%) of neurons was identified by the expression of *Meis2* (Figures S1B and S1C). This group corresponds to a previously identified population of interneurons that derives from the caudal ganglionic eminence and is primarily confined to deep cortical layers and the white matter.[21] These cells are

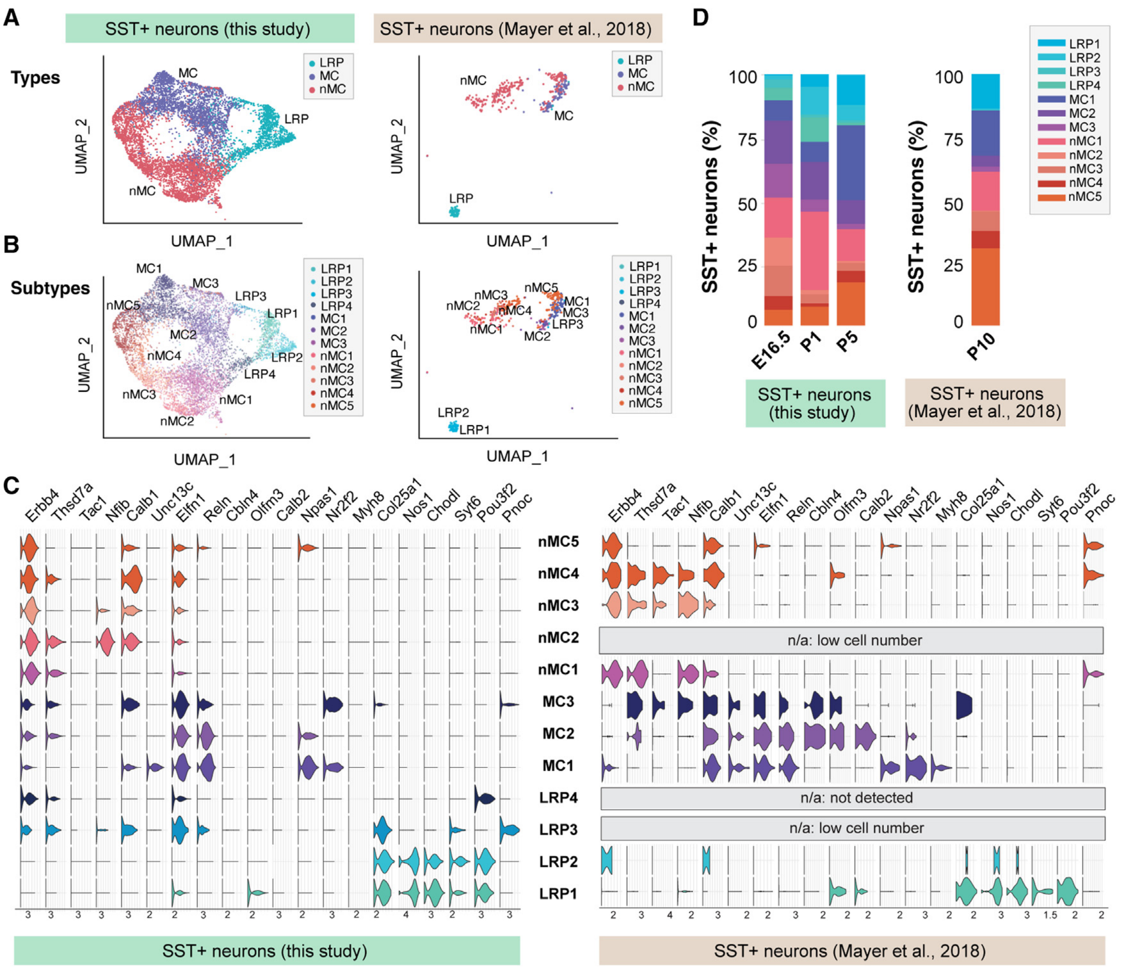

Figure 2. SST+ subtype diversity in previously published datasets

(A and B) Clustering of cortical SST+ cells into types (A) and subtypes (B) in the E16.5, P1, and P5 datasets (this study) and the P10 dataset.[4] In the latter case, clusters were reannotated using the classifier models described in this study.

(C) Violin plots of selected cell-type markers comparing SST+ cells in the E16.5, P1, and P5 datasets (this study) and the P10 dataset.[4]

(D) Distribution of each subtype in the E16.5, P1, and P5 datasets (this study) and the P10 dataset.[4]

labeled in *Sst*$^{Cre/+}$;*RCE* mice, perhaps due to the transient expression of *Sst* (Figures S1D and S1E). We also detected a small fraction of cells that co-expressed PV (Figure S2). Finally, we also observed four small groups of cells with signs of endoplasmic reticulum stress ("stressed" clusters), including enrichment in heat-shock genes and lower average UMIs and number of genes than other neurons (Figure S1F). The Meis2 and stressed clusters were discarded from further analyses.

The 12 clusters of developing cortical SST+ neurons comprise four groups of LRP neurons (LRP1–4) and eight groups of SST+ interneurons, including three groups of MCs (MC1–3) and five groups of nMCs (nMC1–5) (Figure 1B), all characterized by unique patterns of gene expression (Figures 1C and 1D; Table S1). We generated random forest models to evaluate the accuracy and robustness of this cell classification for the main types of SST neurons (LRP neurons, MCs, and nMCs) and individual clusters. We observed 91% accuracy in classifying the three main types and accuracies ranging from 75% to 91% for individual clusters, all with very low false positive rates (Figure S1G). These data revealed that up to 12 potentially distinct subtypes of SST+ neurons could be detected with high confidence in the developing cortex.

Previous studies in mice have not identified such a remarkable diversity among SST+ neurons in the early postnatal cortex.[4] Because clustering and classification methods differ significantly among studies, we reasoned that if our classifier models were sufficiently robust, we should be able to detect similar levels of neuronal diversity among SST+ neurons in previously published datasets. To test this hypothesis, we used a published scRNA-seq dataset of FACS-enriched cortical inhibitory neurons from P10 mice containing 540 SST+ neurons.[4] Label transfer allowed the identification of the three main types of SST+ neurons in this small dataset (Figure 2A). Remarkably, we also identified 11 of the 12 clusters of SST+ neurons found in our dataset, with LRP4 neurons not accounted for (Figures 2B and 2C). In addition, the relative fraction of cells detected for each cluster

CellPress
OPEN ACCESS

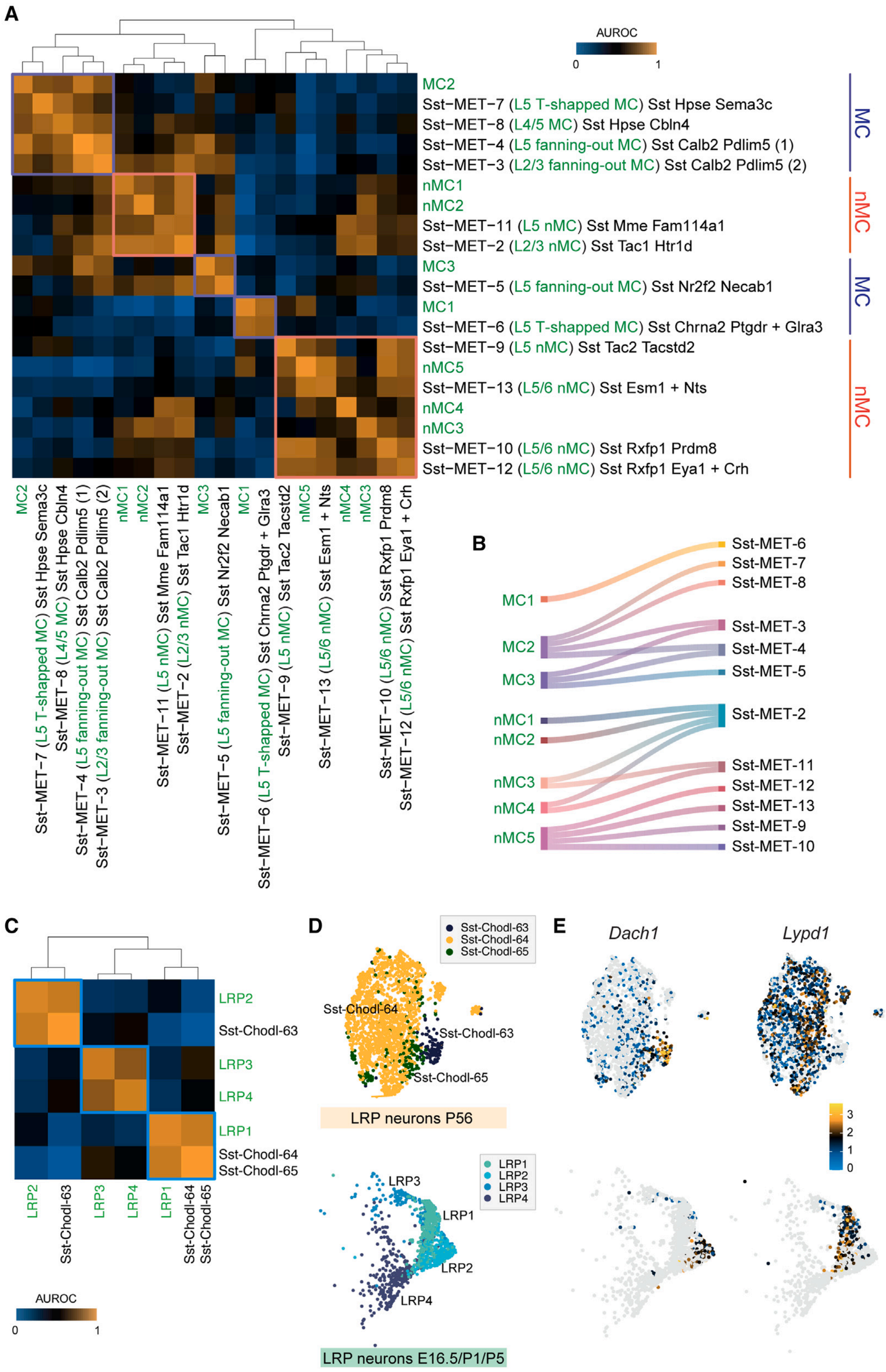

(legend on next page)

was similar in other developmental stages (Figure 2D). This observation suggested that these groups of cells represent relatively stable cell identities from E16.5 to P10. To verify that the gene expression patterns observed in these experiments were not flawed by sorting live cells, we fixed dissociated P5 cells in 4% PFA and performed scRNA-seq using 10× Genomic Chromium Fixed RNA Profiling (see STAR Methods). The results of this experiment revealed a very similar degree of diversity among SST+ cells than with the live cells (Figure S3), which suggests that the live cells isolated via FACS accurately represent cortical SST+ interneurons. These results reinforced the notion that cell diversity among SST+ inhibitory neurons emerges during early cortical development in mice.

Identification of SST+ subtypes during early postnatal development

We used MetaNeighbor analysis[22] to determine whether the transcriptionally diverse groups of developing SST+ neurons observed in our dataset at P5 correspond to specific subtypes of adult SST+ neurons. We identified adult interneurons using a classification that integrates their morphological, electrophysiological, and transcriptomic (MET) properties.[2] Because this is a small dataset, we combined it with a larger single-cell transcriptomic dataset[20] that can be linked to the MET cell type classification (Figures S4A and S4B).

Our initial observations show that the three main types of adult cortical SST+ inhibitory neurons (LRP, MC, and nMC) are easily discernible during development. At the level of specific subtypes, however, not every group identified during development maps to a single adult subtype identity. For instance, although the MC1 transcriptional profile in the developmental dataset aligns well with Sst-MET-6, which corresponds to deep-layer alpha-2 T-shaped MCs,[23] MC3 matches several subtypes of fanning-out MCs (Sst-MET-3, Sst-MET-4, and Sst-MET-5) that occupy different layers in the adult cortex[24,25] (Figures 3A and 3B). The developmental profile MC2 exhibits transcriptional similarities with both subtypes of MCs. The correspondence between developmental profiles and adult subtypes is less clear for nMCs than for MCs. For example, four developmental profiles (nMC1, nMC2, nMC3, and nMC4) are transcriptionally similar to adult Sst-MET-2, which include SST+ interneurons with fast-spiking-like firing properties.[26–28] Conversely, most nMC developmental profiles match multiple subtypes of nMCs (Figures 3A and 3B). In particular, nMC5 is transcriptionally similar to four distinct deep-layer nMC subtypes (Sst-MET-9, -10, -12, and -13) between P5 and adult stages (Figures 3A and 3B). These observations suggest that the diversification of MCs into different subtypes precedes the differentiation of nMCs.

Because the MET classification renders a single subtype of SST+ LRP neurons (Sst-MET-1), we used a more recent single-cell transcriptomic analysis of the adult mouse cortex that identified three transcriptionally distinct populations of adult SST+ LRP cells (Sst-Chodl-63, Sst-Chodl-64, and Sst-Chodl-65).[29] Using MetaNeighbor analysis to explore relationships between these identities and the developmental LRP profiles, we found a clear correspondence between two distinct cell clusters at both stages: LRP1 matches the largely overlapping adult Sst-Chodl-64 and Sst-Chodl-65, whereas LRP2 aligns with the adult subtype Sst-Chodl-63 (Figure 3C). By contrast, we found that LRP3 and LRP4 do not match any adult LRP cells (Sst-Chodl) (Figure 3C). Because LRP3 and LRP4 seem to have a mixed transcriptional identity, bridging the interneuron and long projection neuron profiles (Figure 1B), we compared their transcriptional profile more broadly with other adult SST+ interneurons. We found that LRP3 aligns well with Sst-Etv1-87 (Figure S4C), a neuronal population almost exclusively found in adults in the hippocampal formation.[29] Next, we repeated the MetaNeighbor analysis with the LRP1, LRP2, LRP3, and LRP4 clusters from our dataset along with the adult Sst-Chodl-63, Sst-Chodl-64, Sst-Chodl-65, and Sst-Etv1-87. This analysis confirmed that LRP3 and LRP4 have the highest similarity with Sst-Etv1-87 (Figure S4D). Additionally, when we examined the number of LRP neurons at each developmental stage, we found that LRP3 cells were almost exclusively present in E16.5 samples (Figure S4E), which is consistent with the notion that we could only isolate these prospective hippocampal neurons from the developing NCx during their tangential migration toward the hippocampus.

We identified embryonic markers that remain enriched into adulthood for the two subtypes of LRP neurons that most clearly correspond to Sst-Chodl subtypes in the adult (Figures 3D and 3E). Specifically, we found that *Dach1* is highly enriched in LRP2 neurons during development and in Sst-Chodl-63 in the adult cortex, whereas *Lypd1* is a marker of developing LRP1 cells and adult Sst-Chodl-64 and Sst-Chodl-65 neurons. These observations suggest that in contrast to many interneuron subtypes, subtype diversity among SST+ LRP inhibitory neurons is readily evident during early postnatal development.

Divergent developmental trajectories among SST+ inhibitory neurons

Previous studies have shown that, regardless of cell type identity, cortical pyramidal cells use broadly conserved differentiation programs to drive newborn neurons toward their final identity.[30] Because cortical inhibitory neurons exhibit a dramatically different maturation process,[18] we investigated whether a similar principle applies to the differentiation of SST+ inhibitory neurons.

Figure 3. Alignment of developmental SST+ cell types and subtypes to their corresponding adult counterparts

(A) Heatmap illustrating transcriptomic similarities (AUROC values) between the developmental SST+ cell clusters identified in this study and adult SST+ cell clusters, as defined in Mayer et al.[2] The adult dataset includes SST+ cells from Tasic et al.,[20] with clear correspondence to the MET subtypes defined in Mayer et al.[2]

(B) River plot of adult Sst-MET types with the developmental SST+ cell clusters identified in this study using an AUROC value larger than 0.75.

(C) Heatmap illustrating AUROC values between developmental and adult populations of LRP neurons, attained through MetaNeighbor analysis using the intersection of DEGs between groups.

(D) UMAP of LRP cells in the adult (cells from Yao et al.[29]) and developing mouse neocortex.

(E) UMAP depicting the expression of LRP neuron marker genes in the adult (top) and developing (bottom) mouse neocortex.

CellPress
OPEN ACCESS

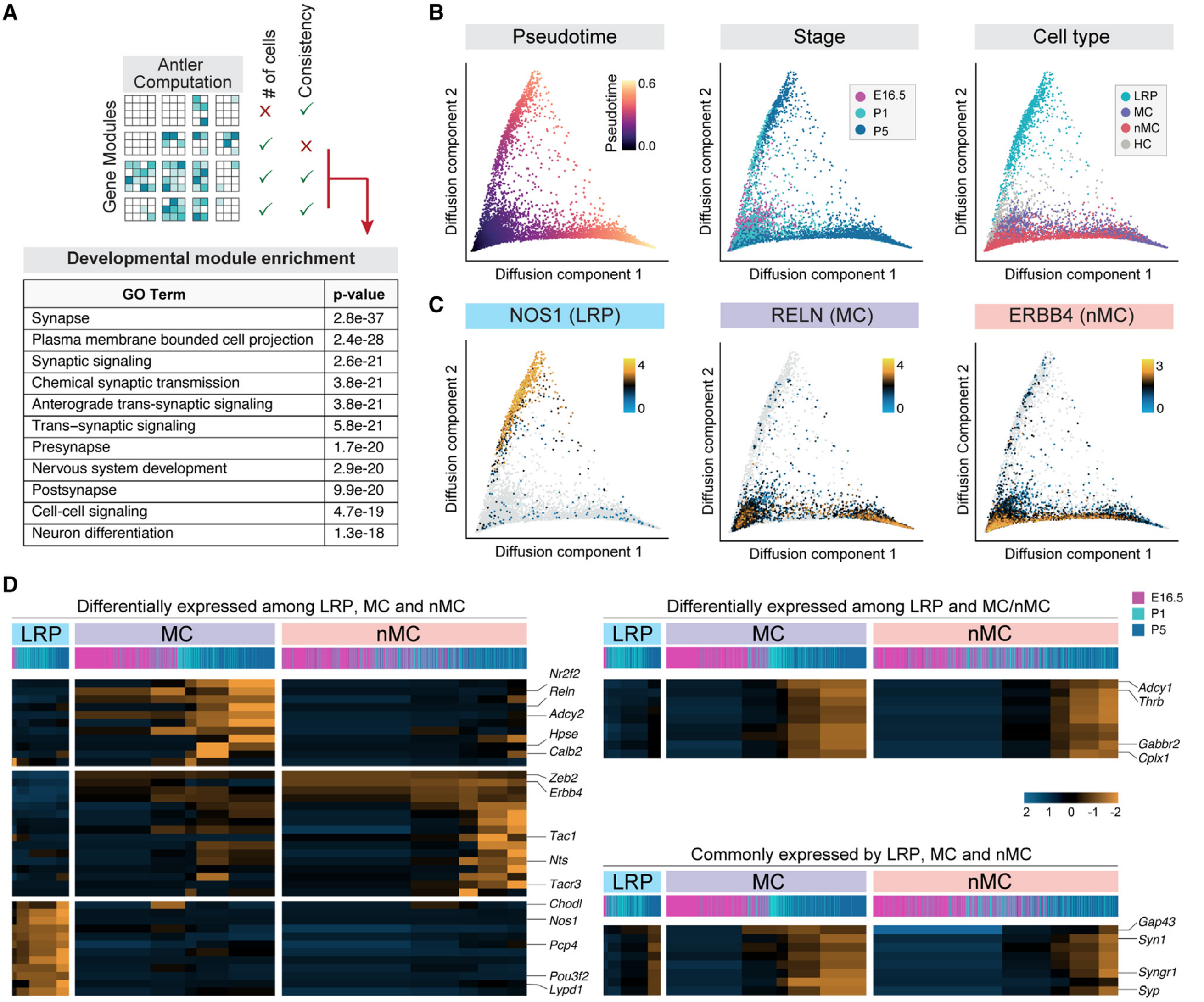

GO Term	p-value
Synapse	2.8e-37
Plasma membrane bounded cell projection	2.4e-28
Synaptic signaling	2.6e-21
Chemical synaptic transmission	3.8e-21
Anterograde trans-synaptic signaling	3.8e-21
Trans–synaptic signaling	5.8e-21
Presynapse	1.7e-20
Nervous system development	2.9e-20
Postsynapse	9.9e-20
Cell-cell signaling	4.7e-19
Neuron differentiation	1.3e-18

Figure 4. Differential gene expression delineates distinct developmental trajectories for SST+ GABAergic neurons

(A) Schematic drawing illustrating gene module filtering for identifying developmentally relevant genes. Genes were grouped into modules by hierarchical clustering and then iteratively discarded based on quality criteria. GO term functional enrichment revealed that the developmental gene module (Table S2) relates to synapse formation and neuronal differentiation.

(B) UMAP plots illustrating pseudotime value, sample age, and cell type.

(C) Gene expression over pseudotime trajectories, plotted for a selection of gene markers for the three main SST+ cell types. *Nos1*, *Reln*, and *Erbb4* are most enriched along the LRP, MC, and nMC branches.

(D) Heatmaps depicting genes changing during development that are (1) differentially expressed among cell types (i.e., branch-specific genes), (2) differentially expressed between LRP neurons and interneurons, and (3) shared by all SST+ GABAergic neurons.

To this end, we first organized genes into distinct modules based on their correlated expression in SST+ neurons and then identified the module that changes most significantly during development. We reasoned that this module should contain transcripts likely linked to cellular maturation. To this end, we performed gene module computations using ANTLER (see STAR Methods), in which genes are clustered iteratively and filtered by quality criteria to generate coherent modules describing gene function (Figure 4A). Following these criteria, we defined a developmental gene module containing 159 genes explaining differences in the maturation stage of the cells (Table S2). We performed gene ontology (GO) enrichment analysis and found that most of the genes in the developmental gene module were related to synapses, axons, and neuronal development (Figure 4A). We then used the developmental gene module to calculate diffusion pseudotime from transition probabilities in a diffusion map space and related the computed pseudotime to the sample stage and the distinct types of SST+ neurons (Figure 4B), with LRP3 and LRP4 now reannotated as hippocampal interneurons. We found three main branches in the pseudotime that align with three main

types of SST+ neurons (LRP neurons, MCs, and nMCs). The LRP, MC, and nMC branches are characterized by distinctive gene expression patterns (Figures 4B and 4C). Most strikingly, we observed that the branch corresponding to LRP neurons is largely divergent from the interneuron branches (Figures 4B and 4C).

We next identified genes that are (1) differentially expressed among LRP neurons, MCs, and nMCs; (2) differentially expressed between LRP neurons and interneurons (MCs and nMCs); and (3) commonly expressed by all the developing SST+ neurons, and mapped their expression along their corresponding pseudotime trajectories (Figure 4D; Table S3). Although all SST+ neurons share standard molecular programs during their maturation, these analyses revealed that each type progressively unfolds the expression of a unique combination of genes that defines its developmental trajectory. Such a distinctive program is particularly evident for SST+ LRP neurons, which diverge from SST+ interneurons already at embryonic stages.

Laminar allocation of SST+ neurons and cell identity

We carried out spatial transcriptomics of the mouse NCx at P5 to explore the correlation between cell identity and laminar allocation among SST+ neurons. To this end, we used the Molecular Cartography platform (Resolve Biosciences), a multiplex spatial analysis technology based on single-molecule fluorescence *in situ* hybridization methods with barcoded probe labeling (Figures 5A and 5B). Using this method, we surveyed the expression of 94 genes (Table S4) with single-cell resolution and a capturing efficiency of 80%. These genes were selected among those differentially expressed by the 12 subtypes of SST+ neurons detected in our scRNA-seq dataset (Figure 1). We then used DAPI staining of individual nuclei to segment individual cells and obtained the gene expression profile and laminar location of over 40,000 cells from 4 cortical tissue sections.

To examine the spatial distribution of SST+ neurons, we filtered the dataset to exclude all other cell populations by gating the expression of *Sst*, *Gad1*, *Gad2*, and *Lhx6* (Figure 5A). We then carried forward the intersection of these sets, retaining only those clusters where all markers were differentially expressed. We obtained 2,319 SST+ neurons using this approach, representing about 5% of the segmented cells. We unbiasedly clustered these cells into 8 groups, based on gene expression, which we then annotated using marker genes (e.g., *Chodl* for LRP neurons; Figure S5A). Most SST+ cell types defined through spatial transcriptomics correlate highly with the SST+ cell types obtained in our scRNA-seq profile (AUROC > 0.8; Figure S5B). Based on this finding, we reannotated the SST+ cell clusters from the spatial transcriptomic dataset to match the corresponding subtype identities defined by the scRNA-seq dataset (Figure 5C). For example, we found that the spatial cluster Sst-sp1 correlates with the LRP1 and LRP2 populations in the scRNA-seq dataset, Sst-sp4 with MC2 and MC3, Sst-sp5 with MC1, Sst-sp3 with nMC1, nMC2, and nMC3, and Sst-sp2 with nMC5 (Figure S5A). This process allowed determining the laminar distribution of the various types of SST+ neurons at P5 (Figure 5D).

To compare the laminar allocation of SST+ cell types at P5 with the corresponding adult MET types,[2] we divided the cells in each cluster based on their location in superficial (layers 1–4) and deep layers (layers 5 and 6). We found that the distribution of deep-layer SST+ interneurons such as MC1 and nMC3/5 (corresponding to adult Sst-MET-6 and Sst-MET-9/10/13, respectively) was already established at P5 (Figure 5E). By contrast, the relative distribution of superficial layer subtypes in MC2/3 and nMC1/2/3 (adult Sst-MET-3 and Sst-MET-2, respectively) significantly differed between P5 and P56 (Figure 5E). This was also the case for LRP1/2, which at P5 were almost exclusively confined to deep layers (>90% of cells). By contrast, a more significant fraction is present in the superficial layers of the NCx at P56 (Figure 5E). Thus, SST+ subtypes located in deep layers of the NCx have reached their final position by P5, whereas subtypes occupying superficial layers have not. In conclusion, at least for the timing of laminar allocation, deep-layer SST+ cells arrive at their final laminar before SST+ subtypes that populate superficial layers independently of cell-type identity.

Molecular control of SST+ LRP neuron development

Although the molecular programs regulating the development of SST+ interneurons are beginning to be elucidated,[18] very little is known about the transcription factors controlling the development of SST+ LRP neurons. Because our previous analyses suggested that SST+ LRP neurons and SST+ interneurons follow vastly divergent developmental trajectories, we searched for differentially expressed genes (DEGs) among these cells (https://fisherj.shinyapps.io/sst-in-diversity/; Table S3). We then performed STRING network analysis[31] with the 111 genes enriched in LRP neurons (Figure S6A). This analysis revealed an active node enriched in transcription factors such as *Zeb1*, *Sox2*, *Tshz2*, *Pou3f2*, *Sox1*, and *Dach1* (Figure S6A). Among these genes, we selected those encoding the transcription factors Dach1 and Pou3f2 (also known as Brn2) for functional validation because they exhibited the highest expression in the developing LRP neurons and relatively limited expression in SST+ interneurons (Figure S6B). Dach1 appears to be mostly restricted to LRP2, whereas Pou3f2 seems to be expressed in most LRP cells (Figure S6B).

To explore the role of Dach1 in developing SST+ LRP neurons, we first validated its expression using immunohistochemistry. SST+ LRP neurons can be marked by their high levels of expression of nitric oxide synthase 1 (NOS1)[32,33] (Figures S6C and S6D). Consistent with our scRNA-seq analyses, we found that Dach1 is exclusively expressed in a relatively small fraction of NOS1+ LRP neurons at P21 (Figures S6E and S6F), most frequently found in L6 in all cortical areas examined (Figure S6G). We generated conditional mutant mice in which Dach1 is exclusively removed from SST+ neurons. We found that the density of SST+ LRP neurons at P21 was similar between control and $Sst^{Cre/+}$;$Dach1^{fl/fl}$ mice across all cortical areas examined (Figures S6H and S6I). The relative proportion of LRP neurons among SST+ neurons was also similar in both genotypes (Figures S6H and S6J).

To determine whether Dach1 expression is sufficient to change the relative proportion of LRP neurons among cortical SST+ cells, we infected $Sst^{Cre/+}$ embryos with conditional retroviruses expressing Cre-dependent control or *Dach1* vectors at E14.5 and examined the identity of SST+ neurons in the cortex of P10 mice (Figure S7A). We found that postmitotic Dach1

CellPress
OPEN ACCESS

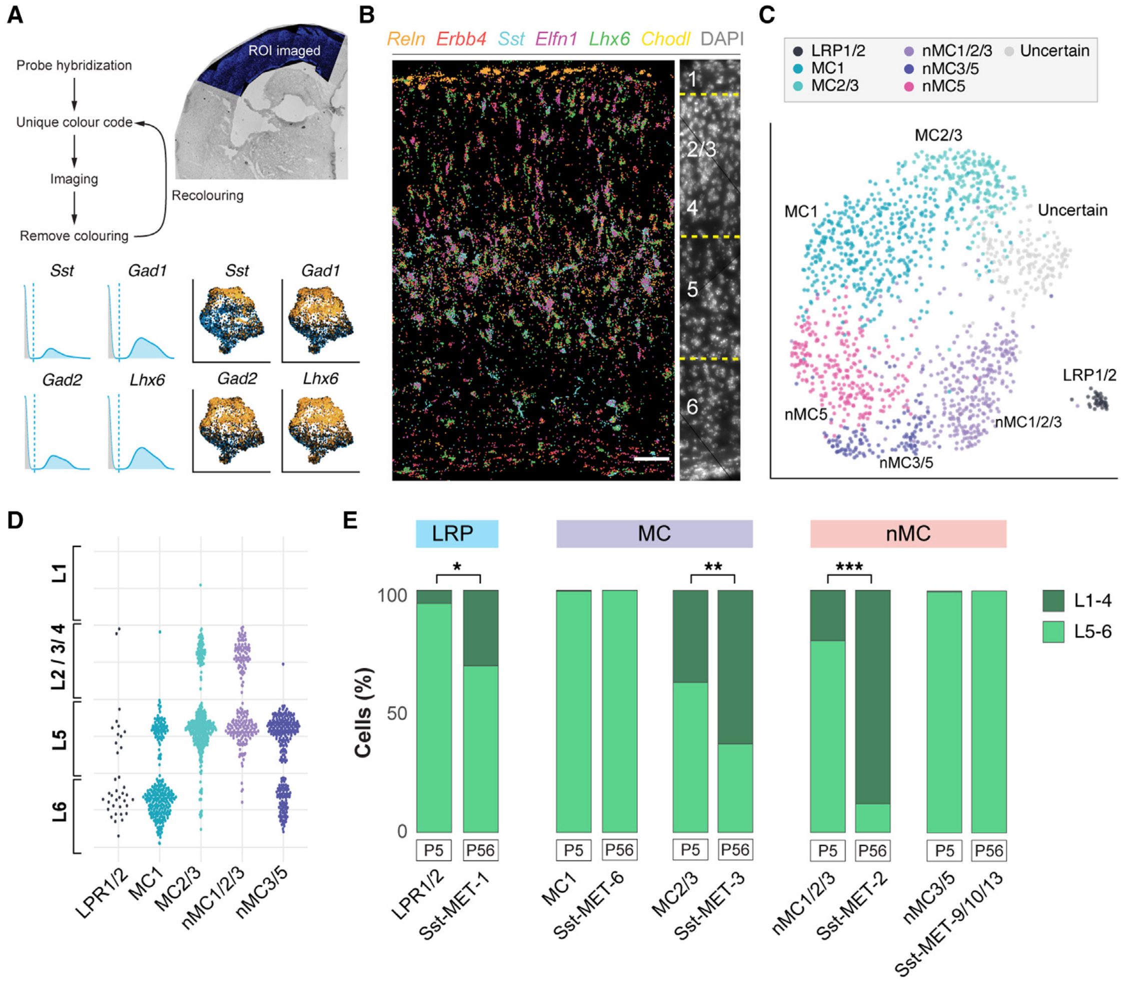

Figure 5. Spatial transcriptomics reveals the laminar distribution of SST+ subtypes at P5

(A) Schema illustrating molecular cartography of 94 genes (Table S4) using unique color barcoding (Resolve Biosciences). Each cell was segmented using the DAPI signal. Gene expression is visualized by UMAP. The bottom plots illustrate the expression profiles of *Sst*, *Gad1*, *Gad2*, and *Lhx6* among all neurons, which were used to filter SST+ neurons.

(B) Coronal section through the P5 mouse neocortex illustrating the expression of selected marker genes using molecular cartography.

(C) UMAP visualization of 2,319 SST+ positive neurons, clustered and annotated based on the strongest correlation to subtypes defined through scRNA-seq (Figure 1).

(D) Dot-plot diagram representing the laminar location of cells in each cluster.

(E) Comparison of the laminar distribution of each spatial cluster at P5 and its corresponding adult Sst-MET type. Fisher exact test: $^{*}p < 0.05$, $^{**}p < 0.01$, $^{***}p < 0.001$.

Scale bars, 100 μm.

overexpression in SST+ cells does not alter the proportion of LRP neurons among cortical SST+ cells (Figure S7B). Similar results were obtained in another series of experiments in which we infected the progenitor cells of the medial ganglionic eminence using the same retroviral strategy (Figures S7C and S7D). These experiments revealed that overexpression of Dach1 is insufficient to modify the identity of SST+ neurons.

We next explored whether Dach1 is required to develop axonal projections from SST+ LRP neurons because this is one of their most characteristic features. To this end, we combined genetic and viral-mediated anterograde tracing approaches to examine the axonal projection of LRP cells. In brief, we injected adeno-associated viruses (AAVs) coding Cre-dependent EGFP under an interneuron-specific promoter (*Dlx5/6*) into the primary motor cortex (M1) of P56 control and conditional *Dach1* mutant mice (Figure 6A). 2 weeks later, we confirmed that only SST+ cells were infected with AAVs (Figure 6B) and examined the pattern of striatal projections from SST+ LRP neurons. We found that SST+ LRP neurons preferentially project to the ipsilateral striatum in control mice (Figures 6C and 6D). By contrast, we observed that many axonal projections from cortical SST+ LRP neurons target the contralateral striatum in conditional *Dach1* mutant mice (Figures 6C and 6D). We performed separate experiments injecting AAVs at P15 to determine whether these defects

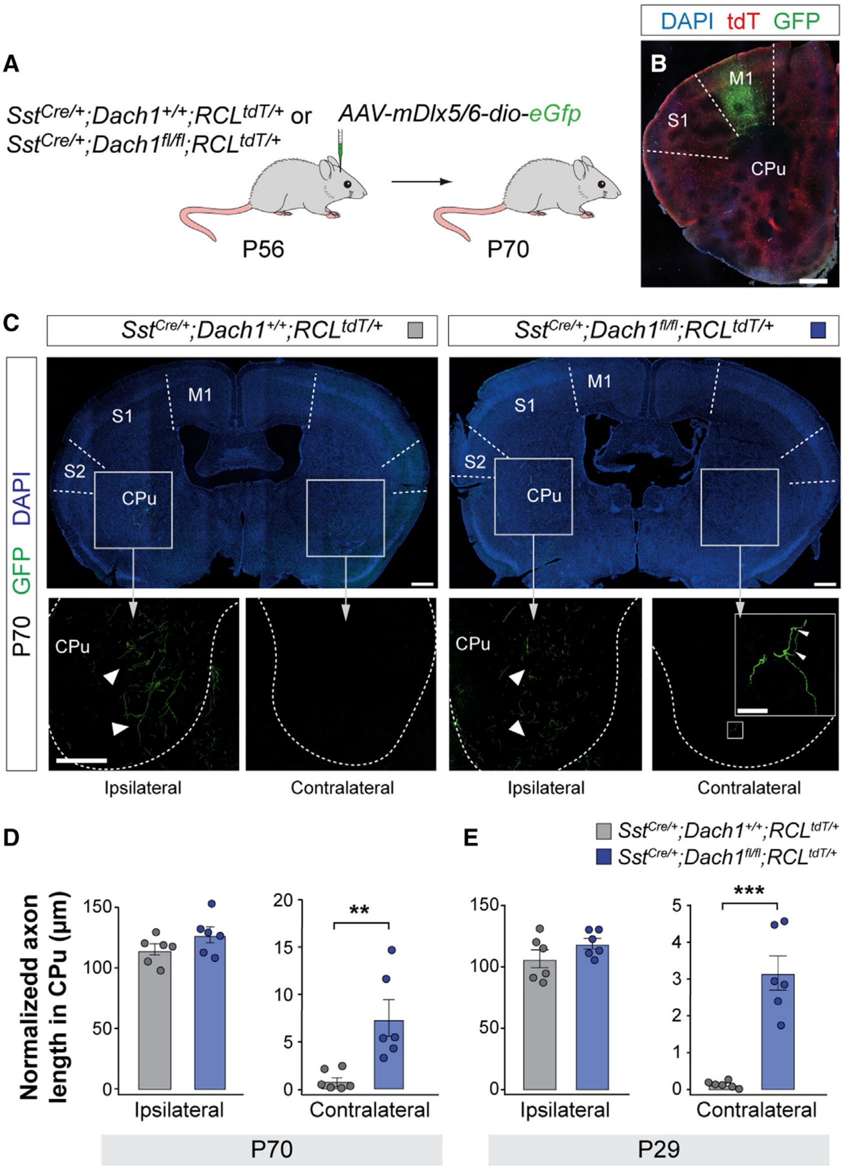

Figure 6. Conditional deletion of *Dach1* from SST+ neurons abnormally increases their contralateral axonal projections

(A) Schematic of the experimental design.

(B) Coronal section through the injection site stained with antibodies against tdTomato and GFP. DAPI staining reveals the distribution of nuclei.

(C) Coronal sections through the mouse telencephalon stained with antibodies against GFP in control and *Dach1* conditional mutants at P70. DAPI staining reveals the distribution of nuclei. The high-magnification images illustrate axons (arrowheads) in the striatum (CPu).

(D and E) Quantification of total axonal length in ipsilateral and contralateral striatum normalized by the number of infected cells in each animal (n = 6 mice per genotype) at P70 (D) and P29 (E). Two-tailed t test: $^{*}p < 0.05$.

Data are shown as mean ± SEM. Scale bars, 200 μm.

were already apparent at earlier stages of development and found a similar excess of contralateral projections 2 weeks later (Figure 6E). These results suggested that Dach1 regulates the development of axonal projections for a population of SST+ LRP neurons.

Next, we examined the role of Pou3f2 in developing SST+ LRP neurons. We first confirmed that Pou3f2 is expressed in SST+ LRP neurons by colocalization with NOS1 at P5 and P21. Consistent with our scRNA-seq analyses, we found that Pou3f2 is not expressed in SST+ interneurons (Figures S8A and S8B). We generated conditional mouse mutants in which *Pou3f2* is exclusively deleted from SST+ neurons (Figure S8C) and assessed the density of LRP neurons in the postnatal cortex. We found that the density of LRP neurons is similar between control and *Sst*$^{Cre/+}$;*Pou3f2*$^{fl/fl}$ mice at P5 (Figures 7A and 7B). By contrast, we found a significant reduction of LRP neurons across several cortical areas at P21, which was more prominent in the motor cortex (Figures 7A–7C). This reduction is already evident at P10 and extends progressively well beyond the normal period of programmed cell death for SST+ cells[34] (Figure 7D). We verified that the reduction in LRP neurons is simply not due to the loss of NOS1 expression because quantification of NPY+/SST+ cells, which also identify LRP neurons,[14] revealed a similar result (Figures S8D and S8E). We also examined whether Pou3f2 expression is sufficient to change the relative proportion of LRP neurons among cortical SST+ cells. To this end, we performed retroviral experiments similar to those described for Dach1. We found that overexpression of Pou3f2 in postmitotic SST+ cells or progenitor cells of the medial ganglionic eminence does not alter the proportion of LRP neurons among cortical SST+ cells (Figure S7). Altogether, these results suggest that Pou3f2 is insufficient to modify the identity of SST+ neurons but is required for the survival of SST+ LRP neurons.

To identify possible mediators of the function of Pou3f2 in the survival of SST+ LRP neurons, we explored known molecular targets of this transcription factor. We took advantage of a ChIP-seq dataset that identified over 2,000 potential gene targets of Pou3f2 in melanocytes.[35] Among those, we found 18 genes differentially expressed in adult LRP cells and enriched in these cells during development. Among these, we noticed that Sox2 was previously identified as part of the active node of transcription factors linked to Pou3f2 and Dach1 in the STRING network analysis (Figure S6A). Sox2 regulates cell survival or prevents apoptosis in multiple neuronal[36,37] and non-neuronal cell types, such as hair cells[38] and cancer cells.[39,40] Overexpression of *Dichaete*, the fly homolog of Sox2, is anti-apoptotic in neural

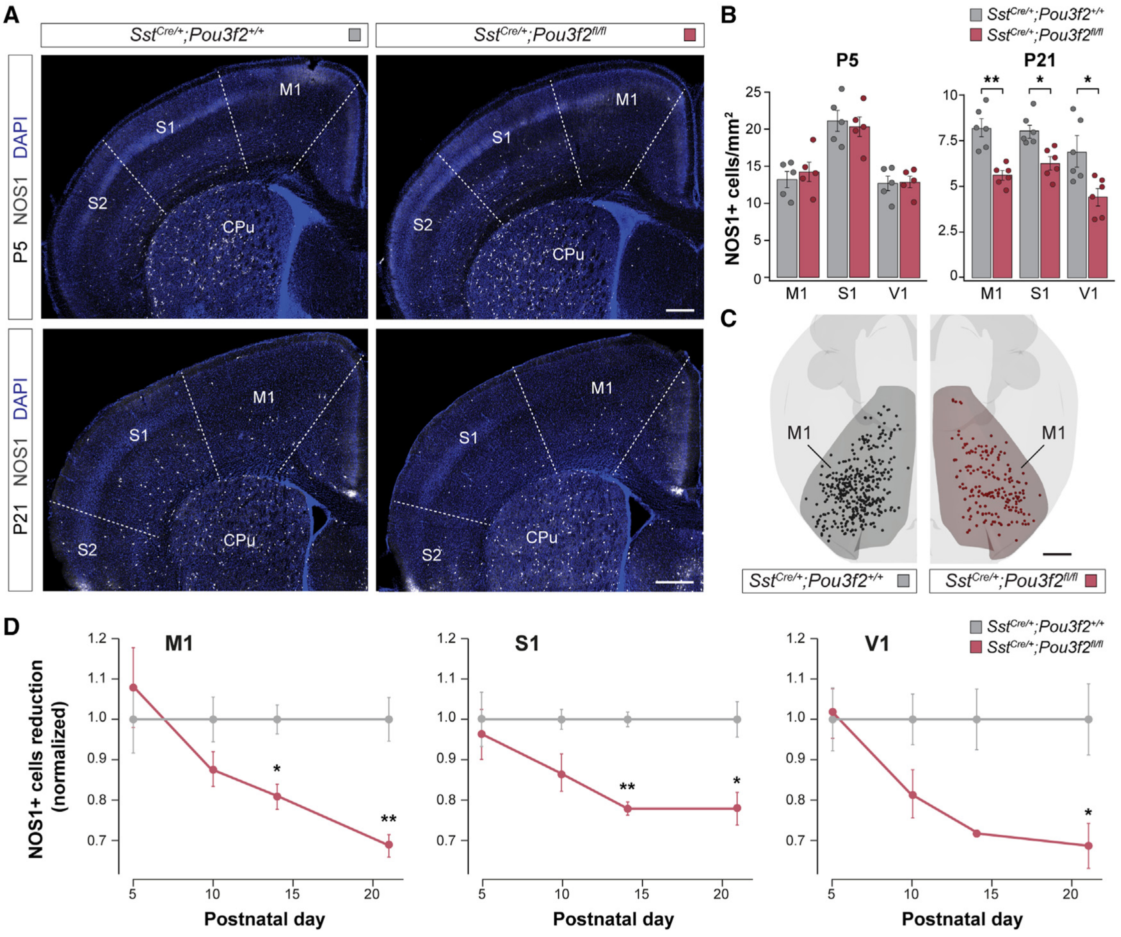

Figure 7. Conditional deletion of *Pou3f2* from SST+ neurons progressively reduces the density of LRP neurons

(A) Coronal sections through the mouse telencephalon stained with antibodies against NOS1 in control and *Pouf3f2* conditional mutants at P5 and P21. DAPI staining reveals the distribution of nuclei.

(B) Quantification of the density of NOS1+ cells in the motor cortex (M1), somatosensory cortex (S1), and visual cortex (V1) at P5 (n = 5 mice per genotype) and P21 (n = 6 mice per genotype). Student's t test with Bonferroni: $^{*}p < 0.05$, $^{**}p < 0.01$.

(C) Schematic representation of the spatial distribution of LRP neurons across the entire motor cortex in control and *Pouf3f2* conditional mutants P21.

(D) Normalized reduction of LRP neurons in M1, S1, and V1 from P5 to P21. Two-way ANOVA: $^{*}p < 0.05$, $^{**}p < 0.01$.

Data are shown as mean ± SEM. Scale bars, 500 μm.

stem cells, whereas loss of *Dichaete* function results in premature death.[41] In the brain, Sox2 expression has been shown in neuronal progenitors[42,43] and astrocytes[44] and is directly regulated by Pou3f2.[45] In the developing ganglionic eminences, Pou3f2 binds the promoter region of Sox2, and *Pou3f2*$^{-/-}$ monkey fetuses have reduced levels of Sox2.[45]

To determine whether Sox2 could mediate the function of Pou3f2 in the survival of LRP neurons, we first validated its expression in SST+ LRP cells. Consistent with our scRNA-seq data, we found that Sox2 is expressed at much higher levels in LRP neurons than in SST+ interneurons (Figures 8A and 8B). Next, we assessed Sox2 levels in SST+ LRP neurons in control and *Pou3f2* conditional mutant at P5 when the density of LRP neurons was still normal. Using immunohistochemistry, we found that Sox2 levels are significantly reduced in LRP neurons in the conditional *Pou3f2* mutants compared with controls (Figures 8C and 8D). We also isolated RNA from SST+ neurons obtained by FACS from control and conditional *Pou3f2* mutants and performed quantitative PCR (qPCR) analyses. Along with a prominent reduction in *Pou3f2* levels, we observed a prominent reduction in *Sox2* RNA in SST+ neurons from conditional *Pou3f2* mutants compared with controls (Figure 8E). Altogether, our experiments suggest that Pou3f2 is required for the expression of Sox2 in SST+ LRP cells and that the gradual loss of these neurons in *Pou3f2* conditional mutants may be at least in part mediated by this factor.

Finally, we explored whether Pou3f2 is required to develop axonal projections in SST+ LRP neurons. To this end, we performed similar AAV tracing experiments to those described for *Dach1* conditional mutants. We injected AAVs coding Cre-dependent mScarlet under an interneuron-specific promoter (*Dlx5/6*) into M1 of P29 control and conditional *Pou3f2* mutant mice (Figures S8F and S8G). Two weeks later, we found no differences

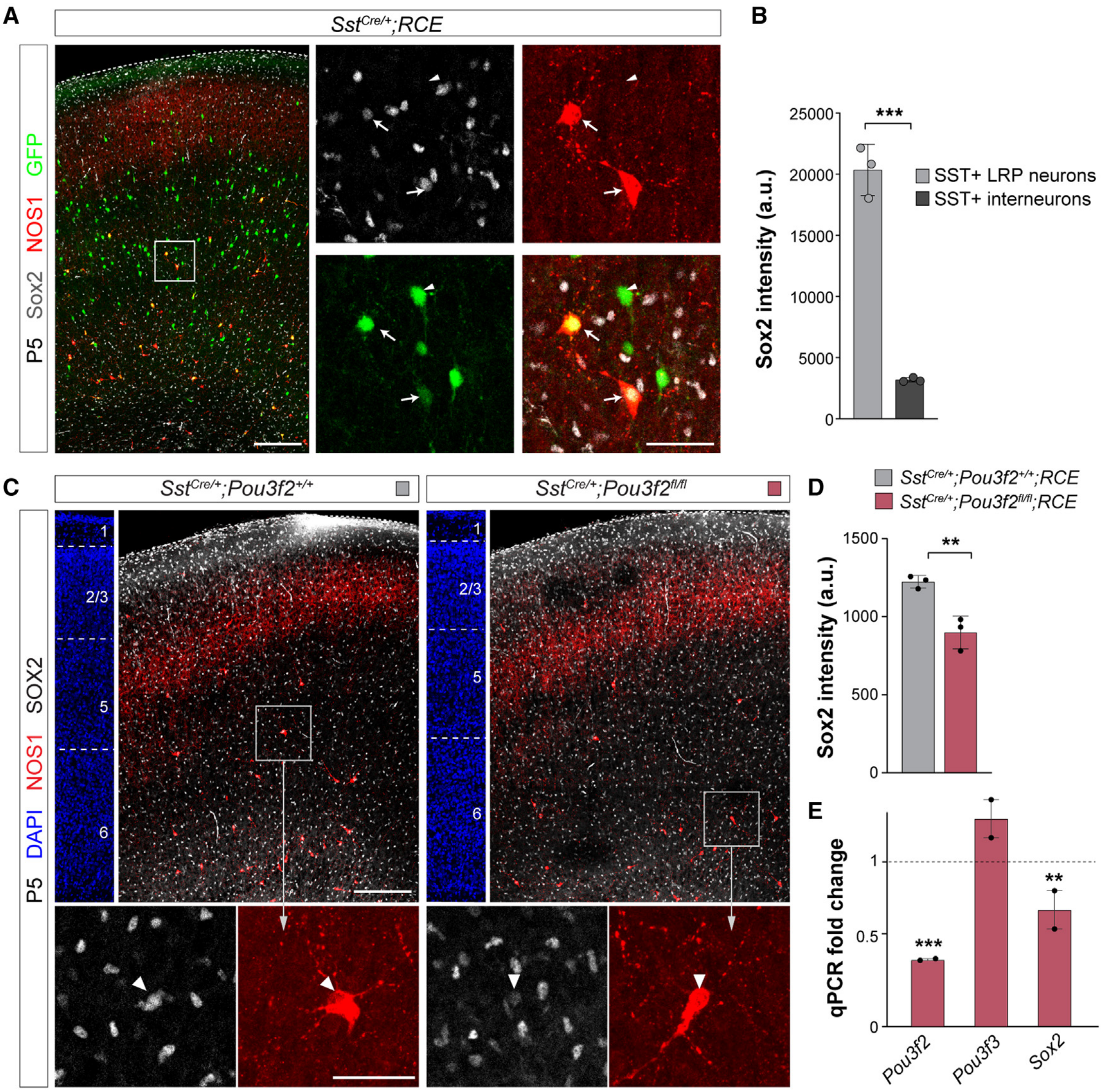

Figure 8. Sox2 levels are reduced in long-range cells in *Pou3f2* conditional mutant

(A) Coronal sections through the telencephalon of P5 *Sst*$^{Cre/+}$*;RCE* mice stained with antibodies against Sox2, NOS1, and GFP.

(B) Quantification of Sox2 intensity measurement in SST+ LRP neurons and SST+ interneurons. Student's t test: ***$p < 0.001$.

(C) Coronal sections through the mouse telencephalon stained with antibodies against Sox2 and NOS1 in control and *Pou3f2* conditional mutants at P5. DAPI staining reveals the distribution of nuclei. High-magnification images illustrate Sox2 intensity levels in NOS1+ neurons.

(D) Quantification of Sox2 intensity levels (n = 3 mice per genotype). Student's t test: **$p < 0.01$.

(E) Quantitative polymerase chain reaction analysis of *Pou3f2*, *Pou3f3*, and *Sox2* in SST+ neurons of *Pou3f2* conditional mutants and litter controls at P5. *Pou3f2* mutants were normalized to the average value of *Pou3f2* control animals. Pou3f3 was used as a control. Student's t test: **$p < 0.01$, ***$p < 0.001$.

Data are shown as mean ± SEM. Scale bars, 200 and 50 μm (inserts).

in the pattern of striatal projections or total length of axonal projections of SST+ LRP neurons in *Pou3f2* conditional mutants compared with control mice (Figures S8H and S8I). These experiments revealed that *Pou3f2* is not involved in the development of axonal projections for a population of SST+ LRP neurons.

DISCUSSION

The mammalian cerebral cortex contains an astonishing diversity of inhibitory neurons. Recent transcriptomic analyses suggest that cortical inhibitory neurons are organized according to a general principle of hierarchical relationship. According to this notion, the main subclasses of inhibitory neurons constitute major branches in a theoretical diversification tree, and each of these branches comprises progressively more similar neuronal populations up to the level of specific subtypes.[1] This hierarchical organization is thought to reflect the developmental origins of these cells.

A plethora of developmental studies have investigated the origin of inhibitory neurons in the mouse cerebral cortex and

CellPress
OPEN ACCESS

established that the different subclasses of inhibitory neurons derive from distinct progenitor pools in the ganglionic eminences and preoptic region of the subpallium.[18,46–53] Moreover, the main molecular programs controlling the specification of the different subclasses of neurons have also been discovered.[54–59] However, identifying the mechanisms through which diversification emerges within each of the main subclasses of cortical inhibitory neurons has been a significant challenge.

Two models have been proposed to explain the diversification of cortical GABAergic neurons.[12] One model suggests that specific transcriptional programs specify the different subtypes of GABAergic neurons during embryonic development, either at the progenitor level or shortly after neurogenesis. The alternative model proposes that GABAergic neurons are only specified embryonically into major subclasses. In this model, the emergence of distinct subtypes of cortical GABAergic neurons requires extrinsic factors that are only available in the postnatal brain, such as neuronal activity. Addressing this question requires unequivocally identifying distinct neuronal populations during development and mapping their developmental trajectory to specific subtypes of neurons in the adult brain.

Single-cell transcriptomics allows the development of a cross-stage taxonomy of cell types based on transcriptional similarity.[22,60,61] This approach is based on the idea that, although some molecular programs might be specific to certain developmental stages (e.g., those regulating cell migration or axon guidance), the critical factors involved in the specification of neuronal subtypes are also required to maintain their terminally differentiated state in the adult brain.[62] Our experiments indicate that at least some subtypes of cortical SST+ neurons are fated during embryonic stages because many developing SST+ cells unfold highly specific molecular programs directly related to those expressed by certain subtypes of cortical SST+ neurons in the adult cortex. For example, we identified two distinct subtypes of SST+ LRP neurons in the embryonic cortex that match their counterparts in the adult. A similar observation has been made for chandelier cells, a subtype of PV+ interneurons for which there is clear evidence of early fate commitment during development.[63]

Our analysis also suggests that the diversification of SST+ interneurons follows a more complex dynamic. Although some populations of embryonic and early postnatal SST+ interneurons align well with a specific adult subtype (e.g., MC1 seems to correspond to deep-layer alpha-2 T-shaped MCs), most groups of SST+ cells identified during development exhibit transcriptional similarity to several highly related subtypes of interneurons in the adult cortex. This is particularly prominent for non-Martinotti subtypes, which can be transcriptionally linked to more than one developmental cell population. These observations suggest that the diversification of at least some subtypes of SST+ interneurons may require other signals than those intrinsically programmed during embryonic development. Alternatively, it is possible that multiple nMCs map to a single adult type because they correspond to interneurons born at different times. If this were true, it would also imply that, compared with MCs and LRP neurons, nMCs are generated throughout an extended period of neurogenesis. Unfortunately, our current analysis lacks the resolution to resolve different neurogenic cohorts and cannot distinguish between both possibilities. Additional experiments involving labeling isochronic cell populations *in vivo*,[64] with increased sequencing depth, may help resolve this issue. Using cells from species with high neoteny—such as human interneurons—would also increase the temporal resolution required to address this question.

The differentiation of neuronal subtypes is often controlled by a small subset of transcription factors.[65,66] Here, we have begun to unravel the molecular program driving the development of SST+ LRP neurons, a relatively rare subtype of GABAergic neuron among the most conserved cell types in the mammalian NCx.[1] Similar to other cortical SST+ neurons, LRP neurons likely derive from the dorsal region of the medial ganglionic eminence,[46,67,68] but the factors regulating their development have remained elusive. Two transcription factors, Dach1 and Pou3f2, emerge as important regulators of the development of SST+ LRP neurons.

The transcription factor Dach1 is a mouse homolog of *dachshund*, a gene involved in eye, leg, and brain development in *Drosophila*.[69–71] Dach1 is expressed in progenitor cells throughout the telencephalon in mouse and human embryos.[72,73] Homozygous *Dach1* mutant mice die shortly after birth without gross brain abnormalities.[74,75] Our study reveals that Dach1 is required for the normal development of the long-range axonal projections that characterize SST+ LRP neurons. This observation suggests this transcription factor might play similar roles for other neuronal populations in the developing telencephalon.

Pou3f2/Brn2 belongs to the Pit-Oct-Unc (POU) family transcription factors, which play critical roles in cell-fate determination and maintenance in the nervous system.[76–79] In the mouse NCx, Pou3f2 is expressed by pallial progenitor cells and newborn excitatory neurons and, together with Pou3f3/Brn1, is required for the fate specification and migration of superficial layer excitatory neurons.[80–82] Recent work suggests that Pou3f2 regulates the patterning of the ganglionic eminences in primates.[45] In humans, loss-of-function mutations in *Pou3f2* cause neurodevelopmental delay, intellectual disability, and obesity.[83] Despite the central role of Pou3f2 in brain development, its function in the development of GABAergic LRP neurons has not been explored. We found that Pou3f2 controls the survival of SST+ LRP neurons postnatally. Loss of Pou3f2 leads to a prominent reduction of Sox2 levels, a transcription factor that plays crucial roles in cell survival in various cell types.[36–40] Additional experiments will be needed to identify the precise function of Pou3f2 and Sox2 in the survival of SST+ LRP neurons.

STAR★METHODS

Detailed methods are provided in the online version of this paper and include the following:

- KEY RESOURCES TABLE
- RESOURCE AVAILABILITY
  - Lead contact
  - Materials availability
  - Data and code availability

- EXPERIMENTAL MODEL AND STUDY PARTICIPANT DETAILS
  - Mice
- METHOD DETAILS
  - FACS experiments
  - Bioinformatic analyses
  - AAV experiments
  - Retrovirus experiments
  - Histology and immunohistochemistry
  - Quantitative polymerase chain reaction
- QUANTIFICATION AND STATISTICAL ANALYSIS
  - Cell density
  - Axonal length
  - Sox2 levels
  - Dach1 and Pou3f2 overexpression experiments

SUPPLEMENTAL INFORMATION

Supplemental information can be found online at https://doi.org/10.1016/j.neuron.2023.11.013.

ACKNOWLEDGMENTS

We thank E. Serafeimidou-Pouliou for general laboratory support, C. Zimmer for help with retroviral experiments, I. Andrew for managing mouse colonies, and the Flow Cytometry and Genomics Platforms at the Guy's and St Thomas' NHS Foundation Trust and King's College London Biomedical Research Centre for technical support. We also thank the VIB Flow Core Leuven and VIB-CBD Single Cell & Microfluidics Units. We thank Genevia Technologies for their help with statistical analyses. We are also grateful to members of the Lim, Marín, Mi, and Rico laboratories for stimulating discussions and ideas and to Nicholas Luscombe for guidance and support on bioinformatic analyses. This work was supported by grants from the Wellcome Trust (215556/Z/19/Z) to O.M., the Research Foundation – Flanders (FWO) Junior Research Project Fundamental Research (G057121N), the FWO-Odysseus Award (G0E9121N), and the VIB Technology Watch (Techwatch) grant (Spatial technologies) to L.L., and the Ministry of Science and Technology of China (STI2030-Major Projects 2021ZD0202300) and the National Natural Science Foundation of China (32271024) to D.M. E.M. was the recipient of an FWO PhD fundamental fellowship (1192822N).

AUTHOR CONTRIBUTIONS

Conceptualization, L.L., O.M., and D.M.; methodology, J.F., M.V., L.L., D.M., and O.M.; investigation, M.V., Z.L., M.M., R.M., E.M., C.M.A., L.L., and D.M.; data curation, J.F., C.H., Y.Y., - J.W., L.L., and D.M.; formal analysis, J.F., C.H., Y.Y., and J.W.; writing – original draft, D.M., O.M., and L.L.; writing – review & editing, M.V., J.F., M.M., Z.L., D.M., O.M., and L.L.; funding acquisition, L.L., O.M., and D.M.; resources, L.L., O.M., and D.M.; supervision, L.L., M.V., O.M., and D.M.

DECLARATION OF INTERESTS

The authors declare no competing interests.

INCLUSION AND DIVERSITY

We support inclusive, diverse, and equitable conduct of research. One or more of the authors of this paper received support from a program designed to increase minority representation in their field of research.

Received: November 7, 2022
Revised: August 22, 2023
Accepted: November 10, 2023
Published: February 21, 2024

REFERENCES

1. BRAIN Initiative Cell Census Network (BICCN) (2021). A multimodal cell census and atlas of the mammalian primary motor cortex. Nature *598*, 86–102.
2. Gouwens, N.W., Sorensen, S.A., Baftizadeh, F., Budzillo, A., Lee, B.R., Jarsky, T., Alfiler, L., Baker, K., Barkan, E., Berry, K., et al. (2020). Integrated morphoelectric and transcriptomic classification of cortical GABAergic cells. Cell *183*, 935–953.e19.
3. Klausberger, T., and Somogyi, P. (2008). Neuronal diversity and temporal dynamics: the unity of hippocampal circuit operations. Science *321*, 53–57.
4. Mayer, C., Hafemeister, C., Bandler, R.C., Machold, R., Batista-Brito, R., Jaglin, X., Allaway, K., Butler, A., Fishell, G., and Satija, R. (2018). Developmental diversification of cortical inhibitory interneurons. Nature *505*, 1–25.
5. Shi, Y., Wang, M., Mi, D., Lu, T., Wang, B., Dong, H., Zhong, S., Chen, Y., Sun, L., Zhou, X., et al. (2021). Mouse and human share conserved transcriptional programs for interneuron development. Science *374*, eabj6641.
6. Mi, D., Li, Z., Lim, L., Li, M., Moissidis, M., Yang, Y., Gao, T., Hu, T.X., Pratt, T., Price, D.J., et al. (2018). Early emergence of cortical interneuron diversity in the mouse embryo. Science *360*, 81–85.
7. Yu, Y., Zeng, Z., Xie, D., Chen, R., Sha, Y., Huang, S., Cai, W., Chen, W., Li, W., Ke, R., et al. (2021). Interneuron origin and molecular diversity in the human fetal brain. Nat. Neurosci. *24*, 1745–1756.
8. Zhao, Z., Zhang, D., Yang, F., Xu, M., Zhao, S., Pan, T., Liu, C., Liu, Y., Wu, Q., Tu, Q., et al. (2022). Evolutionarily conservative and non-conservative regulatory networks during primate interneuron development revealed by single-cell RNA and ATAC sequencing. Cell Res. *32*, 425–436.
9. Cheng, S., Butrus, S., Tan, L., Xu, R., Sagireddy, S., Trachtenberg, J.T., Shekhar, K., and Zipursky, S.L. (2022). Vision-dependent specification of cell types and function in the developing cortex. Cell *185*, 311–327.e24.
10. De Marco García, N.V.D.M., Karayannis, T., and Fishell, G. (2011). Neuronal activity is required for the development of specific cortical interneuron subtypes. Nature *472*, 351–355.
11. De Marco García, N.V.D.M., Priya, R., Tuncdemir, S.N., Fishell, G., and Karayannis, T. (2015). Sensory inputs control the integration of neurogliaform interneurons into cortical circuits. Nat. Neurosci. *18*, 393–401.
12. Wamsley, B., and Fishell, G. (2017). Genetic and activity-dependent mechanisms underlying interneuron diversity. Nat. Rev. Neurosci. *18*, 299–309.
13. Tremblay, R., Lee, S., and Rudy, B. (2016). GABAergic interneurons in the neocortex: from cellular properties to circuits. Neuron *91*, 260–292.
14. Tomioka, R., Okamoto, K., Furuta, T., Fujiyama, F., Iwasato, T., Yanagawa, Y., Obata, K., Kaneko, T., and Tamamaki, N. (2005). Demonstration of long-range GABAergic connections distributed throughout the mouse neocortex. Eur. J. Neurosci. *21*, 1587–1600.
15. He, M., Tucciarone, J., Lee, S., Nigro, M.J., Kim, Y., Levine, J.M., Kelly, S.M., Krugikov, I., Wu, P., Chen, Y., et al. (2016). Strategies and tools for combinatorial targeting of GABAergic neurons in mouse cerebral cortex. Neuron *91*, 1228–1243.
16. Gerashchenko, D., Wisor, J.P., Burns, D., Reh, R.K., Shiromani, P.J., Sakurai, T., de la Iglesia, H.O., and Kilduff, T.S. (2008). Identification of a population of sleep-active cerebral cortex neurons. Proc. Natl. Acad. Sci. USA *105*, 10227–10232.
17. Zielinski, M.R., Atochin, D.N., McNally, J.M., McKenna, J.T., Huang, P.L., Strecker, R.E., and Gerashchenko, D. (2019). Somatostatin+/nNOS+ neurons are involved in delta electroencephalogram activity and cortical-dependent recognition memory. Sleep *42*, zsz143.
18. Lim, L., Mi, D., Llorca, A., and Marín, O. (2018). Development and functional diversification of cortical interneurons. Neuron *100*, 294–313.

19. Lim, L., Pakan, J.M.P., Selten, M.M., Marques-Smith, A., Llorca, A., Bae, S.E., Rochefort, N.L., and Marín, O. (2018). Optimization of interneuron function by direct coupling of cell migration and axonal targeting. Nat. Neurosci. *21*, 920–931.
20. Tasic, B., Yao, Z., Graybuck, L.T., Smith, K.A., Nguyen, T.N., Bertagnolli, D., Goldy, J., Garren, E., Economo, M.N., Viswanathan, S., et al. (2018). Shared and distinct transcriptomic cell types across neocortical areas. Nature *563*, 72–78.
21. Frazer, S., Prados, J., Niquille, M., Cadilhac, C., Markopoulos, F., Gomez, L., Tomasello, U., Telley, L., Holtmaat, A., Jabaudon, D., et al. (2017). Transcriptomic and anatomic parcellation of 5-HT3AR expressing cortical interneuron subtypes revealed by single-cell RNA sequencing. Nat. Commun. *8*, 14219.
22. Crow, M., Paul, A., Ballouz, S., Huang, Z.J., and Gillis, J. (2018). Characterizing the replicability of cell types defined by single cell RNA-sequencing data using MetaNeighbor. Nat. Commun. *9*, 884.
23. Hilscher, M.M., Leão, R.N., Edwards, S.J., Leão, K.E., and Kullander, K. (2017). Chrna2-Martinotti cells synchronize Layer 5 Type A pyramidal cells via rebound excitation. PLoS Biol. *15*, e2001392.
24. Muñoz, W., Tremblay, R., Levenstein, D., and Rudy, B. (2017). Layer-specific modulation of neocortical dendritic inhibition during active wakefulness. Science *355*, 954–959.
25. Nigro, M.J., Hashikawa-Yamasaki, Y., and Rudy, B. (2018). Diversity and connectivity of Layer 5 somatostatin-expressing interneurons in the mouse barrel cortex. J. Neurosci. *38*, 1622–1633.
26. Ma, Y., Hu, H., Berrebi, A.S., Mathers, P.H., and Agmon, A. (2006). Distinct subtypes of somatostatin-containing neocortical interneurons revealed in transgenic mice. J. Neurosci. *26*, 5069–5082.
27. Scala, F., Kobak, D., Shan, S., Bernaerts, Y., Laturnus, S., Cadwell, C.R., Hartmanis, L., Froudarakis, E., Castro, J.R., Tan, Z.H., et al. (2019). Layer 4 of mouse neocortex differs in cell types and circuit organization between sensory areas. Nat. Commun. *10*, 4174.
28. Xu, H., Jeong, H.-Y., Tremblay, R., and Rudy, B. (2013). Neocortical somatostatin-expressing GABAergic interneurons disinhibit the thalamorecipient layer 4. Neuron *77*, 155–167.
29. Yao, Z., van Velthoven, C.T.J., Nguyen, T.N., Goldy, J., Sedeno-Cortes, A.E., Baftizadeh, F., Bertagnolli, D., Casper, T., Chiang, M., Crichton, K., et al. (2021). A taxonomy of transcriptomic cell types across the isocortex and hippocampal formation. Cell *184*, 3222–3241.e26.
30. Telley, L., Agirman, G., Prados, J., Amberg, N., Fièvre, S., Oberst, P., Bartolini, G., Vitali, I., Cadilhac, C., Hippenmeyer, S., et al. (2019). Temporal patterning of apical progenitors and their daughter neurons in the developing neocortex. Science *364*, eaav2522–eaav2529.
31. Szklarczyk, D., Franceschini, A., Wyder, S., Forslund, K., Heller, D., Huerta-Cepas, J., Simonovic, M., Roth, A., Santos, A., Tsafou, K.P., et al. (2015). STRING v10: protein–protein interaction networks, integrated over the tree of life. Nucleic Acids Res. *43*, D447–D452.
32. Magno, L., Oliveira, M.G., Mucha, M., Rubin, A.N., and Kessaris, N. (2012). Multiple embryonic origins of nitric oxide synthase-expressing GABAergic neurons of the neocortex. Front. Neural Circuits *6*, 65.
33. Perrenoud, Q., Geoffroy, H., Gauthier, B., Rancillac, A., Alfonsi, F., Kessaris, N., Rossier, J., Vitalis, T., and Gallopin, T. (2012). Characterization of type I and Type II nNOS-expressing interneurons in the barrel cortex of mouse. Front. Neural Circuits *6*, 36.
34. Wong, F.K., Bercsenyi, K., Sreenivasan, V., Portalés, A., Fernández-Otero, M., and Marín, O. (2018). Pyramidal cell regulation of interneuron survival sculpts cortical networks. Nature *557*, 668–673.
35. Kobi, D., Steunou, A.L., Dembélé, D., Legras, S., Larue, L., Nieto, L., and Davidson, I. (2010). Genome-wide analysis of POU3F2/BRN2 promoter occupancy in human melanoma cells reveals Kitl as a novel regulated target gene. Pigment Cell Melanoma Res. *23*, 404–418.
36. Taranova, O.V., Magness, S.T., Fagan, B.M., Wu, Y., Surzenko, N., Hutton, S.R., and Pevny, L.H. (2006). SOX2 is a dose-dependent regulator of retinal neural progenitor competence. Genes Dev. *20*, 1187–1202.
37. Graham, V., Khudyakov, J., Ellis, P., and Pevny, L. (2003). SOX2 functions to maintain neural progenitor identity. Neuron *39*, 749–765.
38. Chen, D., Jia, G., Zhang, Y., Mao, H., Zhao, L., Li, W., Chen, Y., and Ni, Y. (2022). Sox2 overexpression alleviates noise-induced hearing loss by inhibiting inflammation-related hair cell apoptosis. J. Neuroinflammation *19*, 59.
39. Chen, S., Li, X., Lu, D., Xu, Y., Mou, W., Wang, L., Chen, Y., Liu, Y., Li, X., Li, L.-Y., et al. (2014). SOX2 regulates apoptosis through MAP4K4-survivin signaling pathway in human lung cancer cells. Carcinogenesis *35*, 613–623.
40. Yang, N., Wang, Y., Hui, L., Li, X., and Jiang, X. (2015). Silencing SOX2 expression by RNA interference inhibits proliferation, invasion and metastasis, and induces apoptosis through MAP4K4/JNK signaling pathway in human laryngeal cancer TU212 cells. J. Histochem. Cytochem. *63*, 721–733.
41. Harding, K., Heath, K., and White, K. (2021). Dichaete, a Sox2 homologue, prevents activation of cell death in multiple developmental contexts.
42. Favaro, R., Valotta, M., Ferri, A.L.M., Latorre, E., Mariani, J., Giachino, C., Lancini, C., Tosetti, V., Ottolenghi, S., Taylor, V., et al. (2009). Hippocampal development and neural stem cell maintenance require Sox2-dependent regulation of Shh. Nat. Neurosci. *12*, 1248–1256.
43. Ferri, A., Favaro, R., Beccari, L., Bertolini, J., Mercurio, S., Nieto-Lopez, F., Verzeroli, C., La Regina, F., De Pietri Tonelli, D., Ottolenghi, S., et al. (2013). Sox2 is required for embryonic development of the ventral telencephalon through the activation of the ventral determinants Nkx2.1 and Shh. Development *140*, 1250–1261.
44. Wang, Y., Zhang, S., Lan, Z., Doan, V., Kim, B., Liu, S., Zhu, M., Hull, V.L., Rihani, S., Zhang, C.-L., et al. (2022). SOX2 is essential for astrocyte maturation and its deletion leads to hyperactive behavior in mice. Cell Rep. *41*, 111842.
45. Zhu, X., Guo, Y., Chu, C., Liu, D., Duan, K., Yin, Y., Si, C., Kang, Y., Yao, J., Du, X., et al. (2022). BRN2 as a key gene drives the early primate telencephalon development. Sci. Adv. *8*, eabl7263.
46. Flames, N., Pla, R., Gelman, D.M., Rubenstein, J.L.R., Puelles, L., and Marín, O. (2007). Delineation of multiple subpallial progenitor domains by the combinatorial expression of transcriptional codes. J. Neurosci. *27*, 9682–9695.
47. Nery, S., Fishell, G., and Corbin, J.G. (2002). The caudal ganglionic eminence is a source of distinct cortical and subcortical cell populations. Nat. Neurosci. *5*, 1279–1287.
48. Gelman, D.M., Martini, F.J., Nóbrega-Pereira, S., Pierani, A., Kessaris, N., and Marín, O. (2009). The embryonic preoptic area is a novel source of cortical GABAergic interneurons. J. Neurosci. *29*, 9380–9389.
49. Wichterle, H., Turnbull, D.H., Nery, S., Fishell, G., and Alvarez-Buylla, A. (2001). In utero fate mapping reveals distinct migratory pathways and fates of neurons born in the mammalian basal forebrain. Development *128*, 3759–3771.
50. Xu, Q., Cobos, I., de la Cruz, E., Rubenstein, J.L.R., and Anderson, S.A. (2004). Origins of cortical interneuron subtypes. J. Neurosci. *24*, 2612–2622.
51. Niquille, M., Limoni, G., Markopoulos, F., Cadilhac, C., Prados, J., Holtmaat, A., and Dayer, A. (2018). Neurogliaform cortical interneurons derive from cells in the preoptic area. eLife *7*, 1542.
52. Lavdas, A.A., Grigoriou, M., Pachnis, V., and Parnavelas, J.G. (1999). The medial ganglionic eminence gives rise to a population of early neurons in the developing cerebral cortex. J. Neurosci. *19*, 7881–7888.
53. Anderson, S.A., Eisenstat, D.D., Shi, L., and Rubenstein, J.L.R. (1997). Interneuron migration from basal forebrain to neocortex: dependence on Dlx genes. Science *278*, 474–476.

54. Sussel, L., Marin, O., Kimura, S., and Rubenstein, J.L.R. (1999). Loss of Nkx2.1 homeobox gene function results in a ventral to dorsal molecular respecification within the basal telencephalon: evidence for a transformation of the pallidum into the striatum. Development *126*, 3359–3370.

55. Butt, S.J.B., Sousa, V.H., Fuccillo, M.V., Hjerling-Leffler, J., Miyoshi, G., Kimura, S., and Fishell, G. (2008). The requirement of Nkx2-1 in the temporal specification of cortical interneuron subtypes. Neuron *59*, 722–732.

56. Kanatani, S., Yozu, M., Tabata, H., and Nakajima, K. (2008). COUP-TFII is preferentially expressed in the caudal ganglionic eminence and is involved in the caudal migratory stream. J. Neurosci. *28*, 13582–13591.

57. Tripodi, M., Filosa, A., Armentano, M., and Studer, M. (2004). The COUP-TF nuclear receptors regulate cell migration in the mammalian basal forebrain. Development *131*, 6119–6129.

58. Liodis, P., Denaxa, M., Grigoriou, M., Akufo-Addo, C., Yanagawa, Y., and Pachnis, V. (2007). Lhx6 activity is required for the normal migration and specification of cortical interneuron subtypes. J. Neurosci. *27*, 3078–3089.

59. Hu, J.S., Vogt, D., Sandberg, M., and Rubenstein, J.L.R. (2017). Cortical interneuron development: a tale of time and space. Development *144*, 3867–3878.

60. Welch, J.D., Kozareva, V., Ferreira, A., Vanderburg, C., Martin, C., and Macosko, E.Z. (2019). Single-cell multi-omic integration compares and contrasts features of brain cell identity. Cell *177*, 1873–1887.e17.

61. Stuart, T., Butler, A., Hoffman, P., Hafemeister, C., Papalexi, E., III, W.M.M., Hao, Y., Stoeckius, M., Smibert, P., and Satija, R. (2019). Comprehensive integration of single-cell data. Cell *177*, 1888–1902.e21.

62. Hobert, O., and Kratsios, P. (2019). Neuronal identity control by terminal selectors in worms, flies, and chordates. Curr. Opin. Neurobiol. *56*, 97–105.

63. Taniguchi, H., Lu, J., and Huang, Z.J. (2013). The spatial and temporal origin of chandelier cells in mouse neocortex. Science *339*, 70–74.

64. Govindan, S., Oberst, P., and Jabaudon, D. (2018). In vivo pulse labeling of isochronic cohorts of cells in the central nervous system using FlashTag. Nat. Protoc. *13*, 2297–2311.

65. Hobert, O. (2021). Homeobox genes and the specification of neuronal identity. Nat. Rev. Neurosci. *22*, 627–636.

66. Holguera, I., and Desplan, C. (2018). Neuronal specification in space and time. Science *362*, 176–180.

67. Fogarty, M., Grist, M., Gelman, D., Marín, O., Pachnis, V., and Kessaris, N. (2007). Spatial genetic patterning of the embryonic neuroepithelium generates GABAergic interneuron diversity in the adult cortex. J. Neurosci. *27*, 10935–10946.

68. Xu, Q., Tam, M., and Anderson, S.A. (2008). Fate mapping Nkx2.1-lineage cells in the mouse telencephalon. J. Comp. Neurol. *506*, 16–29.

69. Mardon, G., Solomon, N.M., and Rubin, G.M. (1994). dachshund encodes a nuclear protein required for normal eye and leg development in Drosophila. Development *120*, 3473–3486.

70. Kurusu, M., Nagao, T., Walldorf, U., Flister, S., Gehring, W.J., and Furukubo-Tokunaga, K. (2000). Genetic control of development of the mushroom bodies, the associative learning centers in the Drosophila brain, by the eyeless, twin of eyeless, and dachshund genes. Proc. Natl. Acad. Sci. USA *97*, 2140–2144.

71. Martini, S.R., Roman, G., Meuser, S., Mardon, G., and Davis, R.L. (2000). The retinal determination gene, dachshund, is required for mushroom body cell differentiation. Development *127*, 2663–2672.

72. Castiglioni, V., Faedo, A., Onorati, M., Bocchi, V.D., Li., Z., Iennaco, R., Vuono, R., Bulfamante, G.P., Muzio, L., Martino, G., et al. (2018). Dynamic and cell-specific DACH1 expression in human neocortical and striatal development. Cereb. Cortex *29*, 2115–2124.

73. Machon, O., van den Bout, C.J., Backman, M., Røsok, Ø., Caubit, X., Fromm, S.H., Geronimo, B., and Krauss, S. (2002). Forebrain-specific promoter/enhancer D6 derived from the mouse Dach1 gene controls expression in neural stem cells. Neuroscience *112*, 951–966.

74. Backman, M., Machon, O., Bout, C.J.V.D., and Krauss, S. (2003). Targeted disruption of mouse Dach1 results in postnatal lethality. Dev. Dyn. *226*, 139–144.

75. Davis, R.J., Shen, W., Sandler, Y.I., Amoui, M., Purcell, P., Maas, R., Ou, C.-N., Vogel, H., Beaudet, A.L., and Mardon, G. (2001). Dach1 mutant mice bear no gross abnormalities in eye, limb, and brain development and exhibit postnatal lethality. Mol. Cell. Biol. *21*, 1484–1490.

76. He, X., Treacy, M.N., Simmons, D.M., Ingraham, H.A., Swanson, L.W., and Rosenfeld, M.G. (1989). Expression of a large family of POU-domain regulatory genes in mammalian brain development. Nature *340*, 35–41.

77. Malik, V., Zimmer, D., and Jauch, R. (2018). Diversity among POU transcription factors in chromatin recognition and cell fate reprogramming. Cell. Mol. Life Sci. *75*, 1587–1612.

78. Serrano-Saiz, E., Leyva-Díaz, E., Cruz, E.D.L., and Hobert, O. (2018). BRN3-type POU homeobox genes maintain the identity of mature postmitotic neurons in nematodes and mice. Curr. Biol. *28*, 2813–2823.e2.

79. Jaegle, M., Ghazvini, M., Mandemakers, W., Piirsoo, M., Driegen, S., Levavasseur, F., Raghoenath, S., Grosveld, F., and Meijer, D. (2003). The POU proteins Brn-2 and Oct-6 share important functions in Schwann cell development. Genes Dev. *17*, 1380–1391.

80. Dominguez, M.H., Ayoub, A.E., and Rakic, P. (2013). POU-III transcription factors (Brn1, Brn2, and Oct6) influence neurogenesis, molecular identity, and migratory destination of upper-layer cells of the cerebral cortex. Cereb. Cortex *23*, 2632–2643.

81. McEvilly, R.J., de Diaz, M.O., Schonemann, M.D., Hooshmand, F., and Rosenfeld, M.G. (2002). Transcriptional regulation of cortical neuron migration by POU domain factors. Science *295*, 1528–1532.

82. Oishi, K., Aramaki, M., and Nakajima, K. (2016). Mutually repressive interaction between Brn1/2 and Rorb contributes to the establishment of neocortical layer 2/3 and layer 4. Proc. Natl. Acad. Sci. USA *113*, 3371–3376.

83. Schönauer, R., Jin, W., Findeisen, C., Valenzuela, I., Devlin, L.A., Murrell, J., Bedoukian, E.C., Pöschla, L., Hantmann, E., Riedhammer, K.M., et al. (2023). Monoallelic intragenic POU3F2 variants lead to neurodevelopmental delay and hyperphagic obesity, confirming the gene's candidacy in 6q16.1 deletions. Am. J. Hum. Genet. *110*, 998–1007.

84. Taniguchi, H., He, M., Wu, P., Kim, S., Paik, R., Sugino, K., Kvitsiani, D., Fu, Y., Lu, J., Lin, Y., et al. (2011). A resource of Cre driver lines for genetic targeting of GABAergic neurons in cerebral cortex. Neuron *71*, 995–1013.

85. Sousa, V.H., Miyoshi, G., Hjerling-Leffler, J., Karayannis, T., and Fishell, G. (2009). Characterization of Nkx6-2-derived neocortical interneuron lineages. Cereb. Cortex *19*, i1–i10.

86. Madisen, L., Garner, A.R., Shimaoka, D., Chuong, A.S., Klapoetke, N.C., Li, L., van der Bourg, A., Niino, Y., Egolf, L., Monetti, C., et al. (2015). Transgenic mice for intersectional targeting of neural sensors and effectors with high specificity and performance. Neuron *85*, 942–958.

87. Stewart, S.A., Dykxhoorn, D.M., Palliser, D., Mizuno, H., Yu, E.Y., An, D.S., Sabatini, D.M., Chen, I.S.Y., Hahn, W.C., Sharp, P.A., et al. (2003). Lentivirus-delivered stable gene silencing by RNAi in primary cells. RNA *9*, 493–501.

88. Claudi, F., Tyson, A.L., Petrucco, L., Margrie, T.W., Portugues, R., and Branco, T. (2021). Visualizing anatomically registered data with brainrender. eLife *10*, e65751.

89. De Rop, F.V., Ismail, J.N., Bravo González-Blas, C., Hulselmans, G.J., Flerin, C.C., Janssens, J., Theunis, K., Christiaens, V.M., Wouters, J., Marcassa, G., et al. (2022). Hydrop enables droplet-based single-cell ATAC-seq and single-cell RNA-seq using dissolvable hydrogel beads. eLife *11*, e73971.

CellPress
OPEN ACCESS

90. Raudvere, U., Kolberg, L., Kuzmin, I., Arak, T., Adler, P., Peterson, H., and Vilo, J. (2019). g:profiler: a web server for functional enrichment analysis and conversions of gene lists (2019 update). Nucleic Acids Res. *47*, W191–W198.

91. Delile, J., Rayon, T., Melchionda, M., Edwards, A., Briscoe, J., Sagner, A., Klein, A., and Treutlein, B. (2019). Single cell transcriptomics reveals spatial and temporal dynamics of gene expression in the developing mouse spinal cord. Development *146*, dev173807.

92. Angerer, P., Haghverdi, L., Büttner, M., Theis, F.J., Marr, C., and Buettner, F. (2016). destiny: diffusion maps for large-scale single-cell data in R. Bioinformatics *32*, 1241–1243.

93. Guilliams, M., Bonnardel, J., Haest, B., Vanderborght, B., Wagner, C., Remmerie, A., Bujko, A., Martens, L., Thoné, T., Browaeys, R., et al. (2022). Spatial proteogenomics reveals distinct and evolutionarily conserved hepatic macrophage niches. Cell *185*, 379–396.e38.

STAR★METHODS

KEY RESOURCES TABLE

REAGENT or RESOURCE	SOURCE	IDENTIFIER
Antibodies		
anti-Dach1, rabbit (1:400)	Proteintech	10914-1-AP
anti-GFP, chicken (1:3000)	Aves lab	Cat# GFP-1020, RRID:AB_10000240
anti-Meis2, mouse (1:500)	Sigma-Aldrich	Cat# WH0004212M1, RRID:AB_1842419
anti-NOS1, rabbit (1:1000)	Immunostar	Cat# 24287, RRID:AB_572256
anti-NOS1, rabbit (1:500)	Sigma-Aldrich	Cat# SAB4502010, RRID:AB_10744459
anti-NOS1, sheep (1:100)	Millipore	Cat# AB1529, RRID:AB_90743
anti-Parvalbumin, guinea pig (1:1000)	Synaptic Systems	195004<br>AB_2156476
anti-Pou3f2, mouse (1:400)	Santa Cruz	Cat# sc-393324, RRID:AB_2737347
anti-Somatostatin, rabbit (1:3000)	BMA Biomedicals	Cat# MAB354, RRID:AB_518614
anti-Somatostatin, rat (1:200)	Millipore	Cat# T-4103, RRID:AB_2255365
anti-Sox2, mouse (1:500)	Santa Cruz	Sc-365823<br>RRID:AB_10842165
anti-tdTomato, goat (1:500)	SICGEN	Cat# AB8181, RRID: AB_2722750
anti-tdTomato, guinea pig (1:500)	Oasis biofarm	OB-PGP004
Goat anti-Chicken, Alexa Fluor Plus 488	Thermo Fisher	Cat# A-32931, RRID:AB_2762843
Donkey anti-Guinea Pig, Alexa Fluor Plus 568	Thermo Fisher	Cat# A-11075, RRID:AB_2534119
Donkey anti-Guinea Pig, Alexa Fluor 647	Jackson ImmunoResearch	Cat# 706-605-148, RRID:AB_2340476
Goat anti-Mouse, Alexa Fluor 488	Thermo Fisher	Cat# A-21121, RRID:AB_2535764
Donkey anti-Rabbit, Alexa Fluor Plus 488	Thermo Fisher	Cat# A-21206, RRID: AB_2535792
Donkey anti-Rabbit, Alexa Fluor Plus 555	Thermo Fisher	Cat# A-32794, RRID:AB_2762834
Donkey anti-Rabbit, Alexa Fluor Plus 647	Thermo Fisher	Cat# A-32795, RRID: AB_2762835
Donkey anti-Rat, Alexa Fluor Plus 568	Thermo Fisher	Cat# A-78946
Goat anti-Rat Alexa Fluor 647	Thermo Fisher	Cat# A21247, RRID:AB_141778
Donkey anti-Sheep, Alexa Fluor Plus 488	Thermo Fisher	Cat# A11015, RRID: AB_2534082
Bacterial and virus strains		
AAV2/9-mDlx-FLEX-EGFP-WPRE-pA	Taitool Bioscience	S0978-9
AAV2/9-mDlx-FLEX-mScarlet-WPRE-pA	University of North Carolina - Chapel Hill Vector Core	Custom AAV2/9
Critical commercial assays		
10X Genomics Chromium 3' Single cell reagent Kit	10X Genomics	1000092
Chromium™ Chip B Single Cell Kit	10X Genomics	1000073
Chromium Fixed RNA Kit, Mouse Transcriptome	10X Genomics	1000469
Chromium Next GEM Single Cell Fixed RNA Sample Preparation Kit	10X Genomics	1000414
Deposited data		
scRNA-seq dataset (E16.5, P1, P5)	NCBI GEO	GSE217065
scRNA-seq dataset (P5 fixed cells)	NCBI GEO	GSE235619
Spatial transcriptomic dataset (P5)	NCBI GEO	GSE247005
Spatial transcriptomic dataset (P5), including processed data	Zenodo	https://zenodo.org/records/10069959

(*Continued on next page*)

Continued

REAGENT or RESOURCE	SOURCE	IDENTIFIER
Experimental models: Cell Lines		
HEK293T-Platinum-GP	Cell Biolabs	RV-103
Experimental models: Organisms/strains		
Mouse: *SST-Cre* [B6N.Cg-Ssttm2.1(cre)Zjh].	Taniguchi et al.[84]	JAX:018973, RRID:IMSR_JAX:018973
Mouse: *RCE* [(B6.Gt(ROSA)26Sortm1.1(CAG-EGFP)Fsh], also known as RCL^{eGFP}	Sousa et al.[85]	JAX:032037, RRID:MMRRC_032037-JAX
Mouse: $POU3f2^{fl/fl}$ [(Pou3f2$^{tm1\ Mejr}$]	Jaegle et al.[79]	RRID:MGI:4359761
Mouse: Dach1$^{fl/+}$[Dach1$^{tm1\ DaMi}$]	This paper	N/A
Mouse: IS [B6;129S4-Gt(ROSA)26Sortm3(CAG-tdTomato,-EGFP*)Zjh/J]	Madisen et al.[86]	JAX:028582
Oligonucleotides		
Pou3f2	Thermo Fisher	Mm00843777_s1
Pou3f3	Thermo Fisher	Mm00843792_s1
Sox2	Thermo Fisher	Mm03053810_s1
Rn18s	Thermo Fisher	Mm03928990_g1
Recombinant DNA		
p164-pAAV-mDlx-FLEX-mScarlet	This paper	https://www.addgene.org/204689/
p186-RV-CAG-dio-mScarlet-T2A-mVenus	This paper	https://www.addgene.org/204740/
p194-RV-CAG- dio-mScarlet-T2A-Brn2	This paper	https://www.addgene.org/204741/
p254-RV-CAG-dio-mScarlet-T2A-Dach1-202	This paper	https://www.addgene.org/204742/
pCMV-VSV-G	Stewart et al.[87]	https://www.addgene.org/8454/
Software and algorithms		
Code for the analysis of scRNA-seq and spatial transcriptomics datasets	This paper	https://zenodo.org/records/10069719
FIJI, ImageJ, Version 2.00-rc-69/1.52i	Open Software, ImageJ	RRID:SCR_002285 https://imagej.net/Fiji
NeuroInfo	MBF Bioscience	RRID:SCR_017346 https://www.mbfbioscience.com/products/neuroinfo
Polylux tool plugin (Molecular Cartography)	Resolve BioSciences	https://www.resolvebiosciences.com
Python Brainrender	Claudi et al.[88]	RRID:SCR_008394 https://www.python.org
R Studio	R Studio team	RRID:SCR_000432 https://www.rstudio.com/
Illustrator CC 2021	Adobe	RRID:SCR_010279, https://www.adobe.com/products/illustrator.html
QuantStudio 3 Real Time PCR System	Thermo Fisher	RRID:SCR_018712 https://www.thermofisher.com/order/catalog/product/A28567#/A28567

RESOURCE AVAILABILITY

Lead contact

Further information and requests for resources and reagents should be directed to the lead contact, Oscar Marín (oscar.marin@kcl.ac.uk).

Materials availability

Plasmids used to generate viruses in this study have been deposited on Addgene.

Data and code availability

- The scRNA-seq data and the analysis pipeline datasets have been deposited at the National Center for Biotechnology Information BioProjects Gene Expression Omnibus (GEO) and are accessible through GEO Series accession number GSE217065 (E16.5, P1, and P5 FACS data), GSE235619 (P5-fixed FACS) and GSE247005 (spatial transcriptomics). The processed spatial

transcriptomics dataset, including the region of interest used, is available at Zenodo (https://zenodo.org/records/10069959). All other original data reported in this paper will be shared by the lead contact upon request.

- The code used to analyse and process scRNA-seq and spatial transcriptomic data is available at Zenodo (https://zenodo.org/records/10069719).
- Any additional information required to reanalyze the data reported in this paper is available from the lead contact upon request.

EXPERIMENTAL MODEL AND STUDY PARTICIPANT DETAILS

Mice

Wild type and all transgenic mouse lines used in this study are listed in the key resources table. All adult mice were housed in groups and kept on a reverse light/dark cycle (12/12 h) regardless of genotypes. Only time-mated pregnant female mice were housed individually. Male and female mice were used in all experiments.

The following transgenic mouse lines (key resources table) were used in this study: *Pou3f2*$^{fl/+}$ [(Pou3f2tm1 Mejr],[79] *Dach1*$^{fl/+}$ [Dach1tm1 DaMi], *RCE* [(B6.Gt(ROSA)26Sortm1.1(CAG-EGFP)Fsh],[85] *IS* [B6;129S4-Gt(ROSA)26Sortm3(CAG-tdTomato,-EGFP) Zjh/J],[86] and *Sst*^{Cre} [B6N.Cg-Ssttm2.1(cre)Zjh].[84]

Sst^{Cre} mice were crossed with RCE reporter mice (JAX stock 032037) containing a floxed-stop cassette upstream of enhanced green fluorescent protein (EGFP) in the ROSA gene locus to obtain *Sst*^{Cre}*;RCE*$^{fl/+}$. To obtain *Sst*$^{Cre/+}$*;Pou3f2*$^{fl/+}$, *Pou3f2*$^{fl/+}$ mice were crossed with *Sst*$^{Cre/+}$ mice. To obtain *Sst*$^{Cre/+}$*;Dach1*$^{fl/+}$ mice, *Dach1*$^{fl/+}$ mice were crossed with *Sst*$^{Cr/+e}$ mice. To obtain *Sst*$^{Cre/+}$*;IS*$^{fl/+}$ mice, *Sst*$^{Cre/+}$ mice were crossed with IS reporter mice, which contain a floxed-stop cassette upstream of tdTomato in the ROSA gene locus. All mice were maintained on a C57Bl/6 background and housed under a 12 h light/dark cycle with ad libitum access to food and water.

All procedures were approved by KU Leuven, Tsinghua University, and King's College London animal welfare committees and were performed according to the project license of Directive 2010/63/EU, the Belgian Royal Decree, and the UK Home Office, respectively.

METHOD DETAILS

FACS experiments

Tissue preparation

All solutions used in this procedure have an osmolarity of 290 ± 20 mOsm/kg. The neocortex of E16.5, P1, and P5 *Sst*$^{Cre/+}$*;RCE* mice were dissected in an ice-cold HBSS buffer supplement with 25 mM of glucose. For E16.5, tissue from 6 animals was pooled together. For P1 and P5, tissue from 3 animals was pooled together. Tissues were cut into small pieces and digested for 15 min at 37°C with carbogen oxygenation using 10ml of pronaseE dissociation buffer solution, pH7.5, Osmolarity 300–315, with the following composition: 0.2 mg/ml pronaseE (Sigma), 50 mM of trehalose, 20 mM glucose, 0.8 mM kynurenic acid, 0.05 mM APV, 0.09 M Na^2SO^4, 0.03 M K^2SO^4 and 0.014 M $MgCl^2$. For P5 tissue, tissue was incubated for 10 mins with dissociation buffer containing pronaseE, after which 500 μl of 2.5% trypsin and 200 μl of 10mg/ml DNAseI were added and incubated at 37°C for 2 more minutes. Post enzymatic digestion, tissues were washed once in ice-cold dissociation buffer without enzyme and the tissue was mechanically triturated into single cells in 2 ml of OptiMEM (Invitrogen) supplement with 2% Trehalose, 20 mM glucose, 0.8 mM kynurenic acid and 0.05 mM APV using 3 glass fire polished pipettes with decreasing diameter. For P1 and P5 tissue, we removed myelin by overlaying single cell suspension on top of a 30% percoll and centrifuge at 700 g for 10 mins. The cell pellet was resuspended in 300-500 μl of OptiMEM with 2% Trehalose, 20 mM glucose, 0.8 mM kynurenic acid and 0.05 mM APV and tissue chunks were eliminated by passing cells suspension through a 40 μm cell strainer (BD).

Live cells FACS and scRNA-seq

GFP+ and DAPI negative cells were isolated using fluorescence-activated cell sorting (FACS) with a flow cytometer (Becton Dickinson) using a 100 μm nozzle, about 3000-5000 events per second, at 2-way purity, and collected directly in 1.5 ml (cold 4 deg) of OptiMEM/2% Trehalose solution in a 1.5-ml LoBind (Eppendorf) tubes precoated with 3% BSA overnight. We limited the FACS procedure to a maximum sort time of 40 mins to ensure high cell survival. We collected 20,000 to 30,000 cells for each time point, which is about 10% extra volume. We centrifuge the collect cells at 600g for 5 minutes at 4 deg and resuspend the FACS cells in 45 μl of OptiMEM/2%Trehalose. To determine cell number and viability, 5 μl of Trypan Blue was added to 5 μl of cells and cells counted on a hemacytometer. In all experiments, we only proceed toward scRNA-seq with > 80% viability. About 4000-6000 isolated single cells for each sample were loaded onto a 10X Genomic single-cell chip/machine for single-cell capture and cDNA library preparation. RNA-seq was performed in an Illumina high-seq 2500 platform.

Fixed cells FACS and scRNA-seq

Dissociated cells were incubated with Fixable Viability Dye eFluor 780 (ThermoFisher), 1:1000 for 5 minutes on ice. Cells were then transferred to 1.5-ml LoBind tubes precoated with 3% BSA overnight and centrifuged at 400g for 7 minutes. The Fixable Viability Dye was removed, and pellet cells were resuspended with 4% PFA (Fixation buffer) overnight at 4°C. The day after the cells were centrifuged at 850g for 7 minutes and resuspended in 700 μL of DPBS with 1% BSA, tissue chunks were eliminated by passing cells

suspension through a 40 μm cell strainer (BD). GFP+ and Fixable Viability Dye 780 negative cells were isolated using fluorescence-activated cell sorting (FACS) with a flow cytometer (BD FACS Aria Fusion) using a 100 μm nozzle, about 3000–5000 events per second, at 2-way purity, and collected at 4°C directly in 1.5-ml LoBind tube precoated with 3% BSA overnight. Post sort, quenching was done in accordance with the 10X Genomics demonstrated protocol (CG000478|Rev B), using reagents from the Chromium Fixed RNA Kit, Mouse Transcriptome kit (Cat No: 1000496). Briefly, 110,000 cells were collected and diluted in 875uL of Nuclease Free Water and 125 uL of Quenching Buffer, and 100 uL of Enhancer Buffer (provided Chromium Fixed RNA Kit) at 4° C. Before starting with probe hybridization, the cell count and quality assessments of the fixed cells were evaluated intermittently by performing cell counting on LUNA dual fluorescence cell counter (Logos Biosystems). Probe Hybridization, GEM barcoding, GEM recovery and pre-amplification, and library construction we followed the Chromium Fixed RNA Profiling protocol (Chromium Fixed RNA Profiling kit; CG000477|Rev C). For GEM generation, about 15000 isolated single cells were loaded onto a custom droplet encapsulation platform; HyDrop.[89] The Flex library was sequenced using BGI DNBSEQ-G400 using the following read configuration: r1: 28bps; i1:10; i2:10; r2: 90, with a sequencing depth of 43000 reads per cell.

Bioinformatic analyses

Data pre-processing

Sequencing data were prepared for analysis by application of the Cell Ranger pipeline. First, Illumina BCL output files were de-multiplexed into FASTQ format files. Feature counts were computed for individual GEM wells. STAR aligner was applied to perform splicing aware alignment to the GRCm38 reference genome, and then reads were bucketed into exonic, intronic, and intergenic categories. Reads were classified as confidently aligned if they corresponded to a single gene annotation. Only confidently aligned reads were carried forward to UMI counting. A cell calling algorithm in conjunction with the barcode rank plot was applied to filter low RNA content cells and remove empty droplets. The output is a read count matrix. We used the R package *Seurat* to perform the bulk of our subsequent data analysis, including filtering, normalization, scaling, and other downstream processing.

Quality control of scRNA-seq

We discarded any genes detected in fewer than 10 cells before filtering low-quality cells. Cells were carried forward if they met the following criteria: (i) unique gene count >700, (ii) mitochondrial gene content <10%, (iii) percentage confidently aligned reads > mean - 2s.d. (iv) ln(total UMI) > mean - 2s.d. Following this, other genes were removed if they fell into any of the following categories, mitochondrial genes, ribosomal genes, Y-chromosome genes. In live cell FACS experiments, we obtained 3,841 (E16.5), 2,418 (P1), and 2,895 (P5) single cells following quality control, for a total of 9,154 cells. In fixed cells FACS experiments, we recovered 11,477 cells (P5) following quality control.

Normalization and scaling

Normalization and scaling of reads were performed using the R package *Seurat*, with the functions *NormalizeData()* and *ScaleData()*. The normalized counts x_{norm} were computed by a logarithmic transformation of the raw counts, where $\boldsymbol{S} = 1000$ is a scaling factor and x_{tot} is the total read count for each cell.[22] Following integration, the data were linearly scaled and mean-centered.

Detecting variable features

Variable features were defined using the *Seurat* function *FindVariableFeatures()* with a variance-stabilizing transformation method, described in detail elsewhere.[61] We used the top 2,000 variable features as the highly variable genes.

Differentially expressed genes

Differentially expressed genes between clusters were computed by the Wilcoxon rank-sum test of one group against all remaining cells. We retained differentially expressed genes with greater than ln(1.5) log-fold change and with Bonferroni adjusted p-value < 0.01 unless stated otherwise. We use a notion of differential expression score as described before, defined for a set of genes size as

$$DEscore = \sum_{i=1}^{n} min\ (-\log_{10}(pi), 20)$$

where p_i is the Bonferroni-adjusted p-value of the differentially expressed gene. To find genes differentially expressed along the pseudotime, we applied the graph-based methodology implemented in *Monocle*. We identify cells at similar positions along a trajectory with similar gene expression by Moran's I test, a measure of spatial autocorrelation.

Multi-sample integration

Following quality control, individual samples were integrated into a single object using the anchoring procedure described in detail elsewhere.[61] In summary, the dimensionality of the data was reduced by canonical correlation analysis, and anchors comprised of a pair of cells – one from each data set – were computed. Anchors were chosen by a mutual nearest neighbor's approach, seeking to preserve cross-dataset correspondences. These anchors were scored, filtered, and weighted using nearest-neighbor graphs to arrive at a subset of anchors representing the shared structure between data sets. To preserve distinct heterogeneity evident in more mature cells, the samples were integrated pairwise in descending order of maturity. The data were then scaled, and variable features were computed.

The integrated data consists of two matrices. One is simply the aggregation of normalized counts from the three samples. The other is a batch-adjusted matrix produced as the integration output, taking both positive and negative values. Although this matrix has the benefit of being corrected for batch effects, it is abstracted from a direct notion of expression. Therefore, we restricted the use of this

corrected matrix to clustering, classification, and dimensionality reduction. We returned to the aggregate normalized count data when computing differentially expressed genes or otherwise comparing gene expression among groups of cells.

Dimensionality reduction

Principal components were computed using the *Seurat* function *RunPCA()* with the top 2000 variable features. We also compute t-distributed stochastic neighbour (TSNE) and uniform manifold approximation and projection (UMAP) embeddings using *RunTSNE()* and *RunUMAP()*, respectively, with the first 15 principal components.

Iterative clustering

To group cells into distinct transcriptomic clusters, we applied an iterative hierarchical clustering procedure, using a differential expression score to judge the validity of sub-cluster identities. The following steps were applied recursively, beginning with the entire data as a single cluster until clusters could not be further subdivided.

1. Compute variable genes and scale the data matrix.
2. Hierarchically cluster the data with average linkage.
3. For all integers : $2 \leq \boldsymbol{k} \leq 20$, compute the silhouette score for a dendrogram cut into k groups. Take a cut of the dendrogram into clusters $k_{max},$ where k_{max} is the value of $\boldsymbol{k}$ with highest silhouette score.
4. Compute differentially expressed genes for each cluster with a minimum logFC threshold of ln(2). Compute the differential expression score (DEscore) for each cluster.
5. Label clusters as failing qualifying criteria if they have a DEscore < 60 or have a size < 50 cells. If all clusters fail, cease subdivision, and return the initial input as a terminal identity.
6. Else, if qualifying clusters exist, merge failed clusters with the nearest qualifying cluster. Check that the newly merged clusters pass the DEscore threshold. If only one qualifying cluster remains, we have recovered the initial input cluster. Cease subdivision and return the initial input as a terminal identity.
7. If we have $N \geq 2$ qualifying clusters, apply steps 1–7 to each qualifying cluster with size $\geq$ 100. Otherwise, cease subdivision and return each qualifying cluster as a terminal identity.

Cluster validation

To validate cell identities, we performed five-fold cross-validation, taking 20% of the data as a test set and the remaining 80% as the training set. We computed differentially expressed genes for all clusters in the five training sets. We then proceed in a pairwise fashion, training a random forest classifier with 1,000 trees on each pair of clusters in the training data using only the top 10 differentially expressed genes from each cluster. This classifier was used to predict the identity of cells in the test set.

Following five-fold cross-validation, we have a predicted identity for every cell for each pairwise comparison of clusters. The above procedure was repeated for a total of 100 iterations. From the consistency of the outcome across these iterations, we extracted a measure of the stability of the cell classification. Over each pairwise comparison, we checked to see if one identity was entirely dominated by the other. For example, identity B was considered dominant if identity A was assigned to the cell 100/100 times and identity B was never assigned. We discounted all such dominated identities and retained only those which appear at least once in every one of their pairwise comparisons. The cell membership score to each cluster was computed as the proportion of cell assignments to that identity over all 10 iterations. A cell which had a score = 1 for one identity and = 0 for all others was referred to as a 'Core' cell. A cell with multiple identity ties (e.g., a score of 0.6 for cluster A and 0.4 for cluster B) was referred to as an 'Intermediate' cell and assigned the identity with the highest score. A cell for which all identities dominated or for which there was an evenly split score between identities was termed a 'Failed' classification. These represented a small proportion of the data ($\sim$ 0.2%).

Identifying neuronal subtypes

We used the dataset described by Tasic et al.[20] and Gouwens et al.[2] to identify cell types in our data. We used their Patch-seq dataset of adult cells from the mouse visual cortex labelled with MET-types and referred to their publication to infer cell type from morphology. Approximately 200 cells were labelled by MET-type, and we reannotated them as LRP neurons, MCs and nMCs. We computed DEGs between the three cell types, taking the ten with the highest log-fold change for each cell type as representative markers. We took these gene sets in combination with a short list of canonical markers and used them as identifying cell type markers.

To label cell types in the developmental data, we computed DEGs for each cluster. We computed DEscore for the detected markers of each cell type. The cell type with the highest DEscore was taken as the cluster identity. Any cluster with a DEscore > 10 for stress markers was discounted as a stressed group of dubious cell quality. Clusters with ambiguous expression of cell type markers were classified by MetaNeighbor alignment with MET-type annotated cells. They were assigned the cell type they most strongly correlated with.

MetaNeighbor analysis

Comparison of datasets to identify correlated cell groups was performed with the R package *Metaneighbor*.[22] AUROC scores for each pairwise comparison of clusters were produced by the correlation network approach implemented in the function *MetaNeighborUS()*. Clusters with AUROC > 0.75 represent a strong correlation between cell identities.

Functional enrichment analysis

To confirm the status of stressed cells and investigate cluster identities, we applied the R package *gProfileR*, as described elsewhere.[90] The function *gost()* was used to extract enriched GO terms from a list of differentially expressed genes.

CellPress
OPEN ACCESS

Data visualization

All data are plotted on R using the Seurat package. We have also created a Shiny web interface for data visualization (https://fisherj.shinyapps.io/sst-in-diversity/).

Gene modules

We computed characteristic gene modules to capture and describe the distinct sources of variation within the data. We used the R package *Antler* to generate these modules.[91] Computation began with a normalized counts-per-million matrix. Genes present in fewer than 10 cells, and cells with fewer than 700 genes were discounted from the computation. Genes were filtered prior to clustering to retain only those genes having Spearman correlation with at least 3 other genes at value 0.3. These genes were clustered hierarchically using Spearman correlation as a dissimilarity metric. This hierarchical clustering was repeated over many iterations, with a heuristic algorithm determining an optimal number of modules. At each iteration, modules were discarded if they failed to meet validity thresholds on the expression level of individual genes. At the terminal iteration, all modules met the validation criteria and were returned as the final set of gene modules. To understand the module contents, we performed functional enrichment on each module and examined gene ontology to identify module relevance to processes concerning development and cell type diversification.

Pseudotime

We used the R package *destiny* to compute the diffusion map,[92] which forms the basis of our pseudotime measure. To achieve a result describing cell type development, we only used the set of $\sim$200 genes from modules associated with cell type or cell maturity. The eigenvalues of the diffusion map transition matrix were taken as diffusion components for low-dimensional embedding. The pseudotime measure for each cell was computed from the full transition matrix using the *DPT()* function and represents a notion of diffusion distance from a root cell. From this, we inferred linear branches diverging from an initial shared state.

Spatial transcriptomics

Molecular cartography at 100-plex was performed according to the manufacturer's instructions and the imaging and spot detection pipeline described before.[93] Briefly, P5 tissue sections were dissected and fresh frozen by dry-ice and isopentane at -40°C. Coronal sections of 10 μm were placed within capture areas on Resolve BioScience slides and stored at -80°C till the day of sample processing (about 2 weeks). Tissue sections were thawed and fixed with 4% v/v Formaldehyde (Sigma-Aldrich F8775) in 1x PBS for 30 min at 4°C. After fixation, sections were washed twice in 1x PBS for two min, followed by 1 min washes in 50% Ethanol and 70% Ethanol at room temperature. Fixed samples were used for Molecular Cartography™ (100-plex combinatorial single-molecule fluorescence in-situ hybridization) according to the manufacturer's protocol 3.0; available for download from Resolve's website), starting with the aspiration of ethanol and the addition of buffer BST1 (step 6 and 7 of the tissue priming protocol). Tissues were primed, followed by overnight hybridization of all probes specific for the target genes (see Table S3 for target list). Samples were washed the next day to remove excess probes and fluorescently tagged in a two-step color development process. Regions of interest were imaged as described below, and fluorescent signals were removed during decolorization. Color development, imaging and decolorization were repeated for multiple cycles to build a unique combinatorial code for every target gene derived from raw images.

Samples were imaged on a Zeiss Celldiscoverer 7 (CD7), using the 50x Plan Apochromat water immersion objective with an NA of 1.2 and the 0.5x magnification changer, resulting in a 25x final magnification. LED excitation light source, filters, and dichroic mirrors were used with customized emission filters optimized for detecting specific signals per round and DAPI. Spot segmentation and downstream image analysis were performed with customized pipeline from Resolve Bioscience. The final image analysis was performed using Genexyz Polylux tool plugin from Resolve BioSciences to examine specific Molecular Cartography™ signals. The output data matrix consisted of an RNA count matrix of individual transcripts used to quantify expression, along with the metadata of cell location (X and Y position) and cortical anatomical regions - manually annotated based on reference developing mouse brain atlas.

AAV experiments

Mice (8-10 weeks old) were anaesthetized with isoflurane (induction 4%, maintenance 1%) in a stereotaxic apparatus (68018, RWD Life Science). The position of the head was carefully adjusted so that the skull surface was horizontal. The stereotaxic surgery was performed under a stereo microscope. The skull was exposed under antiseptic conditions, and a small craniotomy was made with a thin drill over the planned injection coordinates. Injections with a rate of 1nL/s through a borosilicate glass injection pipette (3-000-203-G/X, Drummond Scientific) attached to a pressure injection apparatus (Nanoject III Programmable Nanoliter Injector, Drummond Scientific). Mice were injected with 100nL of AAV2/9-mDlx-FLEX-EGFP or AAV2/9-mDlx-FLEX-mScarlet at the following stereotaxic coordinates based on the third edition of The Mouse Brain in Stereotaxic Coordinates by Franklin and Paxinos: +1.40mm anteroposterior (AP), +1.50mm mediolateral (ML) and $-$0.8mm (for P15 animals) and $-$1.0mm (for P56 animals). The pipette remained for 10 min at the end of infusion to allow virus diffusion.

Retrovirus experiments

Cloning of retroviral vectors

Pou3f2 and Dach1 were cloned by PCR into a conditionally expressing retroviral backbone, RV-CAG-dio-mScarlet-T2A, using XhoI and PacI restriction enzyme cloning sites, to create RV-CAG-dio-mScarlet-T2A-Pou3f2, RV-CAG-dio-mScarlet-T2A-Dach1 and control plasmid RV-CAG-dio-mScarlet-T2A-mVenus. Pou3f2 and Dach1 sequences were subcloned using transcript IDs ENSMUST00000178174.3 and ENSMUST00000071533.13.

Generation of retroviruses

Retroviruses were produced using HEK293T-PlatinumGP (Cell Biolabs) cells according to specification. In brief, HEK cells were grown in DMEM/10% FBS with 10 μg/ml Blasticidine (Merck 15205), plated at 2 x 10^6 cells per well in seven 15-cm plates. Upon 80% confluence, cells were split at a 1:1 ratio into two 4-layered CELLdisc of 1,000 cm^2, (Greiner Bio-One, cat no 678104). A day later, cells were transfected with 100 μg of retroviral transfer DNA construct (see key resources table) and 17 μg pCMV-VSV-G, and 351 μL of TransIt reagent (Mirus, Madison WI) in 160 ml of viral production medium consisting of Optimem (ThermoFisher) supplement with 0.2 mM sodium pyruvate, 5mM sodium butyrate, and 5% FBS. Forty-eight hours post-transfection, the first viral collection was done by removing the 160 ml of supernatant and vacuum filter with 0.4 μm membrane pore size and storing it at 4°C for 24 hours. An additional 160 ml of viral production medium was added for a second round of viral collection the next day. The two viral collections were pooled and concentrated by centrifugation at 20,000 g for 2 hours at 4°C and re-suspended in 100 μl of PBS and incubated for 30 mins on ice before centrifugation for 10 mins at 1600 g and 4°C. The viral-containing supernatant was removed, and 5 μl aliquots were stored at -80°C. Viral titers range from 1-5x10^9 IU/ml.

In utero retrovirus injections

In utero infections were performed at E14.5 *Nkx2.1-Cre* or E14.5 $Sst^{Cre/+}$ embryos. Timed-pregnant females were injected with 0.05 mg/kg buprenorphine hydrochloride and deeply anaesthetized with 2.5 % isoflurane. The abdominal cavity was opened to expose the uterus and 400 nL of concentrated retrovirus with 0.01% fast green (Sigma-Aldrich) were injected into the lateral ventricle of the telencephalon in each embryo using a glass capillary needle (3.5''Drummond 3-000-203Glx) coupled to a nanoinjector (World Precision Instruments). Glass needles were pulled using a vertical puller (World Precision Instruments). The uterine horns were then placed back in the abdominal cavity, and the abdominal wall was sutured (Ethicon coated Vicryl 4-0; V-4). The animals were placed in recovering chamber at 32°C for 1h post-surgery. Brain tissue was collected at P16 and processed for immunohistochemistry.

Histology and immunohistochemistry

Postnatal pups were deeply anaesthetized with an intraperitoneal injection of ketamine (87 mg per kg body weight) and xylazine (13 mg per kg body weight) and transcardially perfused with ice-cold phosphate-buffered saline (PBS) followed by 4% paraformaldehyde (PFA). The brains were isolated and incubated overnight in 4% PFA on a slow-speed rocking platform at 4 °C. Tissue was washed with PBS, incubated in 10% followed by 30% sucrose in PBS and cut on a freezing microtome into 25 μm, 45 μm, 60 μm or 100 μm coronal sections, and stored in cryoprotectant solution (30% glycerol, 30% ethylene glycol in PBS) at -20 °C or processed or free-floating immunohistochemistry. Coronal sections were stained using the following primary antibodies: rabbit anti-Dach1 (1:500, Proteintech, 10914-1-AP), chicken anti-GFP (1:3000, Aves Lab, GFP-1020), mouse anti-Meis2 (1:500, Sigma-Aldrich, WH0004212M1), rabbit anti-Nitric Oxide Synthase 1 (1:1000, Immunostar, 24287), rabbit anti-Nitric Oxide Synthase 1 (1:500, Sigma-Aldrich, SAB4502010), sheep anti-Nitric Oxide Synthase 1 (1:100, Millipore, AB1529), guinea-pig anti-Parvalbumin (1:1000, Synaptic Systems, 195004), mouse anti-Pou3f2 (1:400, Santa Cruz, sc-393324), rabbit anti-Somatostatin (1:3000, BMA Biomedicals, T-4103), rat anti-Somatostatin (1:200, Millipore, MAB354), mouse anti-Sox2 (1:500, Santa Cruz, Sc-365823), goat anti-tdTomato (1:500, SICGEN, AB8181), and guinea pig anti-tdTomato (1:500, Oasis biofarm, OB-PGP004). Antigen retrieval was used for Pou3f2 (Brn2), NOS1 and Dach1 immunohistochemistry. Sections were washed with PBS and incubated in Tris-EDTA buffer (10mM Tris 1mM EDTA, pH 9) for 10 minutes at 85 °C and cooled to RT for 30 min. Tissue was blocked with 2% BSA, 10% goat serum in 0.25% triton-X-100-PBS for 1h at RT. Primary antibodies were incubated overnight at 4 °C in 2% BSA, 10% goat serum in 0.25% triton-X-100-PBS. The following secondary antibodies conjugated to Alex Fluor dyes were used: goat anti-chicken IgY (H+L) 488, donkey anti-guinea pig IgG (H+L) 568, donkey anti-rabbit IgG (H+L) 488, donkey anti-rat IgG (H+L) 568, goat anti-rat IgG (H+L) 647, donkey anti-rabbit IgG (H+L) 555, donkey anti-rabbit IgG (H+L) 647, donkey anti-sheep IgG (H+L) 488, and goat anti-mouse IgG1 488 (all from Thermo Fisher, used at 1:1000) and donkey anti-guinea-pig IgG (H+L) 647 (Jackson ImmunoResearch, 1:250). Secondary antibodies were incubated for 2 h at RT followed by 4',6-diamidino-2-fenylindool (DAPI) (5 μM, Sigma, D9542) for 10 minutes at RT. In between steps, sections were washed three times for 10 minutes with 0.1% triton-X in PBS. Brain sections were mounted in Mowiol-DABCO (25% Mowiol, Sigma, 81381, 2,5% DABCO, Sigma, D27802). Brain sections were imaged using a Zeiss Cell Discoverer 8 LSM 900 confocal microscope (20x Apochromat/NA0.70 or 20x Apochromat/NA0.95) and an Olympus SpinSR10 spinning disk confocal super-resolution microscope (10x Apochromat/NA0.70 or 20x Apochromat/NA0.95). Subsequent image processing was performed using FIJI and NeuroInfo software (MBF Bioscience).

Quantitative polymerase chain reaction

The neocortex of P5 $Sst^{Cre/+}$;$Pou3f2^{fl/fl}$ and $Sst^{Cre/+}$;$Pou3f2^{+/+}$ littermate controls (n = 3 per genotype) were dissected and SST+ neurons were isolated using FACS as described above. cDNA libraries were processed for quantitative polymerase chain reaction (qPCR) using TaqMan™ Fast Advanced Master Mix (ThermoFisher) on a QuantStudio 3 Real Time PCR System (ThemoFisher) using the comparative Ct method. Probes against the following genes were used: Pou3f2 (mM00843777_s1, ThermoFisher), Pou3f3 (Mm00843792_s1, ThermoFisher), Sox2 (Mm03053810_s1, ThermoFisher) and housekeeping gene Rn18s (Mm03928990_g1, ThermoFisher). Conditional mutants were normalized to the average value detected in control animals.

QUANTIFICATION AND STATISTICAL ANALYSIS

Results were plotted and tested for statistical significance using Prism 9 or MATLAB. All values are indicated in Table S5.

CellPress
OPEN ACCESS

Cell density

Region of interest (ROI): motor cortex, somatosensory cortex and visual cortex were manually defined on P5 coronal sections. ROIs stained with reporter protein (EGFP or tdTomato) or specific markers were quantified using FIJI. P21 coronal sections were mapped unto the Allen Mouse Brain reference atlas (CCF v3) within the NeuroInfo software (MBF Bioscience) to define ROIs and quantify protein markers. The coordinates of each cell expressing NOS1 were output into a csv file and plotted with Python (Jupyter Notebook) using the package Brainrender[88] to obtain the 3D brain representation. Each animal was considered a biological replicate. For each brain, 12–18 images spanning rostral to caudal cortical regions were taken and treated as technical replicates. Data analyses were performed using R studio.

Axonal length

Forty-five-micron serial sections spanning rostral to caudal were collected. The regions of interest (ROI) were manually defined on sections with a segmented line using FIJI, and their length was counted. For each brain, images were taken along the rostrocaudal axis every 90 μm posterior to the injection site until about 3 mm (34 images for each brain in total) to count the axon length in the contralateral striatum and every 450 μm posterior to the injection site until about 3 mm (7 images for each brain in total) to quantify the axon length in the ipsilateral striatum. The axon length in each brain was the total sum of axon lengths in all these images, normalized by the thickness of the brain slices and the infected cells.

Sox2 levels

Sixty-micron sections were collected from the brains of $Sst^{Cre/+}$;$Pou3f2^{fl/fl}$ and $Sst^{Cre/+}$;$Pou3f2^{+/+}$ littermate controls. ROIs were manually defined based on NOS1 immunofluorescence together with DAPI for LRP neurons. The same approach was used to determine Sox2 levels in SST+ cells using $Sst^{Cre/+}$;*RCE* animals and ROIs were based on GFP immunofluorescence. Defined ROIs were projected on the Sox2 antibody signal, and the immunofluorescence intensity was measured using ImageJ. For each brain, images were taken spanning the motor cortex measuring 73 ± 14 cells/brain.

Dach1 and Pou3f2 overexpression experiments

Brains were sectioned at 100 μm and one in two sections were analyzed for each brain. Infected mScarlet+ cells from all neocortical regions were imaged at 20X using Apotome.2 microscopy (Zeiss), and the cells were identified as NOS1+ or NOS1-. In *Nkx2-1-Cre* mice, only the infected mScarlet+ cells lacking PV expression (thus putative SST+ cells) were analyzed.

Neuron, Volume *112*

Supplemental information

Cortical somatostatin long-range projection neurons and interneurons exhibit divergent developmental trajectories

Josephine Fisher, Marieke Verhagen, Zhen Long, Monika Moissidis, Yiming Yan, Chenyi He, Jingyu Wang, Elia Micoli, Clara Milían Alastruey, Rani Moors, Oscar Marín, Da Mi, and Lynette Lim

Neuron

Supplemental information

Cortical somatostatin long-range projection neurons and interneurons exhibit divergent developmental trajectories

Josephine Fisher, Marieke Verhagen, Zhen Long, Monika Moissidis, Yiming Yan, Chenyi He, Jingyu Wang, Elia Micoli, Clara Milían Alastruey, Rani Moors, Oscar Marín, Da Mi, Lynette Lim

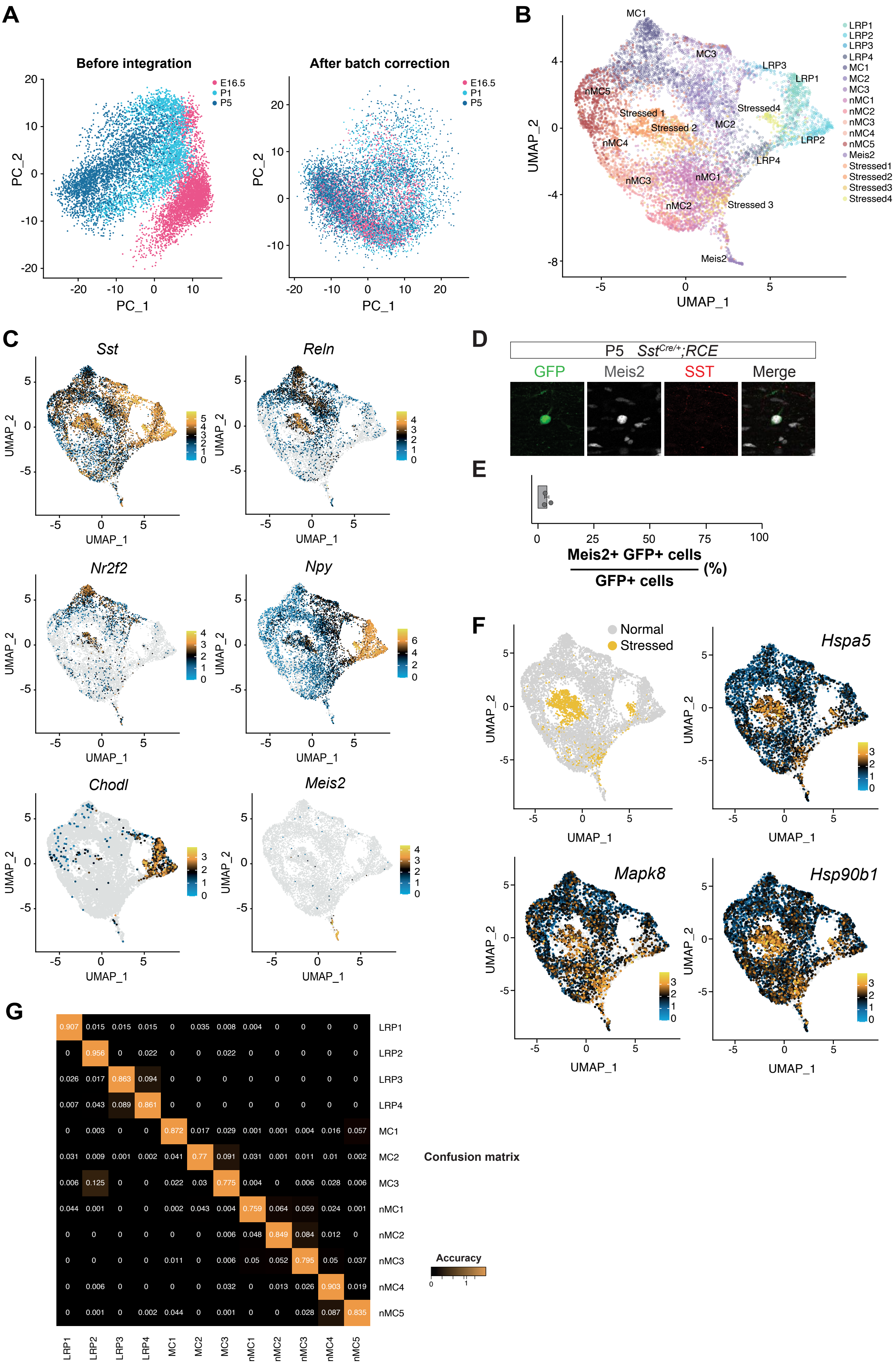
A
Before integration
After batch correction
E16.5
P1
P5
PC_1
PC_2
B
UMAP_1
UMAP_2
MC1 MC3 LRP3 LRP1 nMC5 Stressed 1 Stressed 2 Stressed4 MC2 LRP2 nMC4 LRP4 nMC3 nMC1 nMC2 Stressed 3 Meis2
LRP1 LRP2 LRP3 LRP4 MC1 MC2 MC3 nMC1 nMC2 nMC3 nMC4 nMC5 Meis2 Stressed1 Stressed2 Stressed3 Stressed4
C
Sst
Reln
Nr2f2
Npy
Chodl
Meis2
D
P5 Sst Cre/+;RCE
GFP
Meis2
SST
Merge
E
Meis2+ GFP+ cells
GFP+ cells
(%)
F
Normal
Stressed
Hspa5
Mapk8
Hsp90b1
G
Confusion matrix
Accuracy
LRP1: 0.907 0.015 0.015 0.015 0 0.035 0.008 0.004 0 0 0 0
LRP2: 0 0.956 0 0.022 0 0 0.022 0 0 0 0 0
LRP3: 0.026 0.017 0.863 0.094 0 0 0 0 0 0 0 0
LRP4: 0.007 0.043 0.089 0.861 0 0 0 0 0 0 0 0
MC1: 0 0.003 0 0 0.872 0.017 0.029 0.001 0.001 0.004 0.016 0.057
MC2: 0.031 0.009 0.001 0.002 0.041 0.77 0.091 0.031 0.001 0.011 0.01 0.002
MC3: 0.006 0.125 0 0 0.022 0.03 0.775 0.004 0 0.006 0.028 0.006
nMC1: 0.044 0.001 0 0 0.002 0.043 0.004 0.759 0.064 0.059 0.024 0.001
nMC2: 0 0 0 0 0 0 0.006 0.048 0.849 0.084 0.012 0
nMC3: 0 0 0 0 0.011 0 0.006 0.05 0.052 0.795 0.05 0.037
nMC4: 0 0.006 0 0 0 0 0.032 0 0.013 0.026 0.903 0.019
nMC5: 0 0.001 0 0.002 0.044 0 0.001 0 0 0.028 0.087 0.835
LRP1 LRP2 LRP3 LRP4 MC1 MC2 MC3 nMC1 nMC2 nMC3 nMC4 nMC5

Figure S1

Figure S1. scRNA-seq sample integration and quality control, related to Figure 1.
(A) Principal component analysis (PCA) embedding demonstrating batch-effect correction following multi-sample integration. Cells cluster by developmental stage before and by cell type after correction.
(B) Integration of SST+ neurons from E16.5, P1, and P5, and visualization by UMAP. The annotation of cell clusters was based on marker expression.
(C) Examples of gene expression of markers for LRP neurons, MCs and nMCs visualized by UMAP.
(D) High-magnification of the neocortex of $Sst^{Cre/+}$*;RCE* mice at P5 showing expression of GFP, Meis2 and SST – the GFP neuron expresses Meis2 but not SST.
(E) Quantification of the fraction of Meis2+ and GFP+ cells among GFP+ cells in the neocortex of $Sst^{Cre/+}$*;RCE* mice at P5 (*n* = 3 mice).
(F) Integration of SST+ neurons from E16.5, P1, and P5, and visualization by UMAP with stressed cells highlighted in yellow. The feature plots depict ER-stress markers enriched in the group of stressed cells.
(G) The confusion matrix depicts the accuracy cell assignment in the random forest model for each SST+ subtype.

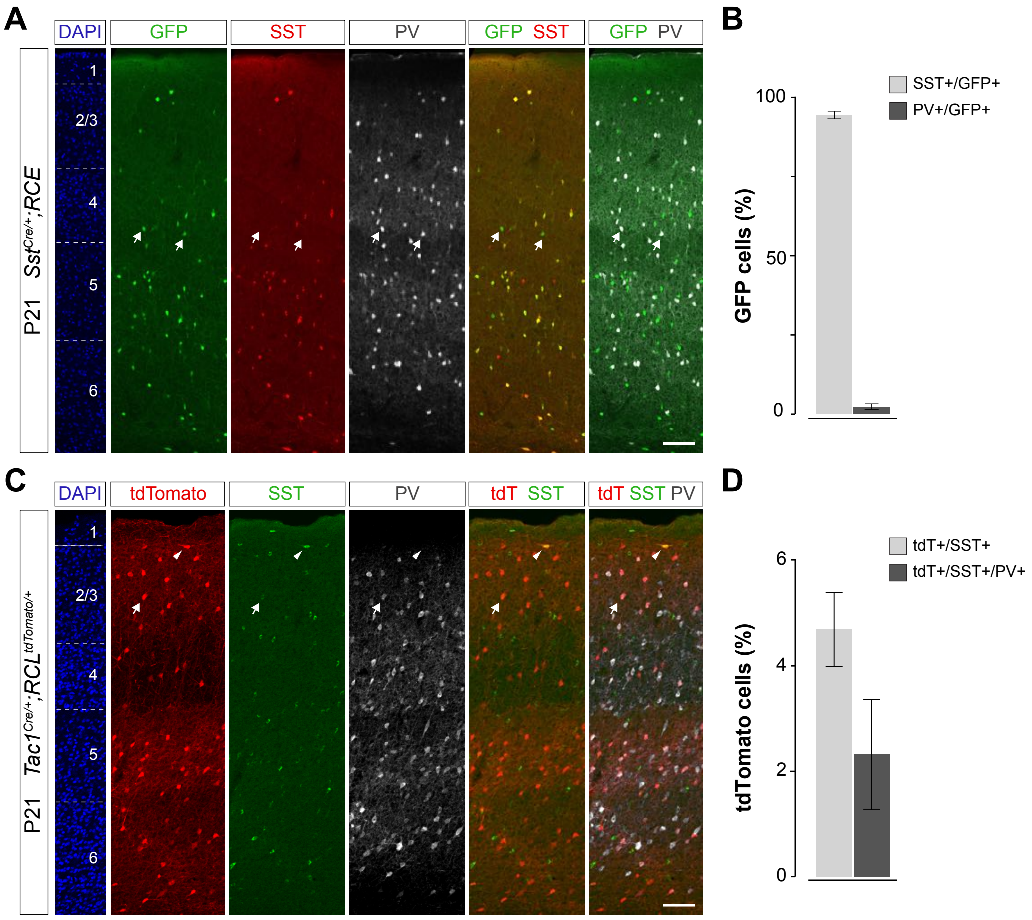

A
DAPI
GFP
SST
PV
GFP SST
GFP PV
P21 Sst^Cre/+;RCE
1
2/3
4
5
6
B
SST+/GFP+
PV+/GFP+
GFP cells (%)
100
50
0
C
DAPI
tdTomato
SST
PV
tdT SST
tdT SST PV
P21 Tac1^Cre/+;RCL^tdTomato/+
1
2/3
4
5
6
D
tdT+/SST+
tdT+/SST+/PV+
tdTomato cells (%)
6
4
2
0

Figure S2

Figure S2. *Sst^{Cre};RCE* mice label SST+ neurons with great specificity, related to Figure 1.
(A) Coronal sections through the neocortex of *Sst$^{Cre/+}$;RCE* mice at P21 stained with antibodies against GFP, SST, and PV. DAPI staining reveals the distribution of nuclei. Arrowheads indicate GFP+/PV+/SST- cells.
(B) Quantification of the fraction of SST+/GFP+ and PV+/GFP+ cells among GFP+ cells in the somatosensory cortex of *Sst$^{cre/+}$;RCE* mice (*n* = 4 mice) at P21.
(C) Coronal sections through the neocortex of *Tac1$^{Cre/+}$;RCL$^{tdTomato/+}$* mice at P21 stained with antibodies against tdTomato, SST, and PV. DAPI staining reveals the distribution of nuclei. Arrowheads indicate a tdT+ SST+ PV- cell; arrows indicate a tdT+ SST+ PV+ cell.
(D) Quantification of the fraction of SST+/tdT+ and PV+/SST+/tdT+ cells among tdT+ cells in the somatosensory cortex of *Tac1$^{Cre/+}$;RCL$^{tdTomato/+}$* mice (*n* = 2 mice) at P21.
Data are shown as mean ± s.e.m. Scale bars, 50 μm.

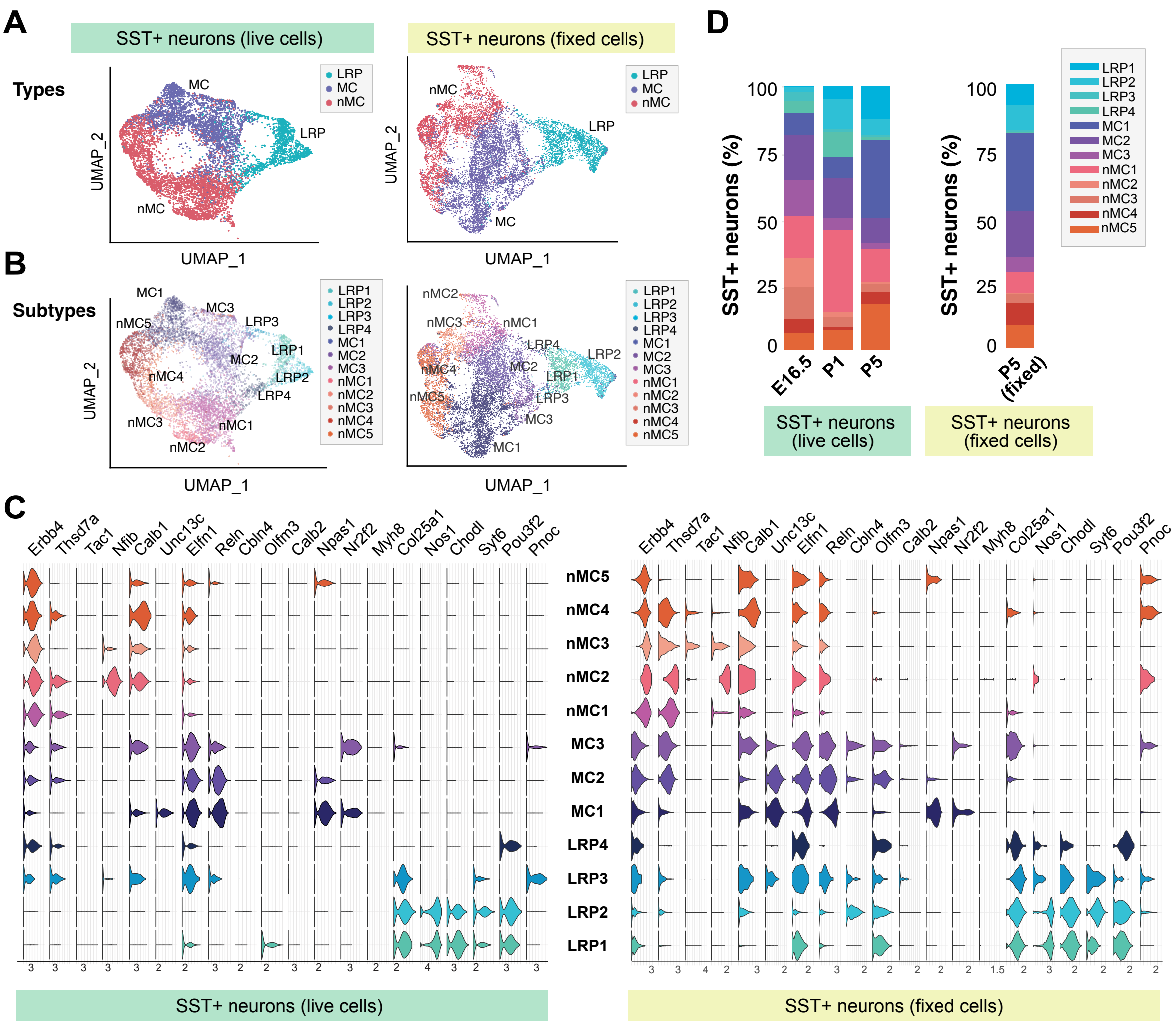
A
SST+ neurons (live cells)
SST+ neurons (fixed cells)
Types
B
Subtypes
UMAP_1
UMAP_2
LRP
MC
nMC
LRP1
LRP2
LRP3
LRP4
MC1
MC2
MC3
nMC1
nMC2
nMC3
nMC4
nMC5
C
Erbb4
Thsd7a
Tac1
Nfib
Calb1
Unc13c
Elfn1
Reln
Cbln4
Olfm3
Calb2
Npas1
Nr2f2
Myh8
Col25a1
Nos1
Chodl
Syt6
Pou3f2
Pnoc
D
SST+ neurons (%)
E16.5
P1
P5
P5 (fixed)
SST+ neurons (live cells)
SST+ neurons (fixed cells)

Figure S3

Figure S3. SST+ subtype diversity of live-FACS cells matches well with fixed-FACS cells at P5, related to Figure 2.
(A and B) Clustering of cortical SST+ cells into types (A) and subtypes (B) in the E16.5, P1 and P5 dataset of live-FACS cells and the P5 fixed-FACS. In the latter case, clusters were reannotated using the classifier models described in Figure 1.
(C) Distribution of each subtype in the E16.5, P1, and P5 dataset (live-FACS cells) and the fixed-FACS P5 dataset.
(D) Violin plots of selected cell type markers comparing SST+ cells in the E16.5, P1, and P5 dataset (live-FACS) and the fixed-FACS P5 dataset.

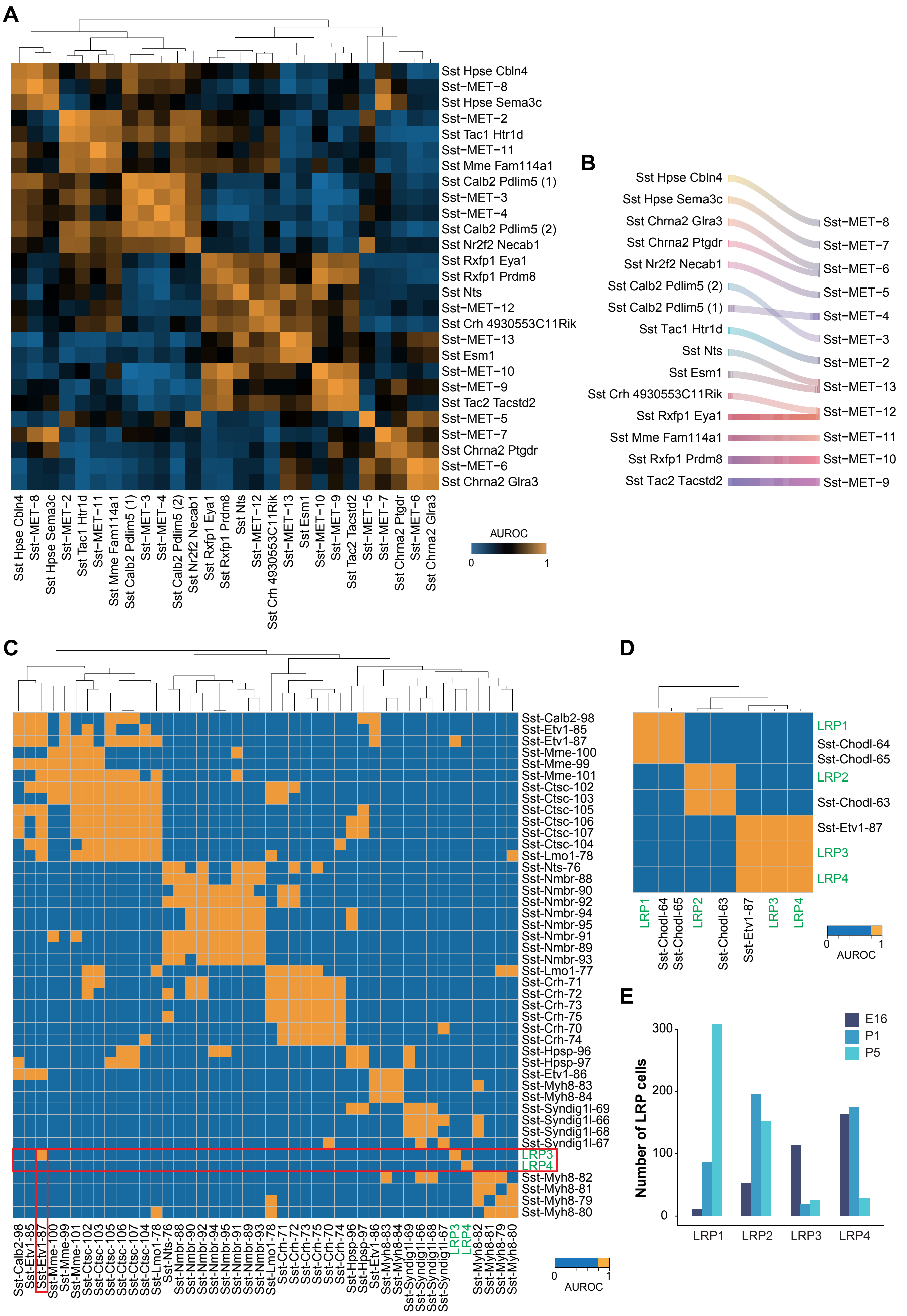

Figure S4

Figure S4. MetaNeighbor analyses between adult and developmental datasets, related to Figure 3.

(A) Heatmap illustrating transcriptomic similarities (AUROC values) between adult transcriptomic SST+ cell clusters[20], and adult SST+ MET subtypes[2].

(B) River plot illustrating the correspondence of adult transcriptomic SST+ cell clusters and Sst-MET types using an AUROC value larger than 0.75.

(C) MetaNeighbor analysis illustrating transcriptomic similarities (AUROC values) between the developing SST+ clusters LRP3 and LRP4 and all adult transcriptomic SST+ subtypes identified by Yao et al. (2021)[29].

(D) MetaNeighbor analysis illustrating transcriptomic similarities (AUROC values) between LRP1 to 4 clusters and four adult Sst subtypes (Sst-Chodl-63, Sst-Chodl-64, Sst-Chodl-65, Sst-Etv1-87).

(E) Histograms illustrating the distribution of SST+ LRP cell clusters by developmental stage.

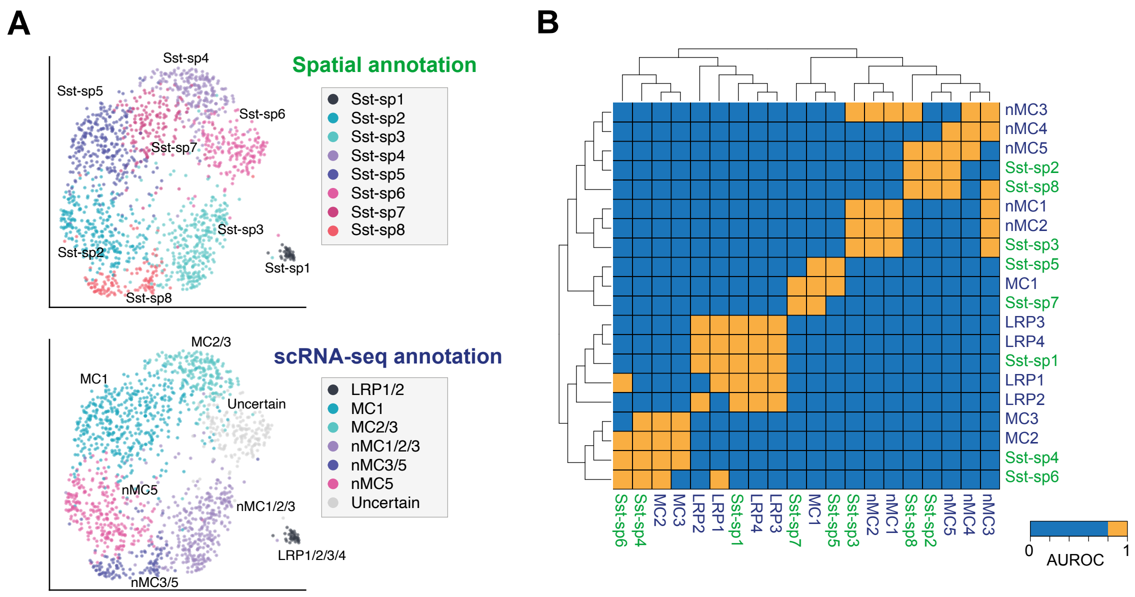
A
B
Spatial annotation
Sst-sp1
Sst-sp2
Sst-sp3
Sst-sp4
Sst-sp5
Sst-sp6
Sst-sp7
Sst-sp8
scRNA-seq annotation
LRP1/2
MC1
MC2/3
nMC1/2/3
nMC3/5
nMC5
Uncertain
LRP1/2/3/4
AUROC
0
1

Figure S5

Figure S5. Alignment of scRNA-seq and spatial transcriptomics datasets, related to Figure 5.

(A) Integration of P5 cortical SST+ cells from the spatial transcriptomic (top panel) and scRNA-seq (bottom panel) datasets, and visualization by UMAP. Annotation of scRNA-seq clusters was based on marker expression, while annotation of spatial clusters was based on label transfer from the scRNA-seq dataset.

(B) MetaNeighbor analysis illustrating transcriptomic similarities between P5 scRNA-seq and spatial transcriptomic clusters.

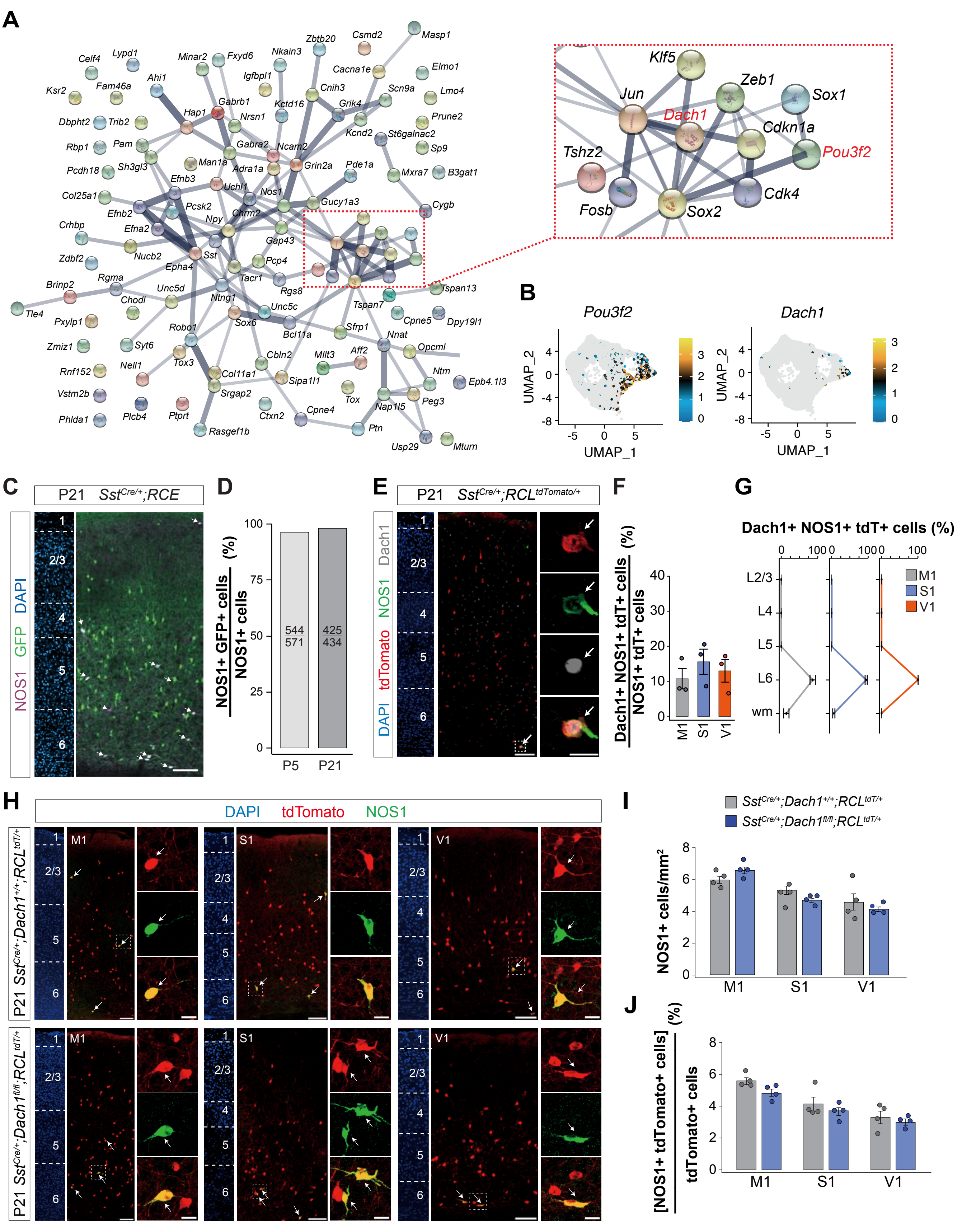

A
B
Pou3f2
Dach1
UMAP_1
UMAP_2
C
P21 SstCre/+;RCE
NOS1 GFP DAPI
D
NOS1+ GFP+ cells / NOS1+ cells (%)
544/571
425/434
P5
P21
E
P21 SstCre/+;RCLtdTomato/+
DAPI tdTomato NOS1 Dach1
F
Dach1+ NOS1+ tdT+ cells / NOS1+ tdT+ cells (%)
G
Dach1+ NOS1+ tdT+ cells (%)
M1
S1
V1
H
DAPI tdTomato NOS1
P21 SstCre/+;Dach1+/+;RCLtdT/+
P21 SstCre/+;Dach1fl/fl;RCLtdT/+
I
SstCre/+;Dach1+/+;RCLtdT/+
SstCre/+;Dach1fl/fl;RCLtdT/+
NOS1+ cells/mm2
J
[NOS1+ tdTomato+ cells] / tdTomato+ cells (%)

Figure S6

Figure S6. Genes differentially expressed genes in LRP neurons and function of Dach1 in LRP development, related to Figures 6 and 7.

(A) STRING network analysis for genes differentially expressed between SST+ LRP neurons and SST+ interneurons (Table S3). Full STRING network, medium confidence (0.4), size cut-off of no more than ten interactors. The inset highlights a cluster of transcription factors at the core of this network.

(B) The feature plots depict the expression of two genes, *Dach1* and *Pou3f2*, which are highly enriched and almost exclusively expressed by LRP neurons.

(C) Coronal sections through the neocortex of $Sst^{Cre/+}$*;RCE* mice P21 stained with antibodies against NOS1 and GFP. DAPI staining reveals the distribution of nuclei.

(D) Quantification of the fraction of NOS1+ and GFP+ cells among GFP+ cells in the neocortex of $Sst^{Cre/+}$*;RCE* mice at P5 and P21 (*n* = 3 mice for each stage).

(E) Expression of Dach1 in cortical LRP neurons in primary motor cortex (M1), primary somatosensory cortex (S1), and primary visual cortex (V1) of $Sst^{Cre/+}$;$RCL^{tdT/+}$ mice at P21 stained with antibodies against NOS1, Dach1, and tdTomato. DAPI staining reveals the distribution of nuclei.

(F) Quantification of the fraction of NOS1+ and Dach1+ cells among cortical SST+ neurons (*n* = 3 mice). Student *t*-test: not significant.

(G) Laminar distribution of Dach1+/NOS1+/tdTomato+ cells in M1, S1 and V1 of $Sst^{Cre/+}$;$RCL^{tdT/+}$ mice at P21 (*n* = 3 mice).

(H) Coronal sections through the neocortex of control and *Dach1* conditional mutant mice at P21 stained with antibodies against NOS1 and tdTomato. Arrows indicate NOS1+ and tdTomato+ cells. DAPI staining reveals the distribution of nuclei.

(I and J) Quantification of the density and proportion of NOS1+ and tdTomato+ neurons in M1, S1, and V1 of control and *Dach1* conditional mutant mice at P21 (*n* = 4 mice per genotype). Two-way ANOVA: not significant.

Data are shown as mean ± s.e.m. Scale bars, 100 µm and 20 µm (inserts).

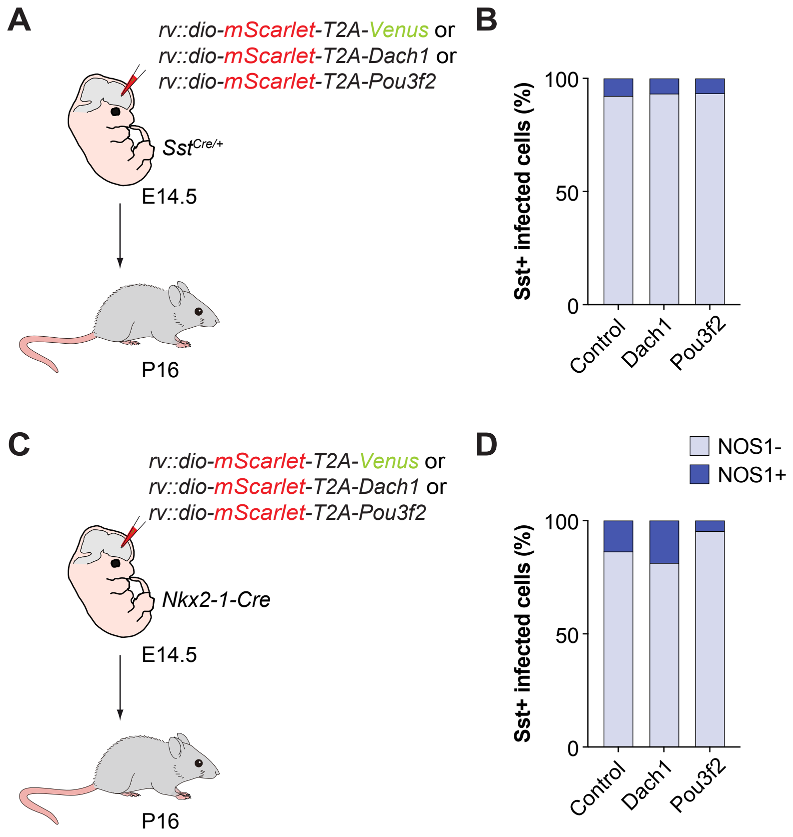

A
rv::dio-mScarlet-T2A-Venus or
rv::dio-mScarlet-T2A-Dach1 or
rv::dio-mScarlet-T2A-Pou3f2
Sst Cre/+
E14.5
P16
B
Sst+ infected cells (%)
100
50
0
Control
Dach1
Pou3f2
C
rv::dio-mScarlet-T2A-Venus or
rv::dio-mScarlet-T2A-Dach1 or
rv::dio-mScarlet-T2A-Pou3f2
Nkx2-1-Cre
E14.5
P16
D
NOS1-
NOS1+
Sst+ infected cells (%)
100
50
0
Control
Dach1
Pou3f2

Figure S7

Figure S7. Expression of Dach1 and Pou3f2 does not induce NOS1 expression, related to Figures 6 and Figure 7.
(A) Schematic of the experimental design.
(B) Quantification of the fraction of NOS1- and NOS1+ cells among infected mScarlet+ cells in the neocortex of $Sst^{Cre/+}$ mice at P16. Control (*mScarlet-T2A-mVenus*: 192 NOS1- cells and 17 NOS1+ cells); *mScarlet-T2A-Dach1*: 175 NOS1- cells and 13 NOS1+ cells; *mScarlet-T2A-Pou3f2*: 149 NOS1- cells and 11 NOS1+ cells. Chi-square test: $p = 0.8635$.
(C) Schematic of the experimental design.
(D) Quantification of the fraction of NOS1- and NOS1+ cells among infected mScarlet+ cells in the neocortex of *Nkx2-1-Cre/+* injected animals at P16. Control (*mScarlet-T2A-mVenus*: 78 NOS1- cells and 13 NOS1+ cells; *mScarlet-T2A-Dach1*: 50 NOS1- cells and 13 NOS1+ cells; *mScarlet-T2A-Pou3f2*: 20 NOS1- cells and 1 NOS1+ cells. Chi-square test: $p = 0.1968$.

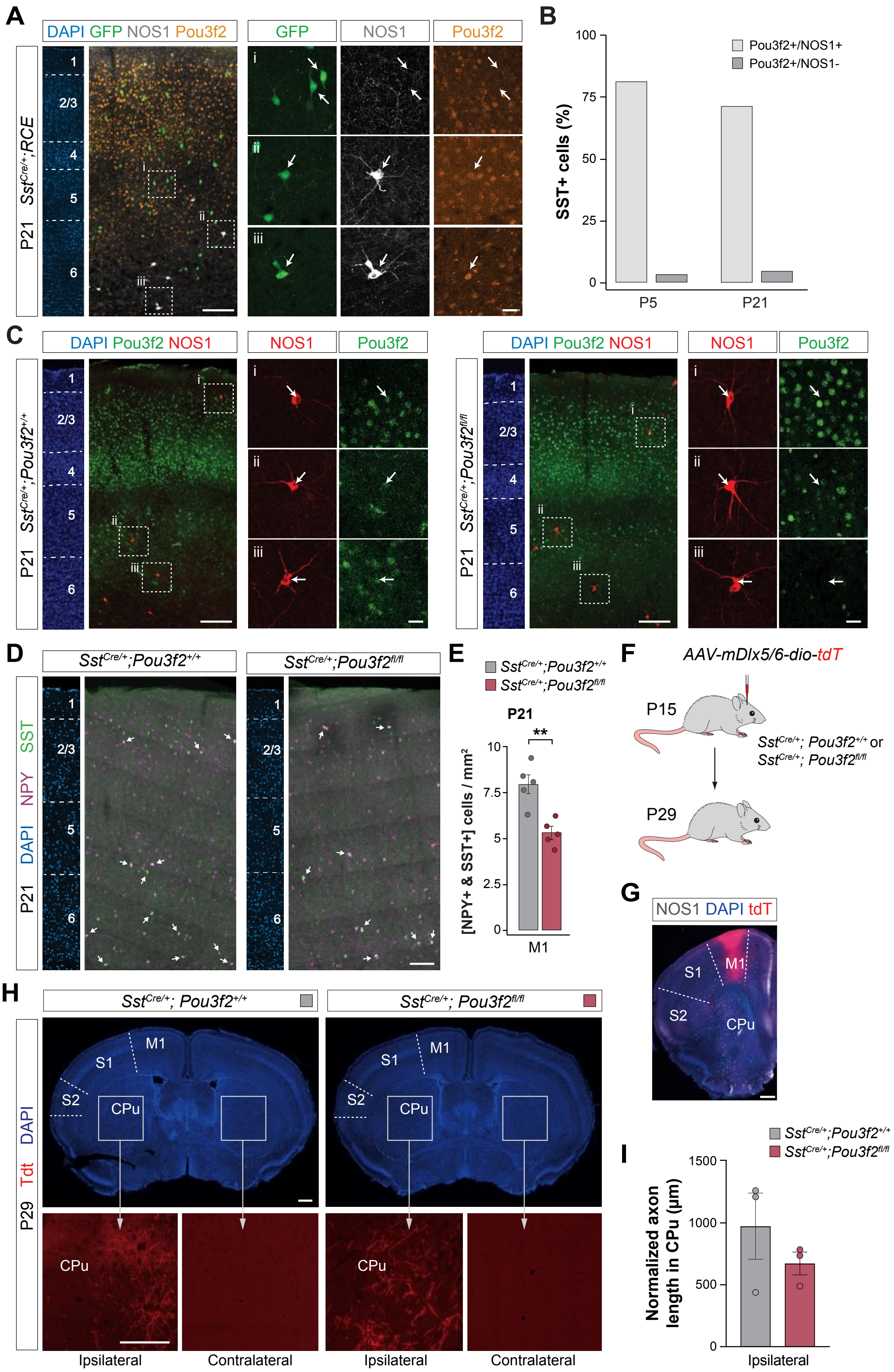

A
DAPI GFP NOS1 Pou3f2
GFP
NOS1
Pou3f2
P21 SstCre/+;RCE
B
Pou3f2+/NOS1+
Pou3f2+/NOS1-
SST+ cells (%)
P5
P21
C
DAPI Pou3f2 NOS1
P21 SstCre/+;Pou3f2+/+
P21 SstCre/+;Pou3f2fl/fl
D
SstCre/+;Pou3f2+/+
SstCre/+;Pou3f2fl/fl
P21 DAPI NPY SST
E
P21
[NPY+ & SST+] cells / mm2
M1
F
AAV-mDlx5/6-dio-tdT
P15
SstCre/+; Pou3f2+/+ or
SstCre/+; Pou3f2fl/fl
P29
G
NOS1 DAPI tdT
M1
S1
S2
CPu
H
SstCre/+; Pou3f2+/+
SstCre/+; Pou3f2fl/fl
P29 Tdt DAPI
Ipsilateral
Contralateral
I
Normalized axon length in CPu (µm)
Ipsilateral

Figure S8

Figure S8. Expression of Pou3f2 in cortical LRP neurons, related to Figure 7.
(A) Coronal sections through the neocortex of $Sst^{Cre/+}$*;RCE* mice at P5 and P21 stained with antibodies against NOS1, Pou3f2, and GFP. DAPI staining reveals the distribution of nuclei.
(B) Quantification of the fraction of Pou3f2+/NOS1+ cells and Pou3f2+/NOS1- cells among cortical SST+ neurons.
(C) Coronal sections through the neocortex of control and *Pou3f2* conditional mutant mice at P21 stained with antibodies against NOS1 and Pou3f2. DAPI staining reveals the distribution of nuclei.
(D) Coronal sections through the mouse telencephalon stained with antibodies against NPY and SST in control and *Pouf3f2* conditional mutants at P21. DAPI staining reveals the distribution of nuclei.
(E) Quantification of the density of SST+/NPY+ cells in the motor cortex at P21 (n = 5 per genotype). Student t-test with Bonferroni: $^{**}p < 0.01$.
(F) Schematic of the experimental design for axonal labeling.
(G) Coronal section through the injection site stained with antibodies against tdTomato and NOS1. DAPI staining reveals the distribution of nuclei.
(H) Coronal sections through the mouse telencephalon stained with antibodies against tdTomato in control and *Pou3f2* conditional mutants at P29. DAPI staining reveals the distribution of nuclei. The high-magnification images illustrate axons in the striatum (CPu).
(I) Quantification of total axonal length in the ipsilateral striatum normalized by the number of NOS1+/tdTomato+ cells in each animal (n = 3 mice per genotype).
Data are shown as mean ± s.e.m. Scale bars, 500 µm (G and H), 100 µm (A, C and D), and 20 µm (insets).

Table S1. Differentially expressed genes among SST+ neurons, related to Figure 1

Marker Gene	Cell type Enrichment	Marker Gene	Cell type Enrichment
CHRNA2	Martinotti	SFRP1	LRP
CALB2	Martinotti	POU3F2	LRP
NPAS1	Martinotti	DACH1	LRP
CBLN4	Martinotti	SYT6	LRP
MYH8	Martinotti	HHIP	LRP
HTR1A	Martinotti	CHODL	LRP
ELFN1	Martinotti	NOS1	LRP
GPX3	Martinotti	CHRM2	LRP
NR2F2	Martinotti	PTN	LRP
SATB1	Martinotti	COL25A1	LRP
ETV1	Martinotti	NPY	LRP
HPSE	Martinotti	CRHBP	LRP
RELN	Martinotti	TRPM3	LRP
UNC13C	Martinotti	CPNE4	LRP
TMEFF2	Martinotti	EGFEM1	LRP
DPP10	Martinotti	CLU	Stressed
OLFM3	Martinotti	APP	Stressed
KIRREL3	Martinotti	CPE	Stressed
NELL1	Martinotti	PSAP	Stressed
HS6ST3	Martinotti	PDIA6	Stressed
PCDH7	Martinotti	ATP2C1	Stressed
CDH12	Martinotti	HSPA5	Stressed
NTS	Non-Martinotti	CALR	Stressed
TAC2	Non-Martinotti	P4HB	Stressed
TAC1	Non-Martinotti	HSP90B1	Stressed
TH	Non-Martinotti	XBP1	Stressed
PTPRD	Non-Martinotti	CDH13	Stressed
C1QL3	Non-Martinotti		
CRH	Non-Martinotti		
THSD7A	Non-Martinotti		
ERBB4	Non-Martinotti		
GABRA1	Non-Martinotti		
KCNH7	Non-Martinotti		
CNTNAP2	Non-Martinotti		
LRP1B	Non-Martinotti		
KCNIP4	Non-Martinotti		
CNTN5	Non-Martinotti		
CSMD1	Non-Martinotti		
GABRG3	Non-Martinotti		
LRRC4C	Non-Martinotti		

Table S2. Genes in developmental modules, related to Figure 4

Gene symbol	Gene symbol	Gene symbol	Gene symbol
2900055J20RIK	ABLIM1	GNG4	SLC24A2
AHI1	ABLIM3	GRIN1	SLC24A3
ARFGEF3	ACHE	GRIN2A	SNAP91
ATP6V0A1	ADCY1	GRIN2B	SNCA
B230209E15RIK	ADCY2	GRIN3A	SPARCL1
CDKN1A	ADCYAP1R1	IGF1	SRGAP3
CLCN3	AF529169	ILDR2	SSX2IP
CPNE5	AI413582	INPP5F	STUM
CRHBP	AI504432	KCNMA1	SV2A
DLG2	AKAP7	KCNQ3	SYNGR1
FXYD6	ALCAM	KIF1A	SYNPR
GABRB1	AP2A2	KIF5A	SYP
GAP43	ATP1A3	KIFC2	SYT13
GRM1	ATP1B1	MAP1A	THRA
HAP1	ATP2A2	MAP2K4	TMEM151A
KCND2	B4GALT6	MAP6	TMOD2
L1CAM	BEND6	MAPK10	TRIM37
LSAMP	CACNG2	MRFAP1	TRPC5
MEG3	CADM2	MTPN	TSPAN17
NCAM2	CADM3	NAV2	UNC13C
NRXN1	CAMK2B	NCDN	YWHAB
NTM	CAMK2N1	NDRG4	YWHAZ
OPCML	CAMK4	NPTXR	
PCLO	CBARP	NRGN	
PEG3	CD99L2	NRIP3	
PFKP	CDK5R2	NRXN2	
PKIA	CDKL2	PAFAH1B1	
PLPPR4	CLSTN1	PDYN	
PSAP	CNTN1	PEA15A	
PTPRS	CTNND2	PGM2L1	
RIMS1	CX3CL1	PITPNC1	
SCN3B	DNAJC5	PPP2R2C	
SEZ6L2	DPYSL5	PRKCG	
SNHG11	EIF4G2	PTPRN	
SPOCK2	EMC10	RASGRF2	
STXBP6	ENO2	REEP5	
SYN1	FAM171B	RELL2	
SYT11	FKBP1B	RGS6	
SYT4	FUT9	ROBO2	
TENM1	FXYD7	SCAMP5	
TIMP2	GABBR2	SCN2A	
TMEM130	GAS7	SDC3	
TSPAN7	GDA	SH3GL2	
1500009L16RIK	GNAL	SHISA6	
2900011O08RIK	GNAO1	SHROOM2	
2900097C17RIK	GNAZ	SLC12A5	

Table S4. Genes used in spatial transcriptomics experiments, related to Figure 5

Gene symbol	Gene symbol	Gene symbol	Gene symbol
ADRA1A	ERBB4	MYH8	RGS6
ALKAL2	ETV1	NDST4	RXFP1
AMELX	GABRA2	NETO1	SATB1
APELA	GAD1	NFIB	SFRP1
APP	GAD2	NOS1	SHISA9
B3GAT1	GNG4	NPAS1	SLC24A2
BCL6	GPHN	NPAS3	SLC30A3
BMP3	GPX3	NPY	SORCS1
CALB2	HAP1	NR2F2	SOX1
CBLN4	HHIP	PAX6	SP9
CDH8	HPSE	PBX3	SPON1
CHODL	ID2	PCP4	SST
CHRNA2	ILDR2	PCP4L1	SYP
CHRNA4	INHBB	PDE1A	SYT6
CLU	KCNIP2	PDYN	TAC1
COL19A1	KHDRBS3	POU3F2	TACR1
COL25A1	LHX6	PSAP	TACR3
CORT	LHX8	PTN	TH
DACH1	LRP1B	RARB	THSD7A
DBPHT2	LYPD1	RASGEF1B	TRIB2
DLX1	LYPD6	RBFOX3	UNC5C
DLX5	MEIS2	RELN	ZEB2
ELFN1	MGAT4C	RGMA	

Table S5. Summary of data and statistical analyses, related to Figures 5-8 and Figures S2, S6, S7 and S8

**Fig. 5**	Measurement	Values	N	Statistical	P value
Fig. 5E	Fraction of cells in superficial and deep layers	LRP1/2: 5.4 and 94.6; Sst-MET-1: 31.2 and 68.8; MC1: 2.5 and 97.5; Sst-MET-6/7: 0 and 100; MC2/3: 37.9 and 62.1; Sst-MET-3/4: 63.4 and 36.6; nMC1/2/3: 20.8 and 79.2; Sst-MET2: 88.2 and 11.8; nMC3/5: 0 and 100; Sst-MET-9/10/13: 0 and 100	[total cells] LRP1/2 = 37<br>Sst-MET-1 = 16<br>MC1 = 160<br>Sst-MET-6/7 = 29<br>MC2/3 = 211<br>Sst-MET-3/4 = 41<br>nMC1/2/3 = 312<br>Sst-MET2 = 17<br>nMC3/5 = 116<br>Sst-MET-9/10/13 = 40	Fisher exact test	LRP1/Sst-MET-1 = 0.029<br>MC1/2/Sst-MET-6/7 = 1<br>MC2/3/ Sst-MET-3/4 = 0.0032<br>nMC1/2/3/ Sst-MET-2 = 0.00001<br>nMC3/5/ Sst-MET-9/10/13 = 1
**Fig. 6**	Measurement	Values	N	Statistical	P value
Fig. 6D	Ipsilateral axon length per cell in CPu at P70 (mean ± SEM)	$Sst^{Cre/+}$;$Dach1^{+/+}$: 104.74 ± 4.21;<br>$Sst^{Cre/+}$;$Dach1^{fl/fl}$: 115.12 ± 5.94	[brains] n = 6 for each genotype	Unpaired t-test	p = 0.1844
Fig. 6D	Contralateral axon length per cell in CPu at P70 (mean ± SEM)	$Sst^{Cre/+}$;$Dach1^{+/+}$: 0.78 ± 0.44;<br>$Sst^{Cre/+}$;$Dach1^{fl/fl}$: 7.05 ± 1.78	[brains] n = 6 for each genotype	Unpaired t-test	p = 0.0066
Fig. 6E	Ipsilateral axon length per cell in CPu at p29 (mean ± SEM)	$Sst^{Cre/+}$;$Dach1^{+/+}$: 106.62 ± 7.35;<br>$Sst^{Cre/+}$;$Dach1^{fl/fl}$: *118.91* ± 4.27	[brains] n = 6 for each genotype	Unpaired t-test	p = 0.1789
Fig. 6E	Contralateral axon length per cell in CPu at P29 (mean ± SEM)	$Sst^{Cre/+}$;$Dach1^{+/+}$: *0.14* ± 0.04;<br>$Sst^{Cre/+}$;$Dach1^{fl/fl}$: *3.16* ± 0.46	[brains] n = 6 for each genotype	Unpaired t-test	p < 0.0001
**Fig. 7**	Measurement	Values	N	Statistical	P value
Fig. 7B	Mean Density of Nos1+ cells at P5 (mean ± SEM)	$Sst^{cre/+}$;$Pou3f2^{+/+}$: M1, 12.69 ± 1.05; S1, 20.31 ± 1.36; V1, 12.21 ± 0.95.<br>$Sst^{Cre/+}$;$Pou3f2^{fl/fl}$: M1, 13.69 ± 1.25; S1, 19.55 ± 1.25; V1, 12.39 ± 0.74	[brains] n = 5 for each genotype	t-test (Mean Density~Genotype), post-hoc by Bonferroni correction	M1, p = 1; S1, p = 1; V1, p = 1
Fig. 7B	Mean Density of Nos1+ cells at P21 (mean ± SEM)	$Sst^{Cre/+}$;$Pou3f2^{+/+}$: M1, 8.17 ± 0.44; S1, 8.03 ± 0.35; V1, 6.87 ± 0.72.<br>$Sst^{Cre/+}$;$Pou3f2^{fl/fl}$: M1, 5.61 ± 0.22; S1, 6.25 ± 0.32; V1, 4.45 ± 0.42	[brains] n = 6 for each genotype	t-test (Mean Density~Genotype), post-hoc by Bonferroni correction	M1, p = 0.0032; S1, p = 0.012; V1, p = 0.047
Fig. 7D	Normalized Mean Density of Nos1+ cells in MCx at P5, P10, P14 and P21 (mean ± SEM)	$Sst^{Cre/+}$;$Pou3f2^{+/+}$: P5, 1 ± 0.08; P10, 1 ± 0.05; P14, 1 ± 0.03; P21, 1 ± 0.05.<br>$Sst^{Cre/+}$;$Pou3f2^{fl/fl}$: P5, 1.07 ± 0.09; P10, 0.87 ± 0.04; P14, 0.80 ± 0.03; P21, 0.68 ± 0.02	[brains] n = 5 for each genotype at P5, 4 for each genotype at P10, 3 for each genotype at P14 and 6 for each genotype at P21	t-test (Mean Density~Genotype), post-hoc by Bonferroni correction	P5, p = 1; P10, p = 0.39; P14, p = 0.048; P21, p = 0.0032
Fig. 7D	Normalized Mean Density of Nos1+ cells in SSCx at P5, P10, P14 and P21 (mean ± SEM)	$Sst^{Cre/+}$;$Pou3f2^{+/+}$: P5, 1 ± 0.06; P10, 1 ± 0.02; P14, 1 ± 0.01; P21, 1 ± 0.04.<br>$Sst^{Cre/+}$;$Pou3f2^{fl/fl}$: P5, 0.96 ± 0.06; P10, 0.86 ± 0.04; P14, 0.77 ± 0.01; P21, 0.77 ± 0.04	[brains] n = 5 for each genotype at P5, 4 for each genotype at P10, 3 for each genotype at P14 and 6 for each genotype at P21	t-test (Mean Density~Genotype), post-hoc by Bonferroni correction	P5, p = 1; P10, p = 0.17; P14, p = 0.002; P21, p = 0.012
Fig. 7D	Normalized Mean Density of Nos1+ cells in VCx at P5, P10, P14 and P21 (mean ± SEM)	$Sst^{Cre/+}$;$Pou3f2^{+/+}$: P5, 1 ± 0.07; P10, 1 ± 0.06; P14, 1 ± 0.07; P21, 1 ± 0.08.<br>$Sst^{Cre/+}$;$Pou3f2^{fl/fl}$: P5, 0.99 ± 0.06; P10, 0.81 ± 0.05; P14, 0.71 ± 0.008; P21, 0.68 ± 0.05	[brains] n = 5 for each genotype at P5, 4 for each genotype at P10, 3 for each genotype at P14 and 6 for each genotype at P21	t-test (Mean Density~Genotype), post-hoc by Bonferroni correction	P5, p = 1; P10, p = 0.23; P14, p = 0.18; P21, p = 0.047
**Fig. 8**	Measurement	Values	N	Statistical	P value
Fig. 8B	Sox2 level intenstiy (mean ± SEM)	$Sst^{Cre/+}$;*RCE*: SST+NOS1+, 20,340 ± 762.8; SST+;NOS1-, 3,184 ± 76.1	[brains] n= 3	Unpaired t-test	p < 0.00001
Fig. 8D	Sox2 level intensity at P5 (mean ± SEM)	$Sst^{Cre/+}$;$Pou3f2^{+/+}$: NOS1+: 1223 ± 23;<br>$Sst^{Cre/+}$;$Pou3f2^{fl/fl}$: NOS1+: 899 ± 60	[brains] n = 3 for each genotype	Unpaired t-test	p = 0.0075
Fig. 8E	Normalized comparative Ct: Pou3f2, Pou3f3, Sox2 at P5 (%) (mean ± SEM)	$Sst^{Cre/+}$;$Pou3f2^{+/+}$: Pou3f2, 100; Pou3f3, 100; Sox2, 100.<br>$Sst^{Cre/+}$;$Pou3f2^{fl/fl}$: Pou3f2, 40.5 ± 0.5; Pou3f3, 125.5 ± 11.5; Sox2, 70.5 ± 11.5	[brains] n = 3 for each genotype	Unpaired t-test	Pou3f2: p = 0.00047; Pou3f3, p = 0.25; Sox2, p = 0.0015
**Fig. S2**	Measurement	Values	N	Statistical	P value
Fig. S2B	GFP+SST+ cells and GFP+PV+ cells at P21 (%)	GFP+SST+ cells: 90.2 ± 2.0%; GFP+PV+ cells: 2.6 ± 1.4%	[brains] n = 4	N/A	
Fig. S2D	tdTomato+SST+ cells and tdTomato+SST+PV+ cells at P21 (%)	tdT+SST+ cells: 4.6 ± 0.7%; tdT+SST+ PV+ cells: 2.32 ± 1.04%	[brains] n = 2	N/A	
**Fig. S6**	Measurement	Values	N	Statistical	P value

Fig. S6D	Fraction of NOS1+ cells expressing SST at P5 and P21 (%)	*Sst*$^{Cre/+}$*;RCE*: P5, 95.27; P21, 97.92	[cells] n = 571 Nos1+, 544 Nos1+; GFP+ at P5 and n =434 Nos1+, 425 Nos1+;GFP+ at P21	N/A	
Fig. S6F	Proportion of Dach1+;Nos+;tdTomato+ cells among Nos+;tdTomato cells at P21 (%) (mean ± SEM)	*Sst*$^{Cre/+}$*;RCL*$^{tdT/+}$: M1, 10.73 ± 2.90; S1, 15.57 ± 3.60; V1, 12.99 ± 3.23	[brains] n=3 for each cortical area	One-way ANOVA, Bonferroni's multiple comparisons test	M1 vs. S1 : *p* = 0.9998 M1 vs. V1 : p > 0.9999 S1 vs. V1 : p > 0.9999
Fig. S6G	Ratio of Dach1+;Nos1+;tdTomato+ cells in each layer at P21 (%) (mean ± SEM)	*Sst*$^{Cre/+}$*;RCL*$^{tdT/+}$: M1, L2/3: 0%; L4: 0%; L5: 0%; L6: 91.41% ± 4.82%; WM: 8.59% ± 4.82%; S1, L2/3: 0%; L4: 0%; L5: 0%; L6: 91.90% ± 4.23%; WM: 8.10% ± 4.23%; V1, L2/3: 0%; L4: 0%; L5: 0%; L6: 100.00% ; WM: 0%.	[brains] n=3 for each cortical area	N/A	
Fig. S6I	Mean Density of NOS1+ cells at P21 (mean ± SEM)	*Sst*$^{Cre/+}$*;Dach1*$^{+/+}$: M1, 6.02 ± 0.24; S1, 5.07 ± 0.31; V1, 4.62 ± 0.59. *Sst*$^{Cre/+}$*;Dach1*$^{fl/fl}$: M1, 6.24 ± 0.46; S1, 4.60 ± 0.15; V1, 4.26 ± 0.14	[brains] n = 4 for each genotype	Two-way ANOVA, Bonferroni's multiple comparisons test	M1 : *p* = 0.4240 S1 : *p* = 0.5307 V1 : *p* > 0.9999
Fig. S6J	Proportion of NOS1+;tdTomato+ cells at P21 (%) (mean ± SEM)	*Sst*$^{Cre/+}$*;Dach1*$^{+/+}$: M1, 5.63 ± 0.22; S1, 4.12 ± 0.46; V1, 3.31 ± 0.41. *Sst*$^{Cre/+}$*;Dach1*$^{fl/fl}$: M1, 4.87 ± 0.23; S1, 3.65 ± 0.25; V1, 3.01 ± 0.18	[brains] n = 4 for each genotype	Two-way ANOVA, Bonferroni's multiple comparisons test	M1 : *p* = 0.3057 S1 : *p* = 0.9091 V1 : *p* > 0.9999
**Fig. S7**	Measurement	Values	N	Statistical	P value
Fig. S7B	Fraction of infected mScarlet+ cells expressing NOS1 at P16 (%)	Control: 91.87% NOS1-, 8.13% NOS1+; Dach1: 93.09% NOS1-, 6.91% NOS1+; Pou3f2: 93.13% NOS1-, 6.87% NOS1+	[cells] Control: n = 192 Nos1- and n = 17 Nos1+; Dach1: n = 175 Nos1- and n = 13 Nos1+; Pou3f2: n = 149 Nos1- and n = 11 Nos1+.	Chi-square test	*p* = 0.8635
Fig. S7D	Fraction of infected mScarlet+ PV- cells expressing NOS1 at P16 (%)	Control: 85.71% NOS1-, 14.29% NOS1+; Dach1: 79.37% NOS1-, 20.63% NOS1+; Pou3f2: 95.24% NOS1-, 4.76% NOS1+	[cells] Control: n = 78 Nos1- and n = 13 Nos1+; Dach1: n = 50 Nos1- and n = 13 Nos1+; Pou3f2: n = 20 Nos1- and n = 1 Nos1+;	Chi-square test	*p* = 0.1988
**Fig. S8**	Measurement	Values	N	Statistical	P value
Fig. S8B	Fraction of NOS1+;SST+ cells or SST+ cells expressing Pou3f2 at P5 and P21 (%)	*Sst*$^{Cre/+}$*;RCE:* NOS1+SST+, P5, 81.19; P21, 70.90. SST+ P5, 3.13; P21: 4.34.	[cells] n = 1787 SST+ and n = 234 Nos1+ at P5 and n = 899 SST+ and n = 110 Nos1+ at P21	N/A	
