# Supplementary material for: Cortical somatostatin long-range projection neurons and interneurons exhibit divergent developmental trajectories
Source: Neuron. Author manuscript; Available in PMC 2025 Oct 11. (PMC7618239; doi:10.1016/j.neuron.2023.11.013)
Supplement: Supplemental Information — can be found online at https://doi.org/10.1016/j.neuron.2023.11.013. [file EMS209514-supplement-Supplemental_Information.zip › 1-s2.0-S0896627323008875-mmc1.pdf]

Neuron, Volume *112*

Supplemental information

Cortical somatostatin long-range projection neurons and interneurons exhibit divergent developmental trajectories

Josephine Fisher, Marieke Verhagen, Zhen Long, Monika Moissidis, Yiming Yan, Chenyi He, Jingyu Wang, Elia Micoli, Clara Milían Alastruey, Rani Moors, Oscar Marín, Da Mi, and Lynette Lim

Neuron

Supplemental information

Cortical somatostatin long-range projection neurons and interneurons exhibit divergent developmental trajectories

Josephine Fisher, Marieke Verhagen, Zhen Long, Monika Moissidis, Yiming Yan, Chenyi He, Jingyu Wang, Elia Micoli, Clara Milían Alastruey, Rani Moors, Oscar Marín, Da Mi, Lynette Lim

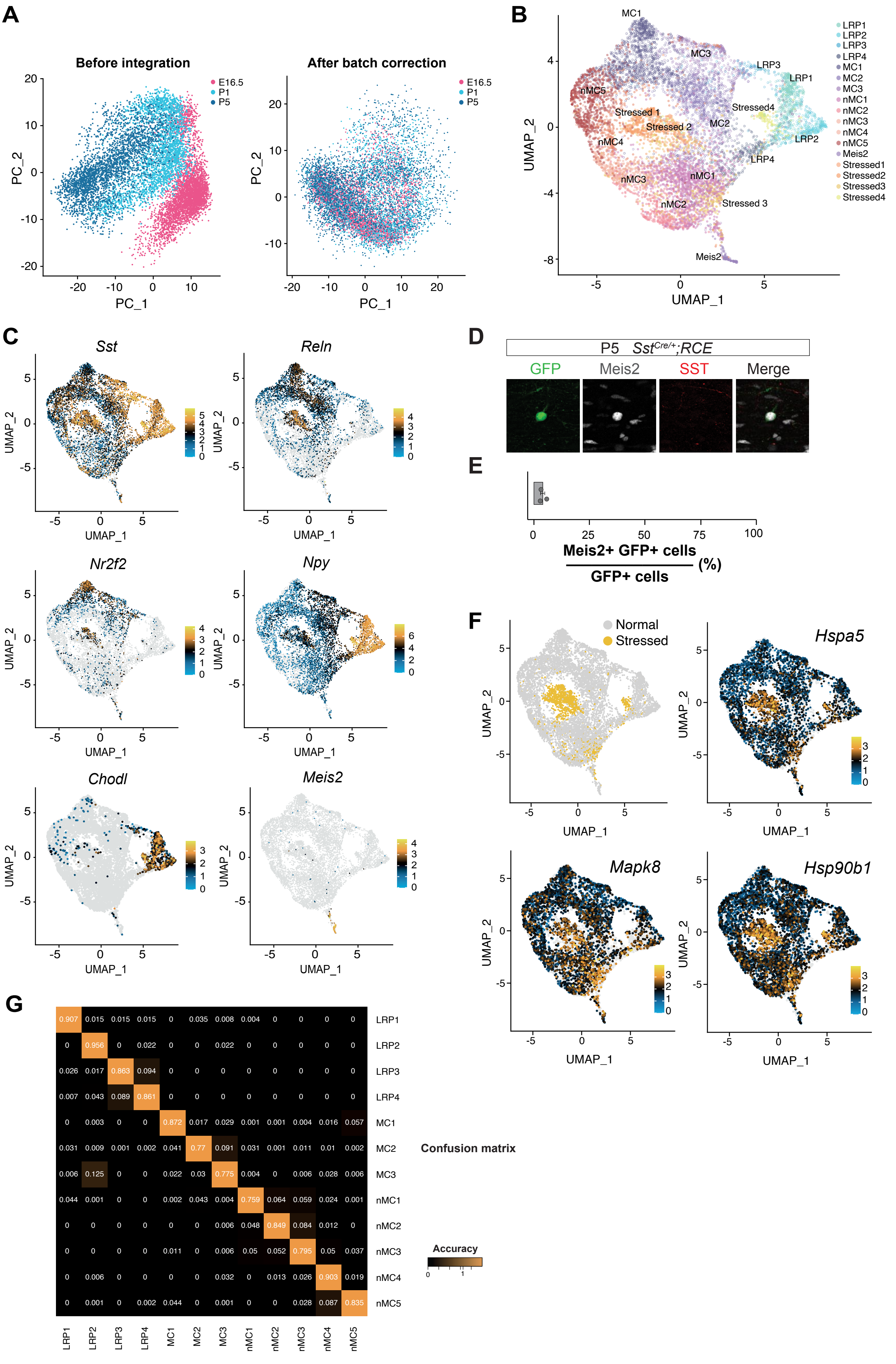
A
Before integration
After batch correction
E16.5
P1
P5
PC_1
PC_2
B
UMAP_1
UMAP_2
MC1
MC3
LRP3
LRP1
nMC5
Stressed 1
Stressed 2
Stressed4
MC2
LRP2
nMC4
LRP4
nMC3
nMC1
nMC2
Stressed 3
Meis2
LRP1
LRP2
LRP3
LRP4
MC1
MC2
MC3
nMC1
nMC2
nMC3
nMC4
nMC5
Meis2
Stressed1
Stressed2
Stressed3
Stressed4
C
Sst
Reln
Nr2f2
Npy
Chodl
Meis2
D
P5 Sst Cre/+;RCE
GFP
Meis2
SST
Merge
E
Meis2+ GFP+ cells
GFP+ cells
(%)
F
Normal
Stressed
Hspa5
Mapk8
Hsp90b1
G
0.907 0.015 0.015 0.015 0 0.035 0.008 0.004 0 0 0 0 LRP1
0 0.956 0 0.022 0 0 0.022 0 0 0 0 0 LRP2
0.026 0.017 0.863 0.094 0 0 0 0 0 0 0 0 LRP3
0.007 0.043 0.089 0.861 0 0 0 0 0 0 0 0 LRP4
0 0.003 0 0 0.872 0.017 0.029 0.001 0.001 0.004 0.016 0.057 MC1
0.031 0.009 0.001 0.002 0.041 0.77 0.091 0.031 0.001 0.011 0.01 0.002 MC2
0.006 0.125 0 0 0.022 0.03 0.775 0.004 0 0.006 0.028 0.006 MC3
0.044 0.001 0 0 0.002 0.043 0.004 0.759 0.064 0.059 0.024 0.001 nMC1
0 0 0 0 0 0 0.006 0.048 0.849 0.084 0.012 0 nMC2
0 0 0 0 0.011 0 0.006 0.05 0.052 0.795 0.05 0.037 nMC3
0 0.006 0 0 0 0 0.032 0 0.013 0.026 0.903 0.019 nMC4
0 0.001 0 0.002 0.044 0 0.001 0 0 0.028 0.087 0.835 nMC5
LRP1 LRP2 LRP3 LRP4 MC1 MC2 MC3 nMC1 nMC2 nMC3 nMC4 nMC5
Confusion matrix
Accuracy

Figure S1

Figure S1. scRNA-seq sample integration and quality control, related to Figure 1.
(A) Principal component analysis (PCA) embedding demonstrating batch-effect correction following multi-sample integration. Cells cluster by developmental stage before and by cell type after correction.
(B) Integration of SST+ neurons from E16.5, P1, and P5, and visualization by UMAP. The annotation of cell clusters was based on marker expression.
(C) Examples of gene expression of markers for LRP neurons, MCs and nMCs visualized by UMAP.
(D) High-magnification of the neocortex of $Sst^{Cre/+}$*;RCE* mice at P5 showing expression of GFP, Meis2 and SST – the GFP neuron expresses Meis2 but not SST.
(E) Quantification of the fraction of Meis2+ and GFP+ cells among GFP+ cells in the neocortex of $Sst^{Cre/+}$*;RCE* mice at P5 (*n* = 3 mice).
(F) Integration of SST+ neurons from E16.5, P1, and P5, and visualization by UMAP with stressed cells highlighted in yellow. The feature plots depict ER-stress markers enriched in the group of stressed cells.
(G) The confusion matrix depicts the accuracy cell assignment in the random forest model for each SST+ subtype.

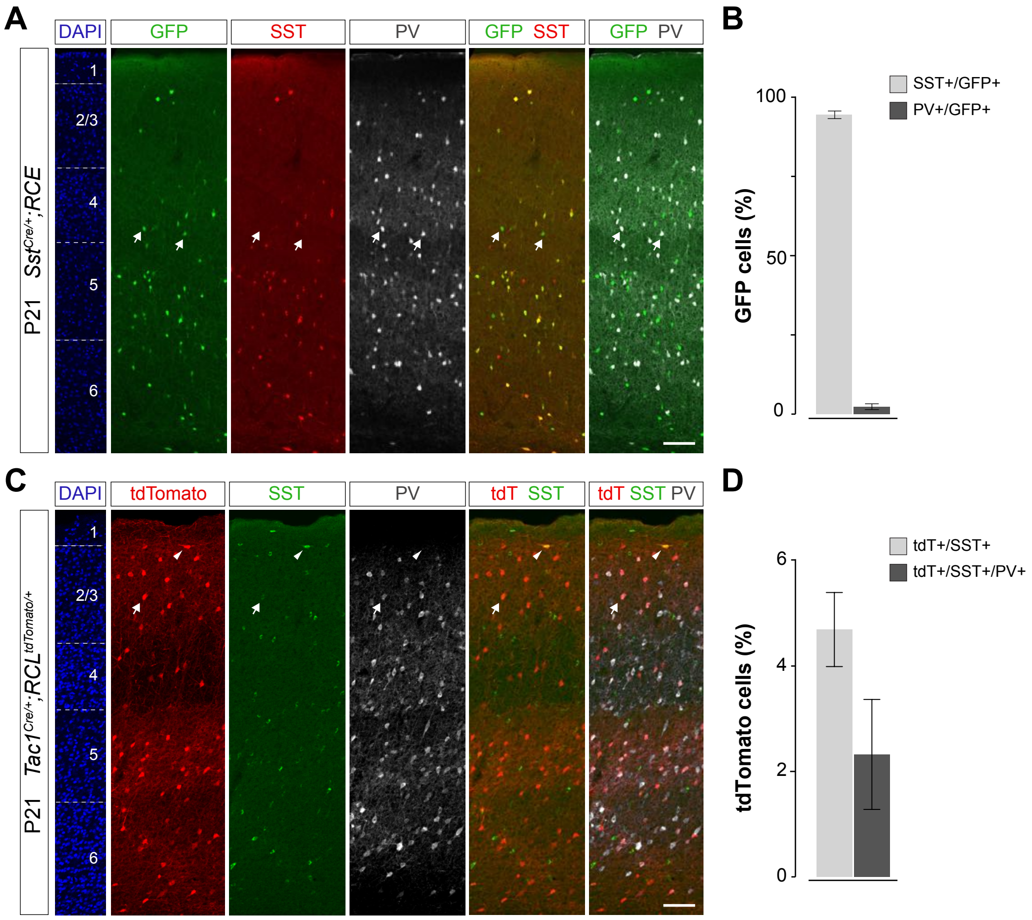
A
DAPI
GFP
SST
PV
GFP SST
GFP PV
P21 SstCre/+;RCE
1
2/3
4
5
6
B
SST+/GFP+
PV+/GFP+
GFP cells (%)
100
50
0
C
DAPI
tdTomato
SST
PV
tdT SST
tdT SST PV
P21 Tac1Cre/+;RCLtdTomato/+
D
tdT+/SST+
tdT+/SST+/PV+
tdTomato cells (%)
6
4
2
0

Figure S2

Figure S2. ***Sst*^{Cre}*;RCE* mice label SST+ neurons with great specificity, related to Figure 1.**
(A) Coronal sections through the neocortex of *Sst*$^{Cre/+}$*;RCE* mice at P21 stained with antibodies against GFP, SST, and PV. DAPI staining reveals the distribution of nuclei. Arrowheads indicate GFP+/PV+/SST- cells.
(B) Quantification of the fraction of SST+/GFP+ and PV+/GFP+ cells among GFP+ cells in the somatosensory cortex of *Sst*$^{cre/+}$*;RCE* mice (n = 4 mice) at P21.
(C) Coronal sections through the neocortex of *Tac1*$^{Cre/+}$*;RCL*$^{tdTomato/+}$ mice at P21 stained with antibodies against tdTomato, SST, and PV. DAPI staining reveals the distribution of nuclei. Arrowheads indicate a tdT+ SST+ PV- cell; arrows indicate a tdT+ SST+ PV+ cell.
(D) Quantification of the fraction of SST+/tdT+ and PV+/SST+/tdT+ cells among tdT+ cells in the somatosensory cortex of *Tac1*$^{Cre/+}$*;RCL*$^{tdTomato/+}$ mice (n = 2 mice) at P21.
Data are shown as mean ± s.e.m. Scale bars, 50 μm.

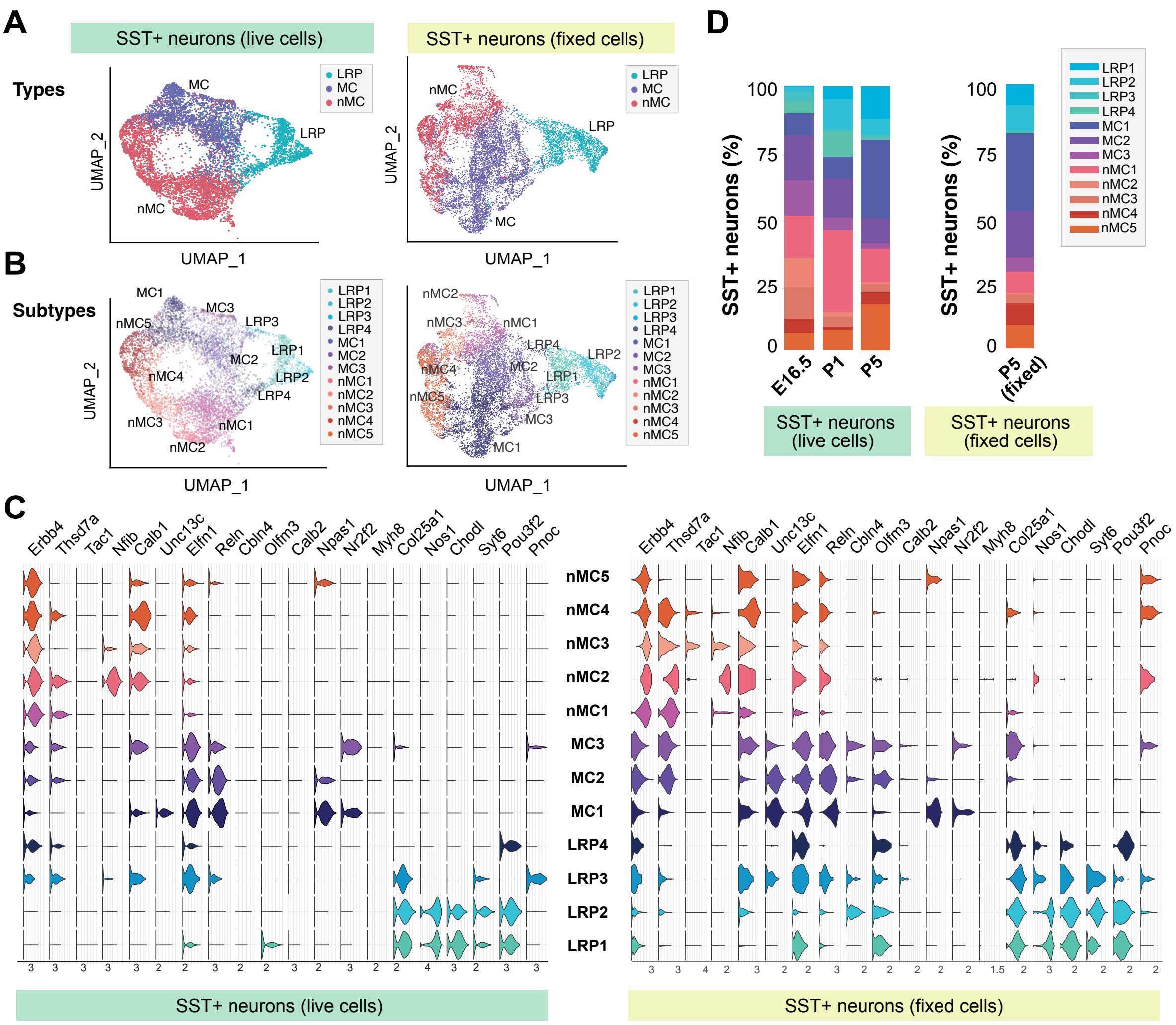
A
SST+ neurons (live cells)
SST+ neurons (fixed cells)
Types
B
Subtypes
C
D
SST+ neurons (%)
E16.5
P1
P5
P5 (fixed)
SST+ neurons (live cells)
SST+ neurons (fixed cells)
UMAP_1
UMAP_2
LRP
MC
nMC
LRP1
LRP2
LRP3
LRP4
MC1
MC2
MC3
nMC1
nMC2
nMC3
nMC4
nMC5
Erbb4
Thsd7a
Tac1
Nfib
Calb1
Unc13c
Elfn1
Reln
Cbln4
Olfm3
Calb2
Npas1
Nr2f2
Myh8
Col25a1
Nos1
Chodl
Syt6
Pou3f2
Pnoc

Figure S3

Figure S3. SST+ subtype diversity of live-FACS cells matches well with fixed-FACS cells at P5, related to Figure 2.

(A and B) Clustering of cortical SST+ cells into types (A) and subtypes (B) in the E16.5, P1 and P5 dataset of live-FACS cells and the P5 fixed-FACS. In the latter case, clusters were reannotated using the classifier models described in Figure 1.

(C) Distribution of each subtype in the E16.5, P1, and P5 dataset (live-FACS cells) and the fixed-FACS P5 dataset.

(D) Violin plots of selected cell type markers comparing SST+ cells in the E16.5, P1, and P5 dataset (live-FACS) and the fixed-FACS P5 dataset.

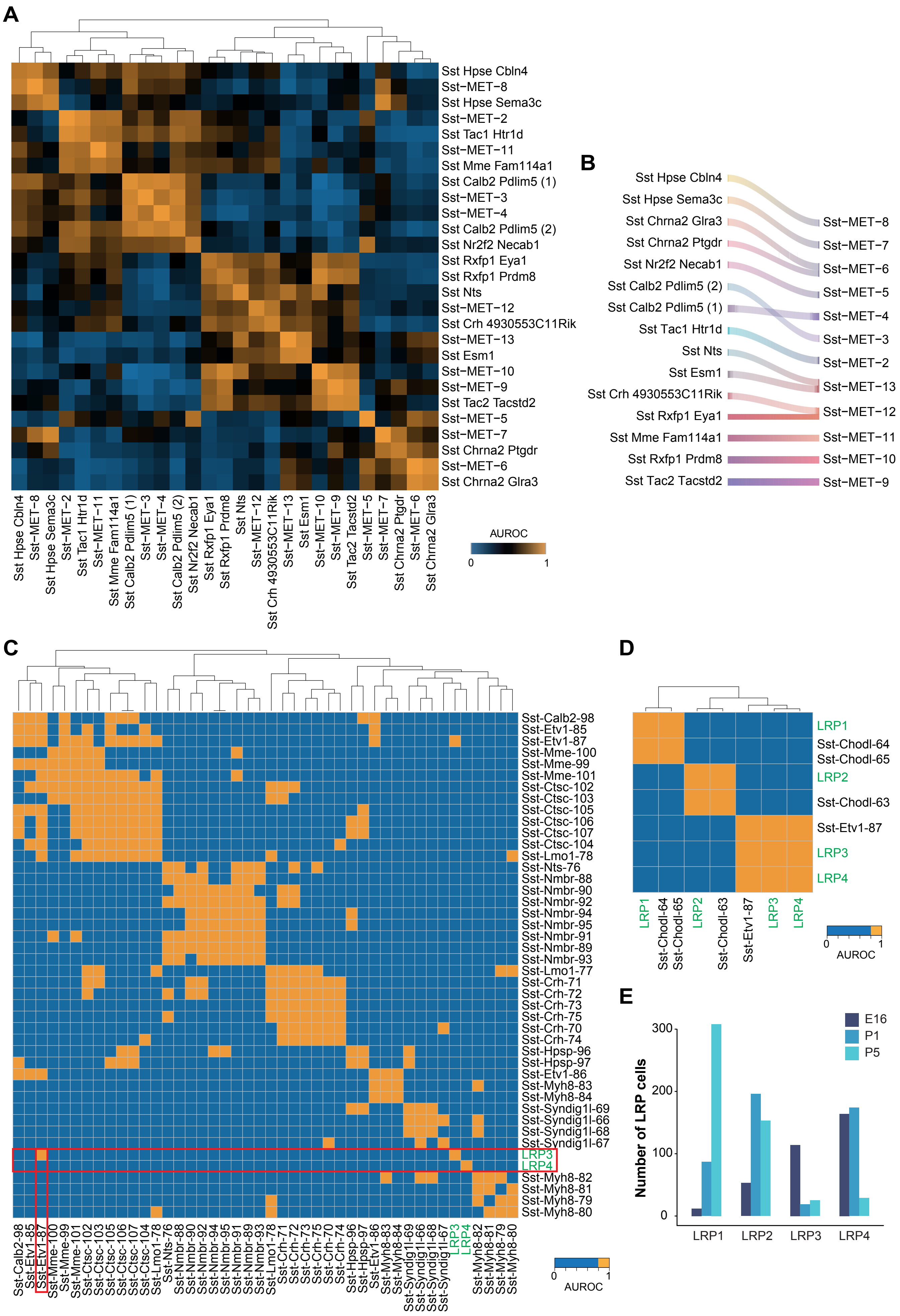
A
Sst Hpse Cbln4
Sst−MET−8
Sst Hpse Sema3c
Sst−MET−2
Sst Tac1 Htr1d
Sst−MET−11
Sst Mme Fam114a1
Sst Calb2 Pdlim5 (1)
Sst−MET−3
Sst−MET−4
Sst Calb2 Pdlim5 (2)
Sst Nr2f2 Necab1
Sst Rxfp1 Eya1
Sst Rxfp1 Prdm8
Sst Nts
Sst−MET−12
Sst Crh 4930553C11Rik
Sst−MET−13
Sst Esm1
Sst−MET−10
Sst−MET−9
Sst Tac2 Tacstd2
Sst−MET−5
Sst−MET−7
Sst Chrna2 Ptgdr
Sst−MET−6
Sst Chrna2 Glra3
AUROC
0
1
B
Sst Hpse Cbln4
Sst Hpse Sema3c
Sst Chrna2 Glra3
Sst Chrna2 Ptgdr
Sst Nr2f2 Necab1
Sst Calb2 Pdlim5 (2)
Sst Calb2 Pdlim5 (1)
Sst Tac1 Htr1d
Sst Nts
Sst Esm1
Sst Crh 4930553C11Rik
Sst Rxfp1 Eya1
Sst Mme Fam114a1
Sst Rxfp1 Prdm8
Sst Tac2 Tacstd2
Sst−MET−8
Sst−MET−7
Sst−MET−6
Sst−MET−5
Sst−MET−4
Sst−MET−3
Sst−MET−2
Sst−MET−13
Sst−MET−12
Sst−MET−11
Sst−MET−10
Sst−MET−9
C
Sst-Calb2-98
Sst-Etv1-85
Sst-Etv1-87
Sst-Mme-100
Sst-Mme-99
Sst-Mme-101
Sst-Ctsc-102
Sst-Ctsc-103
Sst-Ctsc-105
Sst-Ctsc-106
Sst-Ctsc-107
Sst-Ctsc-104
Sst-Lmo1-78
Sst-Nts-76
Sst-Nmbr-88
Sst-Nmbr-90
Sst-Nmbr-92
Sst-Nmbr-94
Sst-Nmbr-95
Sst-Nmbr-91
Sst-Nmbr-89
Sst-Nmbr-93
Sst-Lmo1-77
Sst-Crh-71
Sst-Crh-72
Sst-Crh-73
Sst-Crh-75
Sst-Crh-70
Sst-Crh-74
Sst-Hpsp-96
Sst-Hpsp-97
Sst-Etv1-86
Sst-Myh8-83
Sst-Myh8-84
Sst-Syndig1l-69
Sst-Syndig1l-66
Sst-Syndig1l-68
Sst-Syndig1l-67
LRP3
LRP4
Sst-Myh8-82
Sst-Myh8-81
Sst-Myh8-79
Sst-Myh8-80
AUROC
0
1
D
LRP1
Sst-Chodl-64
Sst-Chodl-65
LRP2
Sst-Chodl-63
Sst-Etv1-87
LRP3
LRP4
AUROC
0
1
E
E16
P1
P5
Number of LRP cells
300
200
100
0
LRP1
LRP2
LRP3
LRP4

Figure S4

Figure S4. MetaNeighbor analyses between adult and developmental datasets, related to Figure 3.

(A) Heatmap illustrating transcriptomic similarities (AUROC values) between adult transcriptomic SST+ cell clusters[20], and adult SST+ MET subtypes[2].

(B) River plot illustrating the correspondence of adult transcriptomic SST+ cell clusters and Sst-MET types using an AUROC value larger than 0.75.

(C) MetaNeighbor analysis illustrating transcriptomic similarities (AUROC values) between the developing SST+ clusters LRP3 and LRP4 and all adult transcriptomic SST+ subtypes identified by Yao et al. (2021)[29].

(D) MetaNeighbor analysis illustrating transcriptomic similarities (AUROC values) between LRP1 to 4 clusters and four adult Sst subtypes (Sst-Chodl-63, Sst-Chodl-64, Sst-Chodl-65, Sst-Etv1-87).

(E) Histograms illustrating the distribution of SST+ LRP cell clusters by developmental stage.

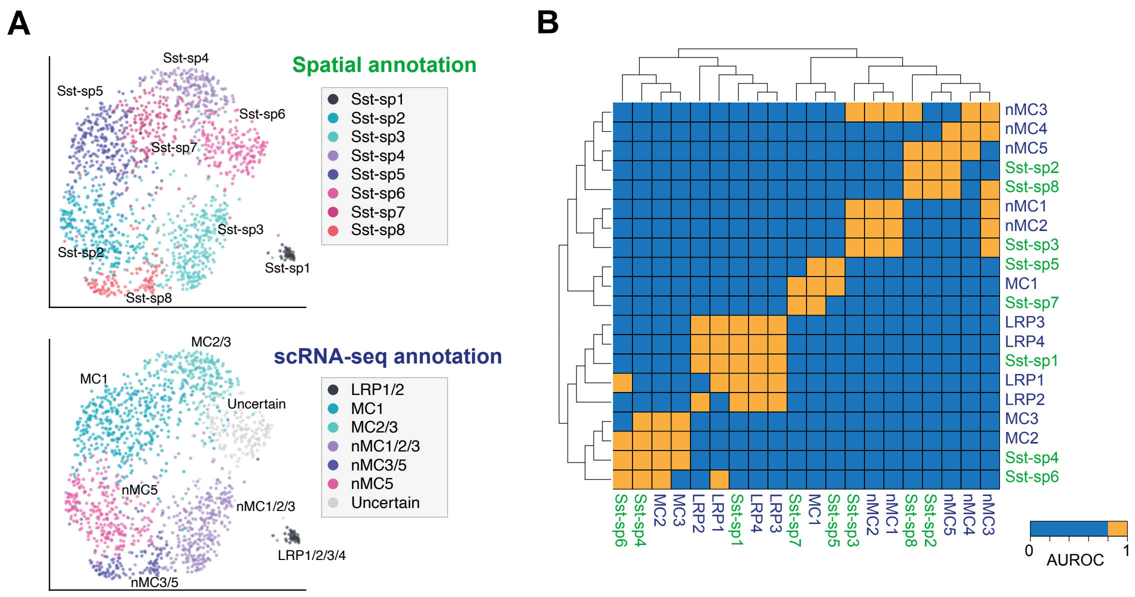
A
Spatial annotation
Sst-sp1
Sst-sp2
Sst-sp3
Sst-sp4
Sst-sp5
Sst-sp6
Sst-sp7
Sst-sp8
scRNA-seq annotation
LRP1/2
MC1
MC2/3
nMC1/2/3
nMC3/5
nMC5
Uncertain
LRP1/2/3/4
B
nMC3
nMC4
nMC5
Sst-sp2
Sst-sp8
nMC1
nMC2
Sst-sp3
Sst-sp5
MC1
Sst-sp7
LRP3
LRP4
Sst-sp1
LRP1
LRP2
MC3
MC2
Sst-sp4
Sst-sp6
AUROC
0
1

Figure S5

Figure S5. Alignment of scRNA-seq and spatial transcriptomics datasets, related to Figure 5.

(A) Integration of P5 cortical SST+ cells from the spatial transcriptomic (top panel) and scRNA-seq (bottom panel) datasets, and visualization by UMAP. Annotation of scRNA-seq clusters was based on marker expression, while annotation of spatial clusters was based on label transfer from the scRNA-seq dataset.

(B) MetaNeighbor analysis illustrating transcriptomic similarities between P5 scRNA-seq and spatial transcriptomic clusters.

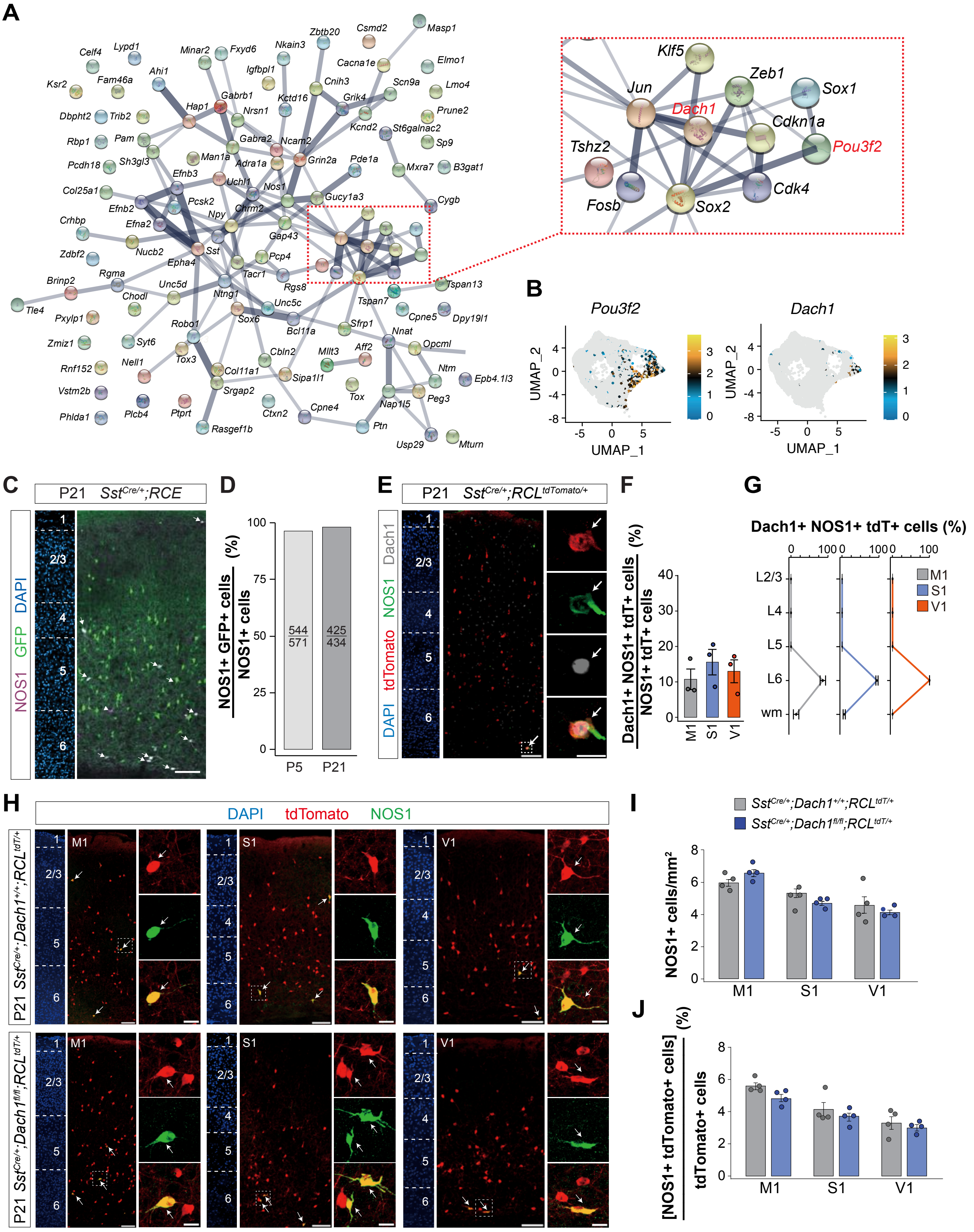
A
Klf5
Jun
Zeb1
Sox1
Dach1
Cdkn1a
Tshz2
Pou3f2
Fosb
Sox2
Cdk4
B
Pou3f2
Dach1
UMAP_1
UMAP_2
C
P21 SstCre/+;RCE
NOS1 GFP DAPI
D
NOS1+ GFP+ cells / NOS1+ cells (%)
544/571
425/434
P5
P21
E
P21 SstCre/+;RCLtdTomato/+
DAPI tdTomato NOS1 Dach1
F
Dach1+ NOS1+ tdT+ cells / NOS1+ tdT+ cells (%)
M1
S1
V1
G
Dach1+ NOS1+ tdT+ cells (%)
L2/3
L4
L5
L6
wm
H
DAPI tdTomato NOS1
P21 SstCre/+;Dach1+/+;RCLtdT/+
P21 SstCre/+;Dach1fl/fl;RCLtdT/+
I
SstCre/+;Dach1+/+;RCLtdT/+
SstCre/+;Dach1fl/fl;RCLtdT/+
NOS1+ cells/mm2
J
[NOS1+ tdTomato+ cells] / tdTomato+ cells (%)

Figure S6

Figure S6. Genes differentially expressed genes in LRP neurons and function of Dach1 in LRP development, related to Figures 6 and 7.

(A) STRING network analysis for genes differentially expressed between SST+ LRP neurons and SST+ interneurons (Table S3). Full STRING network, medium confidence (0.4), size cut-off of no more than ten interactors. The inset highlights a cluster of transcription factors at the core of this network.

(B) The feature plots depict the expression of two genes, *Dach1* and *Pou3f2*, which are highly enriched and almost exclusively expressed by LRP neurons.

(C) Coronal sections through the neocortex of $Sst^{Cre/+}$*;RCE* mice P21 stained with antibodies against NOS1 and GFP. DAPI staining reveals the distribution of nuclei.

(D) Quantification of the fraction of NOS1+ and GFP+ cells among GFP+ cells in the neocortex of $Sst^{Cre/+}$*;RCE* mice at P5 and P21 (*n* = 3 mice for each stage).

(E) Expression of Dach1 in cortical LRP neurons in primary motor cortex (M1), primary somatosensory cortex (S1), and primary visual cortex (V1) of $Sst^{Cre/+};RCL^{tdT/+}$ mice at P21 stained with antibodies against NOS1, Dach1, and tdTomato. DAPI staining reveals the distribution of nuclei.

(F) Quantification of the fraction of NOS1+ and Dach1+ cells among cortical SST+ neurons (*n* = 3 mice). Student *t*-test: not significant.

(G) Laminar distribution of Dach1+/NOS1+/tdTomato+ cells in M1, S1 and V1 of $Sst^{Cre/+};RCL^{tdT/+}$ mice at P21 (*n* = 3 mice).

(H) Coronal sections through the neocortex of control and *Dach1* conditional mutant mice at P21 stained with antibodies against NOS1 and tdTomato. Arrows indicate NOS1+ and tdTomato+ cells. DAPI staining reveals the distribution of nuclei.

(I and J) Quantification of the density and proportion of NOS1+ and tdTomato+ neurons in M1, S1, and V1 of control and *Dach1* conditional mutant mice at P21 (*n* = 4 mice per genotype). Two-way ANOVA: not significant.

Data are shown as mean ± s.e.m. Scale bars, 100 µm and 20 µm (inserts).

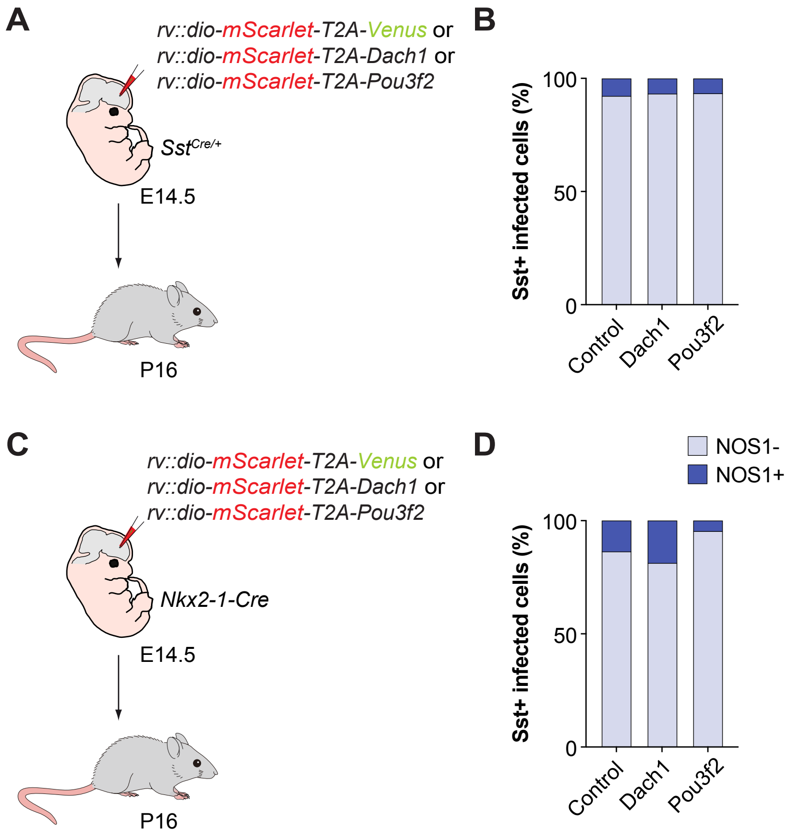
A
rv::dio-mScarlet-T2A-Venus or
rv::dio-mScarlet-T2A-Dach1 or
rv::dio-mScarlet-T2A-Pou3f2
SstCre/+
E14.5
P16
B
Sst+ infected cells (%)
100
50
0
Control
Dach1
Pou3f2
C
rv::dio-mScarlet-T2A-Venus or
rv::dio-mScarlet-T2A-Dach1 or
rv::dio-mScarlet-T2A-Pou3f2
Nkx2-1-Cre
E14.5
P16
D
NOS1-
NOS1+
Sst+ infected cells (%)
100
50
0
Control
Dach1
Pou3f2

Figure S7

Figure S7. Expression of Dach1 and Pou3f2 does not induce NOS1 expression, related to Figures 6 and Figure 7.
(A) Schematic of the experimental design.
(B) Quantification of the fraction of NOS1- and NOS1+ cells among infected mScarlet+ cells in the neocortex of *Sst*$^{Cre/+}$ mice at P16. Control (*mScarlet-T2A-mVenus*: 192 NOS1- cells and 17 NOS1+ cells); *mScarlet-T2A-Dach1*: 175 NOS1- cells and 13 NOS1+ cells; *mScarlet-T2A-Pou3f2*: 149 NOS1- cells and 11 NOS1+ cells. Chi-square test: $p = 0.8635$.
(C) Schematic of the experimental design.
(D) Quantification of the fraction of NOS1- and NOS1+ cells among infected mScarlet+ cells in the neocortex of *Nkx2-1-Cre/+* injected animals at P16. Control (*mScarlet-T2A-mVenus*: 78 NOS1- cells and 13 NOS1+ cells; *mScarlet-T2A-Dach1*: 50 NOS1- cells and 13 NOS1+ cells; *mScarlet-T2A-Pou3f2*: 20 NOS1- cells and 1 NOS1+ cells. Chi-square test: $p = 0.1968$.

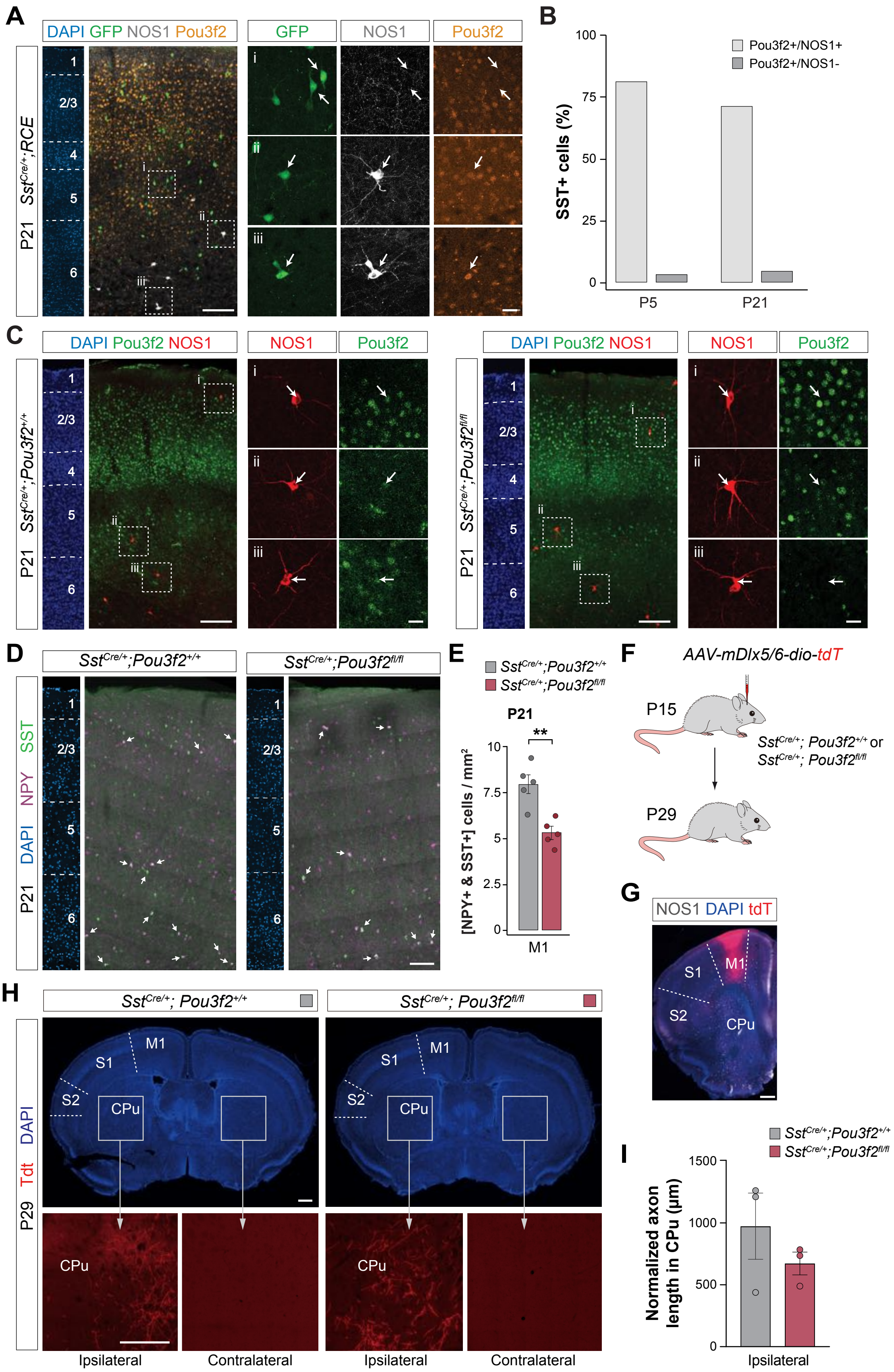
A
DAPI GFP NOS1 Pou3f2
GFP
NOS1
Pou3f2
P21 SstCre/+;RCE
B
SST+ cells (%)
Pou3f2+/NOS1+
Pou3f2+/NOS1-
P5
P21
C
DAPI Pou3f2 NOS1
P21 SstCre/+;Pou3f2+/+
P21 SstCre/+;Pou3f2fl/fl
D
SstCre/+;Pou3f2+/+
SstCre/+;Pou3f2fl/fl
P21 DAPI NPY SST
E
P21
[NPY+ & SST+] cells / mm2
M1
F
AAV-mDlx5/6-dio-tdT
P15
P29
SstCre/+; Pou3f2+/+ or
SstCre/+; Pou3f2fl/fl
G
NOS1 DAPI tdT
H
P29 Tdt DAPI
Ipsilateral
Contralateral
I
Normalized axon length in CPu (µm)
Ipsilateral

Figure S8

Figure S8. Expression of Pou3f2 in cortical LRP neurons, related to Figure 7.
(A) Coronal sections through the neocortex of *Sst*$^{Cre/+}$*;RCE* mice at P5 and P21 stained with antibodies against NOS1, Pou3f2, and GFP. DAPI staining reveals the distribution of nuclei.
(B) Quantification of the fraction of Pou3f2+/NOS1+ cells and Pou3f2+/NOS1- cells among cortical SST+ neurons.
(C) Coronal sections through the neocortex of control and *Pou3f2* conditional mutant mice at P21 stained with antibodies against NOS1 and Pou3f2. DAPI staining reveals the distribution of nuclei.
(D) Coronal sections through the mouse telencephalon stained with antibodies against NPY and SST in control and *Pouf3f2* conditional mutants at P21. DAPI staining reveals the distribution of nuclei.
(E) Quantification of the density of SST+/NPY+ cells in the motor cortex at P21 (n = 5 per genotype). Student t-test with Bonferroni: $^{**}p < 0.01$.
(F) Schematic of the experimental design for axonal labeling.
(G) Coronal section through the injection site stained with antibodies against tdTomato and NOS1. DAPI staining reveals the distribution of nuclei.
(H) Coronal sections through the mouse telencephalon stained with antibodies against tdTomato in control and *Pou3f2* conditional mutants at P29. DAPI staining reveals the distribution of nuclei. The high-magnification images illustrate axons in the striatum (CPu).
(I) Quantification of total axonal length in the ipsilateral striatum normalized by the number of NOS1+/tdTomato+ cells in each animal (n = 3 mice per genotype).
Data are shown as mean ± s.e.m. Scale bars, 500 µm (G and H), 100 µm (A, C and D), and 20 µm (insets).

Table S1. Differentially expressed genes among SST+ neurons, related to Figure 1

Marker Gene	Cell type Enrichment	Marker Gene	Cell type Enrichment
CHRNA2	Martinotti	SFRP1	LRP
CALB2	Martinotti	POU3F2	LRP
NPAS1	Martinotti	DACH1	LRP
CBLN4	Martinotti	SYT6	LRP
MYH8	Martinotti	HHIP	LRP
HTR1A	Martinotti	CHODL	LRP
ELFN1	Martinotti	NOS1	LRP
GPX3	Martinotti	CHRM2	LRP
NR2F2	Martinotti	PTN	LRP
SATB1	Martinotti	COL25A1	LRP
ETV1	Martinotti	NPY	LRP
HPSE	Martinotti	CRHBP	LRP
RELN	Martinotti	TRPM3	LRP
UNC13C	Martinotti	CPNE4	LRP
TMEFF2	Martinotti	EGFEM1	LRP
DPP10	Martinotti	CLU	Stressed
OLFM3	Martinotti	APP	Stressed
KIRREL3	Martinotti	CPE	Stressed
NELL1	Martinotti	PSAP	Stressed
HS6ST3	Martinotti	PDIA6	Stressed
PCDH7	Martinotti	ATP2C1	Stressed
CDH12	Martinotti	HSPA5	Stressed
NTS	Non-Martinotti	CALR	Stressed
TAC2	Non-Martinotti	P4HB	Stressed
TAC1	Non-Martinotti	HSP90B1	Stressed
TH	Non-Martinotti	XBP1	Stressed
PTPRD	Non-Martinotti	CDH13	Stressed
C1QL3	Non-Martinotti		
CRH	Non-Martinotti		
THSD7A	Non-Martinotti		
ERBB4	Non-Martinotti		
GABRA1	Non-Martinotti		
KCNH7	Non-Martinotti		
CNTNAP2	Non-Martinotti		
LRP1B	Non-Martinotti		
KCNIP4	Non-Martinotti		
CNTN5	Non-Martinotti		
CSMD1	Non-Martinotti		
GABRG3	Non-Martinotti		
LRRC4C	Non-Martinotti		

Table S2. Genes in developmental modules, related to Figure 4

**Gene symbol**	**Gene symbol**	**Gene symbol**	**Gene symbol**
2900055J20RIK	ABLIM1	GNG4	SLC24A2
AHI1	ABLIM3	GRIN1	SLC24A3
ARFGEF3	ACHE	GRIN2A	SNAP91
ATP6V0A1	ADCY1	GRIN2B	SNCA
B230209E15RIK	ADCY2	GRIN3A	SPARCL1
CDKN1A	ADCYAP1R1	IGF1	SRGAP3
CLCN3	AF529169	ILDR2	SSX2IP
CPNE5	AI413582	INPP5F	STUM
CRHBP	AI504432	KCNMA1	SV2A
DLG2	AKAP7	KCNQ3	SYNGR1
FXYD6	ALCAM	KIF1A	SYNPR
GABRB1	AP2A2	KIF5A	SYP
GAP43	ATP1A3	KIFC2	SYT13
GRM1	ATP1B1	MAP1A	THRA
HAP1	ATP2A2	MAP2K4	TMEM151A
KCND2	B4GALT6	MAP6	TMOD2
L1CAM	BEND6	MAPK10	TRIM37
LSAMP	CACNG2	MRFAP1	TRPC5
MEG3	CADM2	MTPN	TSPAN17
NCAM2	CADM3	NAV2	UNC13C
NRXN1	CAMK2B	NCDN	YWHAB
NTM	CAMK2N1	NDRG4	YWHAZ
OPCML	CAMK4	NPTXR	
PCLO	CBARP	NRGN	
PEG3	CD99L2	NRIP3	
PFKP	CDK5R2	NRXN2	
PKIA	CDKL2	PAFAH1B1	
PLPPR4	CLSTN1	PDYN	
PSAP	CNTN1	PEA15A	
PTPRS	CTNND2	PGM2L1	
RIMS1	CX3CL1	PITPNC1	
SCN3B	DNAJC5	PPP2R2C	
SEZ6L2	DPYSL5	PRKCG	
SNHG11	EIF4G2	PTPRN	
SPOCK2	EMC10	RASGRF2	
STXBP6	ENO2	REEP5	
SYN1	FAM171B	RELL2	
SYT11	FKBP1B	RGS6	
SYT4	FUT9	ROBO2	
TENM1	FXYD7	SCAMP5	
TIMP2	GABBR2	SCN2A	
TMEM130	GAS7	SDC3	
TSPAN7	GDA	SH3GL2	
1500009L16RIK	GNAL	SHISA6	
2900011O08RIK	GNAO1	SHROOM2	
2900097C17RIK	GNAZ	SLC12A5	

Table S4. Genes used in spatial transcriptomics experiments, related to Figure 5

Gene symbol	Gene symbol	Gene symbol	Gene symbol
ADRA1A	ERBB4	MYH8	RGS6
ALKAL2	ETV1	NDST4	RXFP1
AMELX	GABRA2	NETO1	SATB1
APELA	GAD1	NFIB	SFRP1
APP	GAD2	NOS1	SHISA9
B3GAT1	GNG4	NPAS1	SLC24A2
BCL6	GPHN	NPAS3	SLC30A3
BMP3	GPX3	NPY	SORCS1
CALB2	HAP1	NR2F2	SOX1
CBLN4	HHIP	PAX6	SP9
CDH8	HPSE	PBX3	SPON1
CHODL	ID2	PCP4	SST
CHRNA2	ILDR2	PCP4L1	SYP
CHRNA4	INHBB	PDE1A	SYT6
CLU	KCNIP2	PDYN	TAC1
COL19A1	KHDRBS3	POU3F2	TACR1
COL25A1	LHX6	PSAP	TACR3
CORT	LHX8	PTN	TH
DACH1	LRP1B	RARB	THSD7A
DBPHT2	LYPD1	RASGEF1B	TRIB2
DLX1	LYPD6	RBFOX3	UNC5C
DLX5	MEIS2	RELN	ZEB2
ELFN1	MGAT4C	RGMA	

Table S5. Summary of data and statistical analyses, related to Figures 5-8 and Figures S2, S6, S7 and S8

**Fig. 5**	Measurement	Values	N	Statistical	P value
Fig. 5E	Fraction of cells in superficial and deep layers	LRP1/2: 5.4 and 94.6; Sst-MET-1: 31.2 and 68.8; MC1: 2.5 and 97.5; Sst-MET-6/7: 0 and 100; MC2/3: 37.9 and 62.1; Sst-MET-3/4: 63.4 and 36.6; nMC1/2/3: 20.8 and 79.2; Sst-MET2: 88.2 and 11.8; nMC3/5: 0 and 100; Sst-MET-9/10/13: 0 and 100	[total cells] LRP1/2 = 37<br>Sst-MET-1 = 16<br>MC1 = 160<br>Sst-MET-6/7 = 29<br>MC2/3 = 211<br>Sst-MET-3/4 = 41<br>nMC1/2/3 = 312<br>Sst-MET2 = 17<br>nMC3/5 = 116<br>Sst-MET-9/10/13 = 40	Fisher exact test	LRP1/Sst-MET-1 = 0.029<br>MC1/2/Sst-MET-6/7 = 1<br>MC2/3/ Sst-MET-3/4 = 0.0032<br>nMC1/2/3/ Sst-MET-2 = 0.00001<br>nMC3/5/ Sst-MET-9/10/13 = 1
**Fig. 6**	Measurement	Values	N	Statistical	P value
Fig. 6D	Ipsilateral axon length per cell in CPu at P70 (mean ± SEM)	$Sst^{Cre/+};Dach1^{+/+}$: 104.74 ± 4.21;<br>$Sst^{Cre/+};Dach1^{fl/fl}$: 115.12 ± 5.94	[brains] n = 6 for each genotype	Unpaired t-test	p = 0.1844
Fig. 6D	Contralateral axon length per cell in CPu at P70 (mean ± SEM)	$Sst^{Cre/+};Dach1^{+/+}$: 0.78 ± 0.44;<br>$Sst^{Cre/+};Dach1^{fl/fl}$: 7.05 ± 1.78	[brains] n = 6 for each genotype	Unpaired t-test	p = 0.0066
Fig. 6E	Ipsilateral axon length per cell in CPu at p29 (mean ± SEM)	$Sst^{Cre/+};Dach1^{+/+}$: 106.62 ± 7.35;<br>$Sst^{Cre/+};Dach1^{fl/fl}$: *118.91* ± 4.27	[brains] n = 6 for each genotype	Unpaired t-test	p = 0.1789
Fig. 6E	Contralateral axon length per cell in CPu at P29 (mean ± SEM)	$Sst^{Cre/+};Dach1^{+/+}$: *0.14* ± 0.04;<br>$Sst^{Cre/+};Dach1^{fl/fl}$: *3.16* ± 0.46	[brains] n = 6 for each genotype	Unpaired t-test	p < 0.0001
**Fig. 7**	Measurement	Values	N	Statistical	P value
Fig. 7B	Mean Density of Nos1+ cells at P5 (mean ± SEM)	$Sst^{cre/+};Pou3f2^{+/+}$: M1, 12.69 ± 1.05; S1, 20.31 ± 1.36; V1, 12.21 ± 0.95.<br>$Sst^{Cre/+};Pou3f2^{fl/fl}$: M1, 13.69 ± 1.25; S1, 19.55 ± 1.25; V1, 12.39 ± 0.74	[brains] n = 5 for each genotype	t-test (Mean Density~Genotype), post-hoc by Bonferroni correction	M1, p = 1; S1, p = 1; V1, p = 1
Fig. 7B	Mean Density of Nos1+ cells at P21 (mean ± SEM)	$Sst^{Cre/+};Pou3f2^{+/+}$: M1, 8.17 ± 0.44; S1, 8.03 ± 0.35; V1, 6.87 ± 0.72.<br>$Sst^{Cre/+};Pou3f2^{fl/fl}$: M1, 5.61 ± 0.22; S1, 6.25 ± 0.32; V1, 4.45 ± 0.42	[brains] n = 6 for each genotype	t-test (Mean Density~Genotype), post-hoc by Bonferroni correction	M1, p = 0.0032; S1, p = 0.012; V1, p = 0.047
Fig. 7D	Normalized Mean Density of Nos1+ cells in MCx at P5, P10, P14 and P21 (mean ± SEM)	$Sst^{Cre/+};Pou3f2^{+/+}$: P5, 1 ± 0.08; P10, 1 ± 0.05; P14, 1 ± 0.03; P21, 1 ± 0.05.<br>$Sst^{Cre/+};Pou3f2^{fl/fl}$: P5, 1.07 ± 0.09; P10, 0.87 ± 0.04; P14, 0.80 ± 0.03; P21, 0.68 ± 0.02	[brains] n = 5 for each genotype at P5, 4 for each genotype at P10, 3 for each genotype at P14 and 6 for each genotype at P21	t-test (Mean Density~Genotype), post-hoc by Bonferroni correction	P5, p = 1; P10, p = 0.39; P14, p = 0.048; P21, p = 0.0032
Fig. 7D	Normalized Mean Density of Nos1+ cells in SSCx at P5, P10, P14 and P21 (mean ± SEM)	$Sst^{Cre/+};Pou3f2^{+/+}$: P5, 1 ± 0.06; P10, 1 ± 0.02; P14, 1 ± 0.01; P21, 1 ± 0.04.<br>$Sst^{Cre/+};Pou3f2^{fl/fl}$: P5, 0.96 ± 0.06; P10, 0.86 ± 0.04; P14, 0.77 ± 0.01; P21, 0.77 ± 0.04	[brains] n = 5 for each genotype at P5, 4 for each genotype at P10, 3 for each genotype at P14 and 6 for each genotype at P21	t-test (Mean Density~Genotype), post-hoc by Bonferroni correction	P5, p = 1; P10, p = 0.17; P14, p = 0.002; P21, p = 0.012
Fig. 7D	Normalized Mean Density of Nos1+ cells in VCx at P5, P10, P14 and P21 (mean ± SEM)	$Sst^{Cre/+};Pou3f2^{+/+}$: P5, 1 ± 0.07; P10, 1 ± 0.06; P14, 1 ± 0.07; P21, 1 ± 0.08.<br>$Sst^{Cre/+};Pou3f2^{fl/fl}$: P5, 0.99 ± 0.06; P10, 0.81 ± 0.05; P14, 0.71 ± 0.008; P21, 0.68 ± 0.05	[brains] n = 5 for each genotype at P5, 4 for each genotype at P10, 3 for each genotype at P14 and 6 for each genotype at P21	t-test (Mean Density~Genotype), post-hoc by Bonferroni correction	P5, p = 1; P10, p = 0.23; P14, p = 0.18; P21, p = 0.047
**Fig. 8**	Measurement	Values	N	Statistical	P value
Fig. 8B	Sox2 level intenstiy (mean ± SEM)	$Sst^{Cre/+;}RCE$: SST+NOS1+, 20,340 ± 762.8; SST+;NOS1-, 3,184 ± 76.1	[brains] n= 3	Unpaired t-test	p < 0.00001
Fig. 8D	Sox2 level intensity at P5 (mean ± SEM)	$Sst^{Cre/+};Pou3f2^{+/+}$: NOS1+: 1223 ± 23;<br>$Sst^{Cre/+};Pou3f2^{fl/fl}$: NOS1+: 899 ± 60	[brains] n = 3 for each genotype	Unpaired t-test	p = 0.0075
Fig. 8E	Normalized comparative Ct: Pou3f2, Pou3f3, Sox2 at P5 (%) (mean ± SEM)	$Sst^{Cre/+};Pou3f2^{+/+}$: Pou3f2, 100; Pou3f3, 100; Sox2, 100.<br>$Sst^{Cre/+};Pou3f2^{fl/fl}$: Pou3f2, 40.5 ± 0.5; Pou3f3, 125.5 ± 11.5; Sox2, 70.5 ± 11.5	[brains] n = 3 for each genotype	Unpaired t-test	Pou3f2: p = 0.00047; Pou3f3, p = 0.25; Sox2, p = 0.0015
**Fig. S2**	Measurement	Values	N	Statistical	P value
Fig. S2B	GFP+SST+ cells and GFP+PV+ cells at P21 (%)	GFP+SST+ cells: 90.2 ± 2.0%; GFP+PV+ cells: 2.6 ± 1.4%	[brains] n = 4	N/A	
Fig. S2D	tdTomato+SST+ cells and tdTomato+SST+PV+ cells at P21 (%)	tdT+SST+ cells: 4.6 ± 0.7%; tdT+SST+ PV+ cells: 2.32 ± 1.04%	[brains] n = 2	N/A	
**Fig. S6**	Measurement	Values	N	Statistical	P value

Fig. S6D	Fraction of NOS1+ cells expressing SST at P5 and P21 (%)	*Sst* $^{Cre/+}$*;RCE*: P5, 95.27; P21, 97.92	[cells] n = 571 Nos1+, 544 Nos1+; GFP+ at P5 and n =434 Nos1+, 425 Nos1+;GFP+ at P21	N/A	
Fig. S6F	Proportion of Dach1+;Nos+;tdTomato+ cells among Nos+;tdTomato cells at P21 (%) (mean ± SEM)	*Sst* $^{Cre/+}$*;RCL* $^{tdT/+}$: M1, 10.73 ± 2.90; S1, 15.57 ± 3.60; V1, 12.99 ± 3.23	[brains] n=3 for each cortical area	One-way ANOVA, Bonferroni's multiple comparisons test	M1 vs. S1 : *p* = 0.9998<br>M1 vs. V1 : p > 0.9999<br>S1 vs. V1 : p > 0.9999
Fig. S6G	Ratio of Dach1+;Nos1+;tdTomato+ cells in each layer at P21 (%) (mean ± SEM)	*Sst* $^{Cre/+}$*;RCL* $^{tdT/+}$: M1, L2/3: 0%; L4: 0%; L5: 0%; L6: 91.41% ± 4.82%; WM: 8.59% ± 4.82%; S1, L2/3: 0%; L4: 0%; L5: 0%; L6: 91.90% ± 4.23%; WM: 8.10% ± 4.23%; V1, L2/3: 0%; L4: 0%; L5: 0%; L6: 100.00% ; WM: 0%.	[brains] n=3 for each cortical area	N/A	
Fig. S6I	Mean Density of NOS1+ cells at P21 (mean ± SEM)	*Sst* $^{Cre/+}$*;Dach1* $^{+/+}$: M1, 6.02 ± 0.24; S1, 5.07 ± 0.31; V1, 4.62 ± 0.59.<br>*Sst* $^{Cre/+}$*;Dach1* $^{fl/fl}$: M1, 6.24 ± 0.46; S1, 4.60 ± 0.15; V1, 4.26 ± 0.14	[brains] n = 4 for each genotype	Two-way ANOVA, Bonferroni's multiple comparisons test	M1 : *p* = 0.4240<br>S1 : *p* = 0.5307<br>V1 : *p* > 0.9999
Fig. S6J	Proportion of NOS1+;tdTomato+ cells at P21 (%) (mean ± SEM)	*Sst* $^{Cre/+}$*;Dach1* $^{+/+}$: M1, 5.63 ± 0.22; S1, 4.12 ± 0.46; V1, 3.31 ± 0.41.<br>*Sst* $^{Cre/+}$*;Dach1* $^{fl/fl}$: M1, 4.87 ± 0.23; S1, 3.65 ± 0.25; V1, 3.01 ± 0.18	[brains] n = 4 for each genotype	Two-way ANOVA, Bonferroni's multiple comparisons test	M1 : *p* = 0.3057<br>S1 : *p* = 0.9091<br>V1 : *p* > 0.9999
**Fig. S7**	Measurement	Values	N	Statistical	P value
Fig. S7B	Fraction of infected mScarlet+ cells expressing NOS1 at P16 (%)	Control: 91.87% NOS1-, 8.13% NOS1+; Dach1: 93.09% NOS1-, 6.91% NOS1+; Pou3f2: 93.13% NOS1-, 6.87% NOS1+	[cells] Control: n = 192 Nos1- and n = 17 Nos1+; Dach1: n = 175 Nos1- and n = 13 Nos1+; Pou3f2: n = 149 Nos1- and n = 11 Nos1+.	Chi-square test	*p* = 0.8635
Fig. S7D	Fraction of infected mScarlet+ PV- cells expressing NOS1 at P16 (%)	Control: 85.71% NOS1-, 14.29% NOS1+; Dach1: 79.37% NOS1-, 20.63% NOS1+; Pou3f2: 95.24% NOS1-, 4.76% NOS1+	[cells] Control: n = 78 Nos1- and n = 13 Nos1+; Dach1: n = 50 Nos1- and n = 13 Nos1+; Pou3f2: n = 20 Nos1- and n = 1 Nos1+;	Chi-square test	*p* = 0.1988
**Fig. S8**	Measurement	Values	N	Statistical	P value
Fig. S8B	Fraction of NOS1+;SST+ cells or SST+ cells expressing Pou3f2 at P5 and P21 (%)	*Sst* $^{Cre/+}$*;RCE:* NOS1+SST+, P5, 81.19; P21, 70.90. SST+ P5, 3.13; P21: 4.34.	[cells] n = 1787 SST+ and n = 234 Nos1+ at P5 and n = 899 SST+ and n = 110 Nos1+ at P21	N/A	
